# Development and validation of a race-agnostic computable phenotype for kidney health in adult hospitalized patients

**DOI:** 10.1371/journal.pone.0299332

**Published:** 2024-04-23

**Authors:** Tezcan Ozrazgat-Baslanti, Yuanfang Ren, Esra Adiyeke, Rubab Islam, Haleh Hashemighouchani, Matthew Ruppert, Shunshun Miao, Tyler Loftus, Crystal Johnson-Mann, R. W. M. A. Madushani, Elizabeth A. Shenkman, William Hogan, Mark S. Segal, Gloria Lipori, Azra Bihorac, Charles Hobson

**Affiliations:** 1 University of Florida Intelligent Clinical Care Center (IC3), Gainesville, Florida, United States of America; 2 Department of Medicine, College of Medicine, University of Florida, Gainesville, Florida, United States of America; 3 Department of Surgery, College of Medicine, University of Florida, Gainesville, Florida, United States of America; 4 University of Florida Health Outcomes and Biomedical Informatics, University of Florida, Gainesville, Florida, United States of America; 5 University of Florida Health, Gainesville, Florida, United States of America; 6 Department of Health Services Research, Management and Policy, College of Public Health and Health Professions, University of Florida, Gainesville, Florida, United States of America; Istanbul University-Cerrahpasa, Cerrahpasa Medical Faculty, TURKEY

## Abstract

Standard race adjustments for estimating glomerular filtration rate (GFR) and reference creatinine can yield a lower acute kidney injury (AKI) and chronic kidney disease (CKD) prevalence among African American patients than non–race adjusted estimates. We developed two race-agnostic computable phenotypes that assess kidney health among 139,152 subjects admitted to the University of Florida Health between 1/2012–8/2019 by removing the race modifier from the estimated GFR and estimated creatinine formula used by the race-adjusted algorithm (*race-agnostic algorithm 1)* and by utilizing 2021 CKD-EPI refit without race formula (*race-agnostic algorithm 2*) for calculations of the estimated GFR and estimated creatinine. We compared results using these algorithms to the race-adjusted algorithm in African American patients. Using clinical adjudication, we validated race-agnostic computable phenotypes developed for preadmission CKD and AKI presence on 300 cases. Race adjustment reclassified 2,113 (8%) to no CKD and 7,901 (29%) to a less severe CKD stage compared to race-agnostic algorithm 1 and reclassified 1,208 (5%) to no CKD and 4,606 (18%) to a less severe CKD stage compared to race-agnostic algorithm 2. Of 12,451 AKI encounters based on race-agnostic algorithm 1, race adjustment reclassified 591 to No AKI and 305 to a less severe AKI stage. Of 12,251 AKI encounters based on race-agnostic algorithm 2, race adjustment reclassified 382 to No AKI and 196 (1.6%) to a less severe AKI stage. The phenotyping algorithm based on refit without race formula performed well in identifying patients with CKD and AKI with a sensitivity of 100% (95% confidence interval [CI] 97%–100%) and 99% (95% CI 97%–100%) and a specificity of 88% (95% CI 82%–93%) and 98% (95% CI 93%–100%), respectively. Race-agnostic algorithms identified substantial proportions of additional patients with CKD and AKI compared to race-adjusted algorithm in African American patients. The phenotyping algorithm is promising in identifying patients with kidney disease and improving clinical decision-making.

## Introduction

The advent of the electronic health record (EHR) has transformed clinical care and our ability to analyze that care [1]. Electronic or computable phenotyping identifies and characterizes clinical conditions through automated queries of digital health records [2, 3]. Acute kidney injury (AKI) and chronic kidney disease (CKD) are clinically used categorizations of kidney health that may be recognized as related entities and a continuum of the disease process [4] and are ideal targets for computational phenotyping, because this would enable a comprehensive, time-efficient, and consistent evaluation of kidney health status and help healthcare providers to save time in the evaluation and management process and to improve outcomes. Hospitalized patients with AKI have up to five-fold increases in risk for other serious complications and an increase in hospital cost of up to $28,000 per hospitalization; CKD-related expenditures exceed $48 billion per year [5]. Both AKI and CKD are frequently asymptomatic at their early stages [6, 7]. Delayed recognition and treatment of CKD and AKI are associated with adverse clinical outcomes, including kidney failure, cardiovascular disease, and higher mortality risk [8, 9].

The Kidney Disease: Improving Global Outcomes (KDIGO) Consortium and the Acute Disease Quality Initiative (ADQI) Workgroup have outlined consensus definitions, offering standard definitions for phenotyping [4, 10–12]. The severity of AKI, the duration of AKI, and renal recovery after AKI are all critical indicators of overall long-term kidney health. While studies on CKD and AKI phenotypes exist, the authors are unaware of any computable phenotype that identifies and characterizes both CKD and various dimensions of AKI using EHR data and that can be easily customized to different data models and used in real-time (S1–S3 Tables). In addition, there is expanding literature on race-agnostic approaches to address concerns about the lack of biological rationale for including race in these equations for estimating glomerular filtration rate (GFR) and reference creatinine, because there are concerns that race-adjusted estimates for GFR and reference creatinine may lead to underestimation of the incidence of CKD and AKI among African Americans [13–19]. Recently, the National Kidney Foundation endorsed the refit CKD Epidemiology Collaboration (CKD-EPI) equation for estimated GFR (eGFR) without a coefficient for race.

Here, we describe the development and validation of automated race-agnostic algorithms that identify and characterize kidney health in EHR, use data standards, and are usable retrospectively and in real-time. The presented study departs from previous research as follows: a) proposed computable phenotyping algorithm utilizes data standards and a combination of disparate sources of EHR in identifying the stages, duration, and clinical trajectories of both AKI and CKD, providing a detailed description; b) proposed algorithm is race-agnostic. We quantify the effects of race adjustments and compare different approaches commonly used in kidney health assessments, focusing on African American patients.

## Materials and methods

### Data source and participants

Using the University of Florida Health (UFH) Integrated Data Repository as Honest Broker, we created single-center, longitudinal patient cohorts that integrate EHR data.

### Study cohorts and data elements

Three datasets—*DECLARE*, *PICS*, and *AKI EPIC*—were used to develop, verify, and validate phenotyping algorithms, respectively. Studies to develop *DECLARE* and *AKI EPIC* datasets were approved by the University of Florida (UF) Institutional Review Board under a waiver of informed consent and with authorization under the Health Insurance Portability and Accountability Act, while for *PICS* cohort, informed consent was obtained from each subject or their surrogate decision-maker. The *DECLARE*, *AKI EPIC*, and *PICS* studies were approved by the Institutional Review Board of the University of Florida and the University of Florida Privacy Office (IRB #5–2009, IRB 201901123, and IRB 201400611).

Algorithm validation and analyses presented in this study was based on AKI EPIC dataset (S1 Text [Methods], S1 and S2 Figs). We performed all analyses on de-identified datasets. We extracted data from the electronic health records of 156,699 patients ≥18 years admitted to UFH between January 1, 2012, and August 22, 2019 (access date June 1, 2020). After excluding encounters with end-stage kidney disease (ESKD) or with no serum creatinine measurement to determine AKI status during hospitalization, our analysis cohort included 358,580 hospital encounters from 139,152 patients. We utilized data standards including International Classification of Diseases (ICD) and Current Procedural Terminology (CPT) codes for diagnosis and procedures and Logical Observation Identifiers Names and Codes (LOINC) for laboratory variables with corresponding concept identifiers in Observational Health Data Sciences and Informatics (OMOP) common data models. Data elements used for phenotyping are included in S4 and S5 Tables.

### Algorithm development

**Fig 1:**
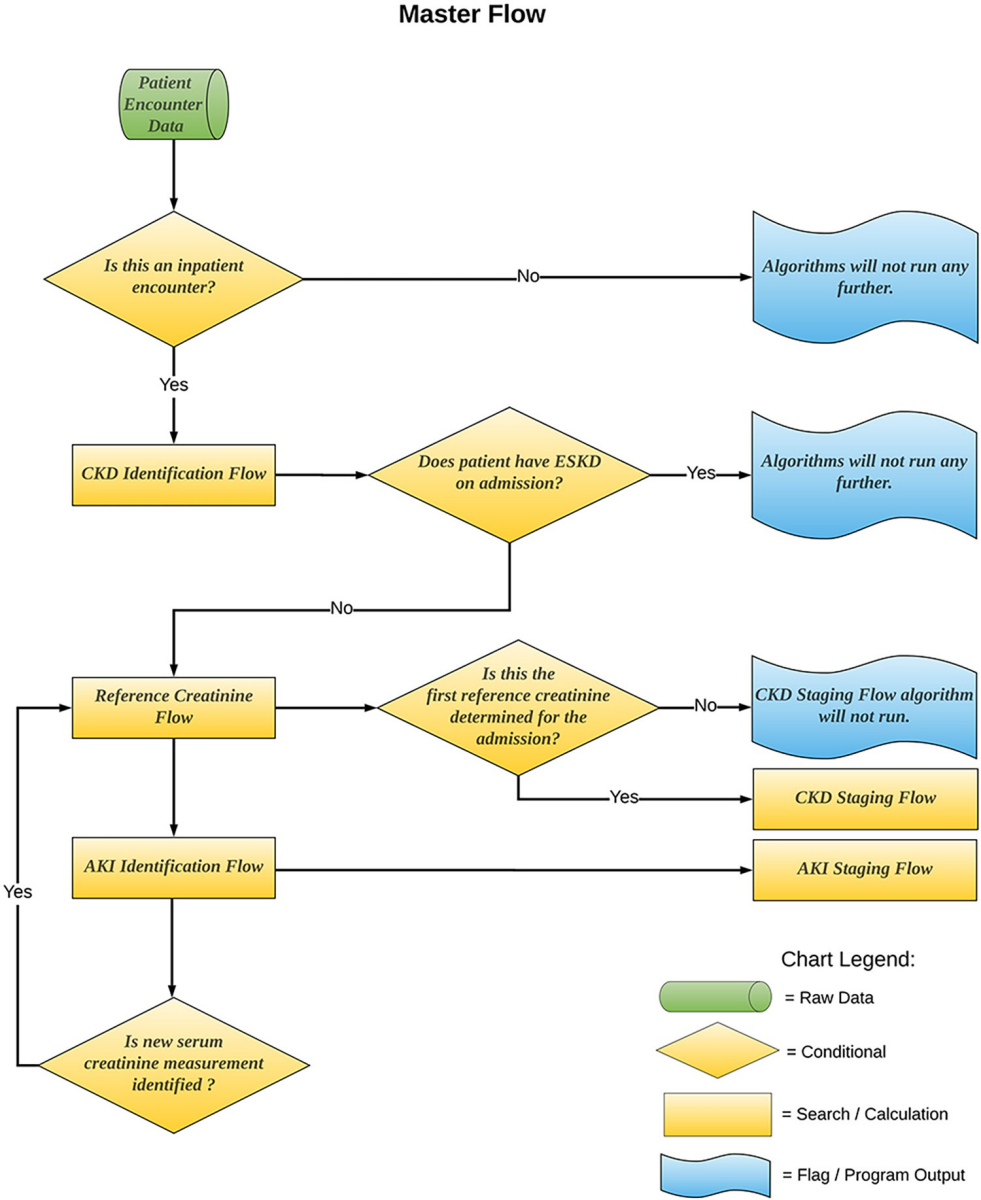
Master flow. Master flow demonstrates incorporation of five rule-based algorithms that can identify and characterize kidney health in any inpatient encounter.

We used KDIGO definitions for AKI and CKD, and we used the ADQI 16 consensus report on renal recovery as the conceptual framework for our *eKidneyHealth* phenotype using a rule-based methodology to replicate, as closely as possible, an experienced clinician’s approach to diagnosing and clinically staging both CKD and AKI and to documenting recovery or persistence of AKI (Fig 1). In order to obtain the eGFR and the estimated creatinine that is part of reference creatinine, two race-agnostic algorithms and one race-adjusted algorithm have been developed. These three algorithms follow similar logic except for the way race was considered for calculation of estimated creatinine and eGFR (Table 1). The race-adjusted algorithm calculated an estimated creatinine by back-calculation from the Modification of Diet in Renal Disease Study (MDRD) equation using equation as in Levey et al. [23] and calculated eGFR using the 2009 CKD-EPI formula, both of which includes race modifier. The first race-agnostic algorithm, referred to as race-agnostic algorithm 1, removed race modifier from the formula used by the race-adjusted algorithm for calculation of estimated creatinine and eGFR. The second race-agnostic algorithm, referred to as race-agnostic algorithm 2, calculated an estimated GFR and creatinine using 2021 CKD-EPI refit without race. The three algorithms analyze a single hospital admission using all data available during and prior to the index admission. The data for the index admission is analyzed temporally from the beginning to the end of the admission, with identification of each new measurement of serum creatinine triggering another cycle of analysis. Results were compared to the clinical adjudication as ground truth.

**Table 1:** Formulas used for estimated creatinine and estimated glomerular filtration rate calculations by the three algorithms.

Output	Algorithm	Formula
Estimated reference creatinine*	Race-adjusted algorithm	MDRD eGFR = 186 × (Scr)^-1.154^× (Age)^-0.203^× (0.742 if female) × (1.21 if African American)
	Race-agnostic algorithm 1	MDRD eGFR = 186 × (Scr)^-1.154^× (Age)^-0.203^× (0.742 if female)
	Race-agnostic algorithm 2	CKD EPI 2021 refit without race eGFR = 142 × min (Scr/κ, 1)^β^ × (Scr/κ, 1)^-1.200^ × 0.9938^Age^ × (1.012 if female)
Estimated glomerular filtration rate	Race-adjusted algorithm	CKD EPI 2009 eGFR = 141 × min (Scr/κ, 1)^α^× (Scr/κ, 1)^-1.209^ × 0.993^Age^ × (1.018 if female) × (1.159 if African American)
	Race-agnostic algorithm 1	CKD EPI 2009 eGFR = 141 × min (Scr/κ, 1)^α^ × (Scr/κ, 1)^-1.209^ × 0.993^Age^ × (1.018 if female)
	Race-agnostic algorithm 2	CKD EPI 2021 refit without race eGFR = 142 × min (Scr/κ, 1)^β^ × (Scr/κ, 1)^-1.200^ × 0.9938^Age^ × (1.012 if female)

*Estimated reference creatinine values are calculated using back-calculation and solution for Scr by setting eGFR to 75.

κ is 0.7 for female patients and 0.9 for male patients.

α is -0.329 for female patients and -0.411 for male patients.

β is -0.241 for female patients and -0.302 for male patients.

### Identification of CKD

Any evidence of preadmission CKD or ESKD was determined by each algorithm first using all available administrative codes in a patient’s medical record to identify patients with CKD, ESKD, and any history of kidney transplantation using a previously validated combination of ICD-9 or ICD-10 codes (S3 Fig and S6–S10 Tables). Patients who had CKD by diagnosis or procedure codes are considered to have CKD by medical history, and others are checked to determine if they had CKD by creatinine criteria. Each algorithm also accounts for any episodes of AKI without renal recovery that occurred within three months of the index admission (S11 Table).

### Determination of reference creatinine

The Reference Creatinine Flow is used to calculate a reference serum creatinine level for the admission, which then is used to calculate the eGFR for CKD staging, AKI identification, and staging (S4 Fig). Initially, the algorithm determines if the creatinine measurement that has triggered this run of the algorithm was obtained within the first seven days of the admission. If the index creatinine measurement is from the first seven days of the admission, we used a list of all serum creatinine levels with time and date stamps to calculate the reference creatinine. If there were previous creatinine measurements in the interval 0–7 days before admission, we used the minimum creatinine level during that interval as reference value 1. If there were previous creatinine measurements in the interval 8–365 days before admission, we used the median creatinine level during that interval as reference value 2 [20–22]. The reference creatinine is then the minimum of reference value 1, reference value 2, and the admission creatinine (S4 Fig). For patients with no history of CKD, the reference creatinine is the minimum of reference value 1, reference value 2, the admission creatinine, and estimated creatinine. Estimated creatinine values are obtained by back-calculation from existing formulas assuming that baseline eGFR is 75 ml/min per 1.73 m^2^.

We compared results using three methods of estimating creatinine to examine the effect of race adjustment for African Americans. The race-adjusted algorithm and the race-agnostic algorithm 1 calculated an estimated creatinine by back-calculation from the Modification of Diet in Renal Disease Study (MDRD) equation with and without race multiplier using equation as in Levey et al. [23], respectively. The race-agnostic algorithm 2 calculated an estimated creatinine by back-calculation from the 2021 CKD-EPI refit without race [17]. For encounters with preadmission CKD but no preadmission or admission creatinine, the first creatinine of the encounter was used as the reference creatinine to determine the first AKI status and stage of the encounter, but the eGFR calculation and the CKD staging was not done. For days with no serum creatinine measurement, the AKI stage was imputed by carrying forward the last available. For example, suppose the index creatinine measurement is from eight or more days after admission; in that case, the algorithm identifies the last available reference creatinine if the patient had AKI the day prior or the minimum creatinine from the previous seven days as the reference creatinine otherwise. The algorithm was run for every creatinine measurement identified in the non-ESKD admission to adjust reference creatinine. S12 and S13 Tables describe our method for determining reference creatinine.

### Determination of CKD stages

The G-stage of CKD is based on the calculated eGFR using the CKD-EPI formula. This formula uses the first reference creatinine calculated (S5 Fig) [13, 23]. The race-adjusted algorithm calculated eGFR using the 2009 CKD-EPI formula, while race-agnostic algorithm 1 used the 2009 CKD-EPI formula with the race modifier removed [15]. The race-agnostic algorithm 2 calculated eGFR using 2021 CKD-EPI refit without race [17].

The A-stage of CKD is determined using urine laboratory measurements within one year prior to admission with LOINC measurements (S6 Fig and S14 Table). We determined A-stage using albumin excretion rate (AER) and urine albumin-to-creatinine ratio (UACR) measurements as A1 if there was at least one measurement of AER or UACR <30 mg/g; as A2 if there was at least one measurement of AER or UACR between 30 and 300 mg/g; or as A3 when A1 and A2 criteria are not met. If there were no AER or UACR values available, we used urine protein-to-creatinine ratio (UPCR) values and multinomial logistic models to determine the A-stage (S1 Text [Methods]). If none of these laboratory measurements were available, we used urine protein (UAP) and specific gravity as inputs for multinomial logistic models. We also calculated the distribution of A-stages using the formula by Sumida et al. [24].

### Identification and staging of AKI and renal recovery

The AKI Identification Flow is triggered to run by every new measurement of serum creatinine during admission to determine if the patient has current AKI by KDIGO serum creatinine criteria or by the requirement for kidney replacement therapy (KRT) (S7 Fig). The AKI trajectory Identification Flow identifies the trajectory of AKI according to the duration of AKI and the presence or absence of renal recovery (S8 Fig) [4, 12]. We defined an episode of AKI as beginning when AKI is identified. In conjunction, we defined an episode as ending when there are two consecutive days without AKI. An episode of AKI that resolves completely within 48 hours is termed “rapid reversal,” an episode of AKI persisting beyond 48 hours is termed “persistent” AKI, and an episode of AKI with renal dysfunction persisting beyond 7 days is termed “Acute Kidney Disease” (AKD) [4, 25].

The KDIGO AKI stage was determined for all patients identified by each algorithm (S9 Fig). For a patient undergoing KRT, the AKI stage is “Stage 3 with KRT.” If not, the current reference creatinine was used to stage the AKI by KDIGO serum creatinine criteria. The KRT was determined daily, according to Current Procedural Terminology (CPT) codes and EHR orders for hemodialysis, peritoneal dialysis, and continuous KRTs (S5 and S9 Tables). To determine the impact of the race modifier on AKI status and stages, we quantified changes in classifying AKI status and stage after including the race modifier in the MDRD formula that is part of the reference creatinine for non-CKD patients.

### Phenotype algorithm clinical validation

Three physicians and a medical student trained in the clinical consensus definitions of AKI and CKD independently reviewed the validation cohort of patients to determine if the patients had CKD at the time of admission and/or AKI that developed during the hospitalization. The review sample for the *eKidneyHealth* phenotype algorithm clinical validation was created by selecting inpatient encounters admitted between January 2012 and April 2016 from the AKI EPIC database based on CKD status while stratifying each group into three groups by AKI status and renal recovery. The review sample included 300 selected inpatient encounters, half with CKD and half with no CKD, while stratifying each group into three groups by AKI status and renal recovery (no AKI, AKI with renal recovery, and AKI without renal recovery). We selected a proportional number of patients in each subgroup for review. Half of the patients in each subgroup were selected among the relevant group in the cohort with the highest reference creatinine values, and the other half of the patients were selected among the ones with the lowest reference creatinine values. Differences in ascertainment were resolved by discussion among all four reviewers. We calculated sensitivity, specificity, positive and negative predictive values, and overall accuracy with exact binomial confidence intervals for the computational phenotype relative to clinical adjudication as ground truth by adjusting for prevalence in the cohort [26]. Statistical analyses were performed with SAS (version 9.4; SAS Institute, Inc, Cary, NC), Python (version 3.7), and R software (version 3.5.1).

## Results

### Clinical characteristics

Our final analysis cohort included 358,580 hospital encounters from 139,152 patients, of whom 52% were female and 17% were African American, with an average age of 54 (S15 Table).

### CKD computational phenotypes

Among 358,580 hospital admissions with adequate data for CKD phenotyping (in other words, admissions who had history with ICD codes), the prevalence of CKD was 23%–24%, with 19% of these determinations made by medical history alone and an additional 3%–4% made by creatinine criteria using race-agnostic algorithms (Table 2 and S16 Table). By G-staging, almost half of all CKD patients had normal (G1, 20%–24%) or mildly reduced (G2, 34%–35%) kidney function. Approximately 40% had moderately reduced function (G3a, 20%–21%; G3b, 13%–15%), and the remaining 8% had severely reduced function (G4, 6%–7%; G5, 1%). Among CKD patients, more than half (52%) had reference creatinine determined using values prior to admission, especially using median 8–365 days prior to admission (S12 Table). Among non-CKD patients, the proportion of patients with reference creatinine determined using past creatinine (32%–33%), admission creatinine (49%–51%), and estimated creatinine (16%–20%) were similar across the three algorithms (S12 Table). CKD was more common among African American patients (30%–31% vs 21%–22%), most frequently determined by medical history (S13 Table). Results were similar for the two race-agnostic algorithms, with a slight reduction in number of patients with CKD and patients in higher stages of CKD, due to changes in calculations for the subset of African American cohort. Among 86,379 African American patient admissions, 26,908 (31%) and 26,003 (30%) had CKD based on race-agnostic algorithm 1 and 2, respectively.

**Table 2:** Summary of CKD and AKI characteristics.

	Using race-adjusted algorithm, n (%)	Using race-agnostic algorithm 1, n (%)	Using race-agnostic algorithm 2, n (%)
**Overall number of encounters**	**N = 358,580**	**N = 358,580**	**N = 358,580**
**Preadmission CKD, n (%)**			
Insufficient Data (No CKD with warning)	26 (<0.1)	26 (<0.1)	26 (<0.1)
No CKD	274,338 (77)	272,225 (75)	275,819 (77)
CKD	84,216 (23)	86,329 (24)	82,735 (23)
**No AKI during hospitalization, n (%)**	304,749 (85)	304,174 (85)	304,909 (85)
**AKI during hospitalization, n (%)**	53,831 (15)	54,406 (15)	53,671 (15)
Reference serum creatinine, median (25^th^, 75^th^)	0.85 (0.68, 1.11)	0.84 (0.68, 1.09)	0.89 (0.69, 1.16)
Reference serum creatinine, mean (SD)	1.03 (0.8)	1.03 (0.8)	1.07 (0.8)
**Maximum AKI Stage, n (%)**	53,831	54,406	53,671
Stage 1	36,062 (67)	36,396 (66)	36,001 (67)
Stage 2	9,403 (17)	9,588 (18)	9,316 (17)
Stage 3 (with or without KRT)	8,366 (16)	8422 (15)	8,354 (16)
KRT, n (%)	2,058 (4)	2,058 (4)	2,058 (4)
**AKI trajectories, n (%)**			
Rapidly reversed AKI	31,291 (58)	31,605 (58)	31,784 (59)
Persistent AKI	22,540 (42)	22,801 (42)	21,887 (41)

Abbreviations: AKI, acute kidney injury; CKD, chronic kidney disease, KRT, kidney replacement therapy.

Race-adjusted algorithm calculated eGFR using 2009 CKD-EPI formula, while race-agnostic algorithm 1 used 2009 CKD-EPI formula with race modifier removed. Race-agnostic algorithm 2 calculated eGFR using the 2021 CKD-EPI refit without race.

### AKI computational phenotypes

Among 358,580 hospital admissions with creatinine data required for AKI phenotyping, the incidence of AKI was 15% (Table 2 and S17 Table). The maximum AKI stage was predominantly stage 1 (66%–67%), with AKI stage 2 identified in 17%–18%, and AKI stage 3 in 15%–16%. About 4% of all hospital admissions included KRT. Twelve percent of patients developed more than one episode of AKI. The median duration of AKI was two days (interquartile range 1–4 days), and 41%–42% of all AKI episodes persisted for more than 48 hours. The median duration of KRT was 10 days (interquartile range 5–20 days). AKI characteristics were similar for race-agnostic and race-adjusted algorithms in all cohorts.

### Phenotyping algorithm performance relative to clinical adjudication

Performance of the CKD and AKI phenotyping algorithms relative to clinical adjudication was evaluated using 300 cases (Table 3 and S18 and S19 Tables). When race-adjusted algorithm 2 was used, the CKD phenotyping algorithm yielded a positive predictive value of 72% (95% confidence interval [CI] 62%–79%), negative predictive value of 100%, sensitivity of 100% (95% CI 97%–100%), and specificity of 88% (95% CI 82%–93%). The AKI phenotyping algorithm yielded a positive predictive value of 89% (95% CI 68%–97%), negative predictive value of 100% (95% CI 99%–100%), sensitivity 99% (95% CI 97%–100%), and specificity 98% (95% CI 93%–100%). Reasons for mismatches between phenotyping algorithm and manual chart review include wrong ICD code assignments, erroneous laboratory measurement, and insufficient creatinine history with details provided in footnote of Table 3. S18 and S19 Tables show diagnostic performance for race-adjusted and race-agnostic 1 algorithms, which were similar in this sample dataset.

**Table 3:** Comparison of performance of chronic kidney disease (CKD) and acute kidney injury (AKI) phenotyping algorithms, using race-agnostic algorithm 2, to manual chart review in diagnosing CKD and AKI.

	Manual chart review for CKD			Manual chart review for AKI		
*eKidneyHealth* Phenotyping Algorithm	Case	Control	Total	Case	Control	Total
Case, n	132	20^a^	152	202	2^b^	204
Control, n	0	148	148	2^c^	94	96
Total, n	132	168	300	204	96	300
Positive predictive value (95% Confidence Interval)			72% (62%, 79%)			89% (68%, 97%)
Negative predictive value (95% Confidence Interval)			100% (NA, NA)			100% (99%, 100%)
Sensitivity (95% Confidence Interval)			100% (97%, 100%)			99% (97%, 100%)
Specificity (95% Confidence Interval)			88% (82%, 93%)			98% (93%, 100%)
Accuracy (95% Confidence Interval)			91% (87%, 94%)			98% (96%, 99%)

Reasons for mismatches between phenotyping algorithm and manual chart review includes

^a^ Assignment of wrong ICD code for patient who had AKI (n = 4), assignment of wrong ICD code (n = 2), assignment of wrong ICD code for nephrotic syndrome (n = 4), non-specific CKD code for patient who had AKI (n = 9), and CKD captured based on creatinine criteria by algorithm (n = 1)

^b^ Reference creatinine wrong based on erroneous laboratory measurement (n = 2)

^c^ Wrong reference creatinine due to insufficient creatinine history for CKD patient (n = 1) and wrong reference creatinine due to wrong CKD code assignment (n = 1)

Race-agnostic algorithm 2 calculated eGFR using the 2021 CKD-EPI refit without race.

### Race-agnostic CKD and AKI phenotyping algorithm results compared with results from race-adjusted algorithms for African American cohort

The reference creatinine values used for determining CKD staging as well as AKI status and stage were affected when using MDRD methods, which yielded higher reference creatinine for all African American patients. The MDRD method was used to determine reference creatinine in 13.4% of all African American patients using race-agnostic phenotyping and 6.4% of all African American patients using race-adjusted phenotyping (S20 Table).

Among 86,379 African American patient admissions, 26,908 (31%) and 26,003 (30%) had CKD based on race-agnostic algorithm 1 and 2, respectively. When the race-adjusted algorithm was used for the 86,379 African American patients instead of race-agnostic algorithm 1 and 2, respectively, the median increases in eGFR were 15.31 ml/min/1.73m^2^ (25th–75th 12.4–18.0) and 11.3 (8.1, 15.4); when the race-adjusted algorithm was used for the subset of patients with CKD, the median increases in eGFR were 9.9 ml/min/1.73m^2^ (25th–75th 7.1–13.8) and 6.3 (4.2, 9.6) (S21 Table). When compared to race-agnostic algorithm 1, race adjustment reclassified 2,113 (8%) CKD encounters to no CKD, and 7,901 (29%) to a less severe CKD stage (S12, S22 and S23 Tables). Compared to race-agnostic algorithm 1, race adjustment also reclassified the G-staging for the following percentages of patients: 33% of G2 patients were reclassified to G1, 56% of G3A to G2, 44% of G3B to G3A, 36% of G4 to G3B, and 35% of G5 to G4. On the other hand, compared to race-agnostic algorithm 2, the effect of race-adjustment reclassification was slightly less: race adjustment reclassified 1,208 (5%) CKD encounters to no CKD and 4,606 (18%) to a less severe CKD stage (Table 4). Compared to race-agnostic algorithm 2, race adjustment also reclassified the G-staging for the following percentages of patients: 20% of G2 patients were reclassified to G1, 35% of G3A to G2, 27% of G3B to G3A, 21% of G4 to G3B, and 20% of G5 to G4.

**Table 4:** Reclassification of CKD status and CKD G-stages, using race agnostic algorithm 2, among African American patients after race-adjustment.

	CKD G-stage using race-adjusted algorithm									
	No CKD (n = 61,579, 71%)	CKD (n = 24,795, 29%)	G1 (n = 8,141, 33%)	G2 (n = 7,986, 32%)	G3a (n = 3,934, 16%)	G3b (n = 2,860, 11%)	G4 (n = 1,420, 6%)	G5 (n = 237, 1%)	No staging (n = 217, 1%)
**CKD G-stage using race-agnostic algorithm 2**	No CKD (n = 60,371, 69%)	60,371 (100)	0 (0)	0 (0)	0 (0)	0 (0)	0 (0)	0 (0)	0 (0)	0 (0)
	CKD (n = 26,003, 30%)	1,208 (5)	24,795 (95)	8,141 (33)	7,986 (32)	3,934 (16)	2,860 (12)	1,420 (6)	237 (1)	217 (1)
	G1 (n = 6,791, 26%)	299 (4)	6,492 (96)	6,492 (100)	0 (0)	0 (0)	0 (0)	0 (0)	0 (0)	0 (0)
	G2 (n = 8,802, 33%)	779 (9)	8,023 (91)	1,649 (20)	6,374 (80)	0 (0)	0 (0)	0 (0)	0 (0)	0 (0)
	G3a (n = 4,752, 18%)	118 (2)	4,634 (97)	0 (0)	1,612 (35)	3,022 (65)	0 (0)	0 (0)	0 (0)	0 (0)
	G3b (n = 3,405, 13%)	6 (0.2)	3,399 (99)	0 (0)	0 (0)	912 (27)	2,487 (73)	0 (0)	0 (0)	0 (0)
	G4 (n = 1,733, 6%)	0 (0)	1,733 (100)	0 (0)	0 (0)	0 (0)	373 (21)	1,360 (79)	0 (0)	0 (0)
	G5 (n = 297, 1%)	0 (0)	297 (100)	0 (0)	0 (0)	0 (0)	0 (0)	60 (20)	237 (80)	0 (0)
	No staging (n = 223, 1%)	6 (3)	217 (97)	0 (0)	0 (0)	0 (0)	0 (0)	0 (0)	0 (0)	217 (100)

Abbreviations: CKD, chronic kidney disease.

Gray shading indicates patients who were reclassified into no CKD or less severe stages of CKD after race adjustment.

Race-agnostic algorithm 2 calculated eGFR using the 2021 CKD-EPI refit without race.

Within the 86,379 African American patient admissions, a subset of 63,090 had CKD status identified by laboratory values rather than medical history. From that subset, 3,624 (6%) admissions were classified by race-agnostic algorithm 1 as having CKD; when race adjustment was used, 2,113 (58%) were reclassified to no CKD and 551 (15%) were reclassified to less severe CKD stage (S24 Table). Similar changes were observed for race-agnostic algorithm 2. Of the 12,451 (14.4%) encounters with AKI based on race-agnostic algorithm 1, the race adjustment reclassified 591 (5%) to no AKI, decreasing the prevalence of AKI from 12,451 (14.4%) to 11,876 (13.7%), and reclassified 305 (2%) to a less severe AKI stage (S25 and S26 Tables). Percentages of AKI patients reclassified from Stage 2 to Stage 1, and from Stage 3 to Stage 2, were 12% and 3%, respectively. Similarly, of the 12,251 (14.2%) encounters with AKI based on race-agnostic algorithm 2, the race adjustment reclassified 382 (3%) to no AKI and 196 (2%) to a less severe AKI stage (Table 5). Percentages of AKI patients reclassified from Stage 2 to Stage 1, and from Stage 3 to Stage 2, were 8% and 2%, respectively.

**Table 5:** Reclassification of AKI status and AKI stages, using race agnostic algorithm 2, after race adjustment among African American patients.

			AKI stage with race adjustment			
		No AKI (n = 74,503, 86%)	AKI (n = 11,876, 14%)	Stage 1 (n = 7,937, 67%)	Stage 2 (n = 1,891, 16%)	Stage 3 (n = 2,048, 17%)
**AKI stage using race-agnostic algorithm 2**	No AKI (n = 74,128, 86%)	74,121 (100)	7 (0.1)	7 (100)	0 (0)	0 (0)
	AKI (n = 12,251, 14%)	382 (3)	11,869 (97)	7,930 (67)	1,891 (16)	2,048 (17)
	Stage 1 (n = 8,159, 66%)	382 (5)	7,777 (95)	7,774 (100)	3 (0.1)	0 (0)
	Stage 2 (n = 2,006, 16%)	0 (0)	2,006 (100)	156 (8)	1,848 (92)	2 (0.01)
	Stage 3 (n = 2,086, 17%)	0 (0)	2,086 (100)	0 (0)	40 (2)	2,046 (98)

Abbreviations. AKI, acute kidney injury.

Gray shading indicates patients who were reclassified into no AKI or less severe stages of AKI patients after race adjustment.

Reference creatinine used in determination of AKI stages involves calculation of an estimated creatinine for no CKD patients. Race-agnostic algorithm 2 calculates estimated creatinine by back calculation from the 2021 CKD-EPI refit without race.

## Discussion

Originally developed by genomics researchers to query EHRs and identify patients with rare genetic diseases, computable phenotyping is gaining popularity in both clinical and health services research applications [27–29]. An automated approach to identifying and characterizing kidney disease by combining the global perspective offered by administrative codes, with the clinical detail provided in EHR data, could provide accurate and reliable inferences about the presence and severity of clinical illness [30]. Computable phenotypes using established data standards and a common data model provide the opportunity to get fast and consistent annotation of multiple acute illnesses across multiple centers and further advance Artificial Intelligence/Machine Learning (AI/ML) applications to a broader system adhering to the FAIR principles (Findable, Accessible, Interoperable, Reproducible). Automated and accurate identification and staging of CKD and AKI using electronic data has the potential to facilitate early recognition and appropriate management with targeted preventative and therapeutic interventions, impacting the substantial mortality, morbidity, and health care expenditures associated with kidney disease [31, 32]. As a result of using electronic data, we will develop predictive approaches, optimize AKI alerts, standardize and improve the quality of care provided in the setting of AKI, and track patients/events across populations and care platforms [33]. The authors are unaware of any computable phenotype that identifies and characterizes both CKD and various dimensions of AKI and CKD while using EHR data that can be easily customized to different data models and used in real-time. We utilized data standards including ICD and CPT codes for diagnosis and procedures and LOINC codes for laboratory variables with corresponding concept identifiers in OMOP common data models. We developed and validated *eKidneyHealth*, a computable phenotype for kidney health encompassing both AKI and CKD, while maintaining consistency with KDIGO and ADQI guidelines and addressing the potential racial biases introduced by race adjustments in GFR and creatinine calculations. We evaluated computational phenotyping relative to clinical adjudication, demonstrating that the algorithm outperforms existing tools and administrative codes across the broad spectrum of disease severity, including minor stages of AKI [34, 35].

Prior work has demonstrated that severe AKI (e.g., the Major Adverse Kidney Events by 30 days [MAKE30] composite of death, new KRT, or persistent renal dysfunction) can be identified using EHR data with high sensitivity and specificity [36]. Yet, methods for identifying mild and moderate AKI using EHR data are lacking [33]. Mild to moderate AKI is much more common than severe AKI and is associated with poor clinical outcomes and increased resource use [5, 37]. Beyond the aforementioned potential for improving clinical care, the ability to more accurately identify all stages of AKI as well as CKD could improve the quality of research endeavors that require accurate and precise measurement of kidney disease–associated mortality, morbidity, health care expenditures, quality metrics, and provider clinical performance [38].

Contributors of racial disparity in AKI and CKD rates were investigated from several perspectives in prior studies. Eneanya et al. [16] discussed the role of race and structural racism and the effect of inequities in major social determinants of health on kidney health and reported close links between race and ethnicity to residential segregation [39], educational and income inequalities, reduced access to health-care resources, and elevated exposure to environmental toxins [40]. In Grams et al. [41], the authors related the higher risk of AKI among African American people to inferior socioeconomic factors such as lower income and education level. Based on disparities in health and healthcare delivery in African American communities, there have been recent studies that questioned the biological rationale for including race and evaluated the potential clinical implications of removing race term in GFR equations as that might influence timely access to care and kidney transplantation. When the CKD-EPI eGFR race modifier is applied for African American patients, the percentage of patients classified as CKD and more severe stages of CKD was decreased. Nearly one in four African Americans would be upstaged from CKD stage 3B to 4 when the race adjustment is removed, as also shown by Ahmed et al. [13]. These findings are consistent with recently reported studies that evaluated the potential clinical implications of removing race adjustments from the CKD-EPI formula for eGFR [13, 15, 18]. Estimated GFR values based on 2021 CKD-EPI and 2009 CKD-EPI equations were compared with measured GFR values in a recent study by Meeusen et al. [42] According to their findings, 2021 CKD-EPI equation underestimates measured GFR more than race-adjusted 2009 CKD-EPI equation, which supports reclassification outcomes presented in our study [42].

Removing the race modifier from equations that estimate kidney function could begin to reverse inequities in managing kidney disease for African American patients. Eneanya et al. showed potential beneficial implications of the removal of race from the 2009 CKD-EPI equation in their Table 1 on CKD diagnosis, referral to nephrologist, eligibility for kidney transplant waiting list, health insurance coverage for kidney disease education, and impact on patient-centered outcomes and health equity [16]. Correct identification of CKD stage may enable appropriate CKD management including nephrology referral, radiographic diagnostic assessment, initiation of dialysis, transplant referral, patient education regarding treatment options, and kidney donor candidate evaluation decisions. Potential undesired consequences of new CKD diagnoses and classification to more advanced stages of CKD may include possible changes in eligibility of a patient for being a living kidney donor or continuum of aggressive treatment regimens even if there could be room for dose reduction. Another implication is reduced access to certain diagnosis techniques due to contraindication issues of drugs used for imaging in CKD patients with advanced stages [15, 16, 43]. Recent reports by the Task Force, established by the National Kidney Foundation (NKF) and American Society of Nephrology (ASN) to reassess the inclusion of race in the estimation of GFR, evaluated 26 approaches for the estimation of eGFR. Delgado et al. [44] summarizes possible consequences of various approaches for clinical decision-making in medical and nephrology care, including race agnostic algorithm 1 (referred to as CKD-EPIcr_NB) and race agnostic algorithm 2 (referred to as CKD-EPIcr_R) compared to race-adjusted algorithm in Tables 4 and 5 emphasizing effects on kidney donor candidate evaluation decisions, CKD screening or detection, and risk prediction. The Task Force recommended immediate implementation of the CKD-EPI creatinine equation refit without the race variable in all laboratories due to inclusion of diversity in the refit’s development, acceptable performance characteristics, and potential adverse consequences not disproportionately affecting any one group while facilitating increased, routine, and timely use of cystatin C [44].

This study has several important limitations. Because of relying partially on administrative codes for AKI and CKD, our computational phenotype is partially dependent on accurate EHR disease coding, which is rarely achieved. Since identifying both AKI and CKD depend on changes in serum creatinine and baseline creatinine, the phenotype misses other important clinical signs of kidney injury and illness, such as oliguria, which is an early sign of kidney injury that is not captured by our algorithm due to a lack of reliable data. Likewise, changes in urine and serum biomarkers are not captured by our phenotype. Use of a wealth of structured and unstructured data for phenotyping and deep phenotyping methods will be considered for future research. Finally, while our phenotyping algorithms capture administrative codes for a wide variety of kidney diseases, they do not capture specific etiologies of kidney disease. We used single-institution data, limiting the generalizability of our findings.

## Conclusion

There is crucial need for early detection of AKI and information about reference creatinine, CKD status, AKI status, and stage of the patient in the EHR for a comprehensive, time-efficient, and consistent evaluation of kidney health status and to help healthcare providers save time in the evaluation process. Including race adjustments may underestimate the incidence and severity of AKI and CKD among African Americans. Removing the race modifier from equations that estimate kidney function could begin to reverse inequities in managing kidney disease for African American patients. We developed and validated the *eKidneyHealth* algorithms, race-agnostic computable phenotypes that identify and characterize kidney health in hospitalized adults, use data standards, and can be run on OMOP common data models. Currently, these algorithms are intended to provide healthcare providers with detailed kidney health assessment and can be utilized as part of clinical decision-support systems in future studies. Automated identification and staging of AKI and CKD using electronic data has the potential to assist healthcare providers with clinical decision-making and facilitate early recognition and appropriate management with targeted preventative and therapeutic interventions, impacting the substantial mortality, morbidity, and health care expenditures associated with kidney disease.

## Supporting information

S1 Text Supplementary methods. Detailed description of data elements and methods.
(DOCX)

S1 Table Summary of studies on chronic kidney disease (CKD) phenotyping. (DOCX)

S2 Table Performance of studies on CKD phenotyping. (DOCX)

S3 Table Summary of studies on AKI alerts. (DOCX)

S4 Table Data elements that are used to run CKD phenotyping algorithm. (DOCX)

S5 Table Data elements that are used to run AKI phenotyping algorithm. (DOCX)

S6 Table Administrative codes used for end stage kidney disease. (DOCX)

S7 Table Administrative codes used for chronic kidney disease. (DOCX)

S8 Table Administrative codes for kidney transplant. (DOCX)

S9 Table Administrative codes used for renal-replacement therapy. (DOCX)

S10 Table ICD codes used for history of acute kidney injury (AKI). (DOCX)

S11 Table Details on output categories for CKD algorithm. (DOCX)

S12 Table Methods used to define reference creatinine in validation cohort using race-agnostic and race-adjusted algorithms. (DOCX)

S13 Table Methods used to define reference creatinine in validation cohort using race-agnostic phenotyping algorithms stratified by African American race. (DOCX)

S14 Table Logical Observation Identifier Names and Codes (LOINC) codes used for CKD A-staging. (DOCX)

S15 Table Clinical characteristic for patients without ESKD for each cohort. (DOCX)

S16 Table CKD characteristics using race-adjusted and race-agnostic algorithms. (DOCX)

S17 Table AKI characteristics using race-adjusted and race-agnostic algorithms. (DOCX)

S18 Table Comparison of performance of chronic kidney disease (CKD) and acute kidney injury (AKI) phenotyping algorithms, using race-adjusted algorithm, to manual chart review in diagnosing CKD and AKI. (DOCX)

S19 Table Comparison of performance of chronic kidney disease (CKD) and acute kidney injury (AKI) phenotyping algorithms, using race agnostic algorithm 1, to manual chart review in diagnosing CKD and AKI. (DOCX)

S20 Table Comparison of reference creatinine determination methods used for race-agnostic algorithms to race-adjusted phenotyping algorithms among African American patients. (DOCX)

S21 Table CKD status and G-stages for African American encounters using race-adjusted and race-agnostic algorithms. (DOCX)

S22 Table Reclassification of CKD status and CKD stages, using race agnostic algorithm 1, among African American patients after race-adjustment. (DOCX)

S23 Table Reclassification of CKD stages among African-American CKD patients identified by medical history using into less severe CKD stages after race adjustment. (DOCX)

S24 Table Reclassification of CKD status and CKD stages after race adjustment among African American patients who do not have CKD by medical history. (DOCX)

S25 Table Reclassification of AKI status and AKI stages, using race agnostic algorithm 1, after race adjustment among African American patients. (DOCX)

S26 Table AKI status and stages for African American encounters using algorithms without and with race adjustment. (DOCX)

S1 Fig Clinical datasets. (TIF)

S2 Fig Cohort selection and exclusion criteria. (TIF)

S3 Fig CKD identification flow. This flow shows the rules for determination of preadmission chronic kidney disease using data from the index admission along with historical data prior to that admission.
(TIF)

S4 Fig Determination of reference creatinine flow. This flow shows the rule for determination of reference creatinine that changes dynamically during the index admission. *Race-adjusted algorithm and race-agnostic algorithm calculate estimated creatinine by back-calculation from the Modification of Diet in Renal Disease Study equation with and without race multiplier, respectively. Race-agnostic algorithm 2 calculates estimated creatinine by back calculation from the 2021 CKD-EPI refit without race.
(TIF)

S5 Fig CKD G-staging flow. This flow shows rule for determination of G-stages for patients with chronic kidney disease.
(TIF)

S6 Fig CKD A-staging flow. This flow shows rule for determination of A-stages for patients with chronic kidney disease.
(TIF)

S7 Fig AKI identification flow. This flow shows rule for determination of type of kidney injury/disease during the index admission.
(TIF)

S8 Fig AKI trajectory identification flow. This flow shows rule for determination of type of kidney injury/disease during the index admission.
(TIFF)

S9 Fig AKI staging flow. This flow shows rule for determination of AKI stages for patients with acute kidney injury using KDIGO criteria.
(TIF)
