# Supplementary material for: Development and validation of a race-agnostic computable phenotype for kidney health in adult hospitalized patients
Source: PLoS One. 2024 Apr 23;19(4):e0299332. doi: 10.1371/journal.pone.0299332 (PMC11037544; doi:10.1371/journal.pone.0299332)
Supplement: S1 Text — Detailed description of data elements and methods. (DOCX) [file pone.0299332.s001.docx]

Supplementary Material

S1 Text. Supplementary Methods

**Data Source and Participants**

Using the University of Florida Health (UFH) Integrated Data Repository as Honest Broker, we have created single-center longitudinal patient cohorts that integrate data from a comprehensive inpatient and outpatient electronic health records (EHR) (Epic Systems, Verona, Wisconsin, USA) with laboratory, pharmacy, billing, and other administrative data [1].

**Study Cohorts and Data Elements**

Three datasets were used to develop, verify, and validate phenotyping algorithms (S1 Figure). Studies to develop *DECLARE* and *AKI EPIC* datasets were approved by the University of Florida (UF) Institutional Review Board under a waiver of informed consent and with authorization under the Health Insurance Portability and Accountability Act, while for the *PICS* cohort, informed consent was obtained from each subject or their surrogate decision-maker. The *DECLARE, AKI EPIC*, and *PICS* studies were approved by the Institutional Review Board of the University of Florida and the University of Florida Privacy Office (IRB #5–2009, IRB 201901123, and IRB 201400611).

The Algorithm Development Cohort used the *DECLARE* dataset of 51,457 adult patients who underwent surgery at UFH between January 2000 and November 2010 (S1 Figure) [2-5]. The Algorithm Verification Cohort used the *PICS* cohort that combines EHR and research data for 245 sepsis adult patients prospectively enrolled in a longitudinal cohort study at UF between January 2015 and July 2017 [6, 7]. The Algorithm Validation Cohort used the *AKI EPIC* dataset of 156,699 adult patients admitted to UFH between January 1, 2012, and August 22, 2019, to present results and a subset a cohort of 300 subjects for validation. After exclusion of encounters with end-stage renal disease (ESRD) or with no serum creatinine measurement to determine acute kidney injury (AKI) status during hospitalization, our analysis cohort included 358,580 hospital encounters from 139,152 patients (S1–S2 Figures).

With the exception of the *PICS* cohort, the datasets represented routinely collected clinical and administrative data at the study institution. Every admission to the hospital corresponded to a unique inpatient encounter. Each dataset included all diagnosis and procedure codes, structured and unstructured clinical data, demographic information, vital signs, laboratory values, and medication data for all index inpatient encounters. Data elements used for phenotyping are included in S4 Table and S5 Table. Laboratory data was available for 12 months prior to the index admission and for 12 months after. For each encounter we collected all current and prior International Classification of Diseases, 9^th^ and 10^th^ revision, clinical modification (ICD-9-CM and ICD-10-CM, respectively) and Current Procedural Terminology (CPT) codes with the IDR and identified the appropriate codes for both AKI and CKD (S6–S10 Tables).

Development of the Algorithm

We used the Kidney Disease: Improving Global Outcomes (KDIGO) Clinical Practice Guideline definitions for AKI and CKD and a consensus report of the Acute Disease Quality Initiative (ADQI) 16 Workgroup on renal recovery, as the conceptual framework for our comprehensive assessment of kidney health in adults during an inpatient hospitalization, *eKidneyHealth* [8-11].

The approach utilized EHR data elements including diagnostic and procedure codes, laboratory results, and dialysis intake and output results. We developed the *eKidneyHealth* algorithms using a rule-based methodology to replicate, as closely as possible, an experienced clinician’s approach to diagnosing and clinically staging both CKD and AKI and to documenting recovery or persistence of AKI if it occurs. The final algorithm incorporates five rule-based algorithms that can identify and characterize kidney health in any inpatient encounter and, depending upon implementation, can be used either in real-time or in retrospective analysis (Figure 1).

The *eKidneyHealth* phenotype is constructed from three algorithms to identify CKD along with three algorithms to identify AKI, using a “Reference Creatinine” algorithm that identifies every patient’s baseline creatinine and which is updated every time a new serum creatinine value is identified. These three algorithms follow similar logic except for the way race was considered for calculation of estimated creatinine and estimated glomerular filtration rate. The outputs from the overall algorithm include CKD by medical history and/or creatinine criteria including ESKD, KDIGO G-stage of any CKD, new AKI, persistent AKI, AKD, recovery from AKI, persistent AKI or AKD, and KDIGO stage of any AKI.

The algorithms are designed to analyze a single hospital admission for a patient using all appropriate data available during and prior to the index admission. Data for the index admission is analyzed temporally from beginning to end of the admission, with identification of each new measurement of serum creatinine triggering another cycle of analysis. Central to the algorithm is the determination of a reference creatinine: an estimate of a patient’s creatinine in the steady state, before any AKI occurs, and/or after the patient recovers from any pre-existing AKI. The algorithm first determines if CKD is present using data from the index admission along with historical data prior to that admission (S3 Figure). The first creatinine value identified during the index admission then triggers the assessment of the first reference creatinine (S4 Figure), then the staging of CKD if present (S6–S6 Figures), then the identification of any AKI (including persistent AKI, AKD, and any recovery from kidney injury) (S7-S8 Figure), and finally the staging of AKI if present (S8 Figure). Every subsequently identified creatinine value triggers a reassessment of the reference creatinine and identification and restaging of AKI. Any AKI that develops during an inpatient encounter is thus captured, as well as any recovery from AKI, and the reference creatinine is adjusted each time as appropriate.

The algorithm was written in Python 3.7 software. Data elements were extracted from the dataset, transformed and cleaned as needed, and then organized into Python data structures for fast query of a patients’ history. Each patient record in the algorithm verification *PICS* dataset of 245 sepsis patients contains clinical adjudication for CKD, AKI, and renal recovery status performed by two independent nephrologists as a part of a previous study protocol [7]. Development and editing of the code were done using this database. Code subroutines were revised in an iterative manner by running the subroutines on the development and then verification cohorts, with the results compared to previous clinical adjudication.

1. Identification of CKD

The CKD Identification Flow is used to determine if the patient has any evidence of CKD or ESKD, and to distinguish between patients with pre-existing CKD and those with new onset AKI (S3 Figure). The algorithm first uses all available administrative codes in patients’ medical records to identify patients with CKD, ESKD, and any history of kidney transplantation using previously validated combination of ICD-9 or ICD-10 codes (S6–10 Tables) [12]. A patient is considered to have ESKD if the patient has a) any of the ICD codes for end-stage kidney disease (S6 Table), b) CKD codes (ICD-9 Diagnosis Code of 585.5 or ICD-10 Diagnosis Code of N18.5) and has any of the kidney replacement therapy (KRT) codes (S9 Table), or c) any of the CKD Codes (S7 Table) and any of the KRT Codes (S9 Table) but does not have any of the AKI Codes (S10 Table). Patients with previous renal transplant are considered to have CKD after kidney transplant by medical history. Patients who had CKD by diagnosis and/or procedure codes are considered to have CKD by medical history, and patients who did not have CKD by medical history are checked to determine if they had CKD by creatinine criteria; that is; if they had at least two serum creatinine measurements separated at least three months apart with corresponding estimated glomerular filtration rate (eGFR) < 60 ml/min/1.72m^2^ [13]. A subset of patients with no available previous medical history in the UF EHR were marked as having insufficient data to determine CKD status. The algorithm also accounts for any episodes of AKI without renal recovery that occurred within three months of the index admission. Outputs and definitions from the algorithm are listed in S11 Table.

1. Determination of reference creatinine

The Reference Creatinine Flow is used to calculate a reference serum creatinine level for the admission, which then is used to calculate the eGFR for CKD staging and for AKI identification and staging (S4 Figure). Patients with ESKD are excluded from this algorithm. The algorithm is run for every creatinine measurement identified in the inpatient encounter. First the algorithm determines if the creatinine measurement that has triggered this run of the algorithm was obtained within the first seven days of the admission. If the index creatinine measurement is from the first seven days of the admission, a list of all serum creatinine levels with time and date stamps is used to calculate the reference creatinine. If there were previous creatinine measurements in the interval 0–7 days before admission we used the minimum creatinine level during that interval as reference value 1. If there were previous creatinine measurements in the interval 8–365 days before admission, we used the median creatinine level during that interval as reference value 2 [11, 14, 15]. The reference creatinine is then the minimum of (reference value 1, reference value 2 and the admission creatinine) (S4 Figure). For patients with no history of CKD, the reference creatinine is the minimum of (reference value 1, reference value 2, the admission creatinine, estimated creatinine). Estimated creatinine values are obtained by back-calculation from existing formulas assuming that baseline eGFR is 75 ml/min per 1.73 m^2^. We compared results using three methods of estimating creatinine to examine effect of race adjustment for African Americans. Race-agnostic algorithm 1 calculated estimated creatinine by back-calculation from the Modification of Diet in Renal Disease Study (MDRD) equation with race modifier removed. Race-agnostic algorithm 2 calculated estimated creatinine by back-calculation from the 2021 CKD-EPI fit without race [16]. Race-adjusted algorithm calculated the estimated creatinine by back-calculation from the original Modification of Diet in Renal Disease Study equation with race multiplier. For encounters with preadmission CKD, but no preadmission or admission creatinine, the first creatinine of the encounter was used as the reference creatinine to determine the first AKI status and stage of the encounter, but eGFR calculation and CKD staging was not done. For days with no serum creatinine measurement, AKI stage was imputed carrying forward last available AKI stage. If the index creatinine measurement is from eight or more days after admission, the algorithm identifies the last available reference creatinine if the patient had AKI on prior day or the minimum creatinine from the previous seven days as the reference creatinine, otherwise. We also examined the impact of the race multiplier for African Americans using three methods on the AKI status and stage through its effect on the reference creatinine.

1. Determination of CKD stage

**3.1. G-Staging of CKD:**

The CKD Staging Flow is used to determine the patients’ G-stage of CKD based on a calculated eGFR using reference creatinine and the Chronic Kidney Disease Epidemiology Collaboration equation (CKD-EPI) [17] formula and 2021 CKD-EPI fit (S5 Figure). The reference serum creatinine level was calculated by the Reference Creatinine Flow as explained in Section 2. For encounters with preadmission CKD, but no preadmission or admission creatinine, reference creatinine was assumed to be missing for eGFR calculation and CKD staging. In order to examine the impact of the race adjustment for African Americans in the CKD-EPI formula, we compared CKD status and CKD G-stage using three approaches. Race-adjusted algorithm calculated eGFR using 2009 CKD-EPI formula that has a race multiplier [17]. Race-agnostic algorithm 1 estimated eGFR using the Chronic Kidney Disease Epidemiology CKD-EPI with race modifier removed. Race-agnostic algorithm 2 calculated estimated creatinine using the 2021 CKD-EPI fit without race.

**3.2. A-Staging of CKD:**

As first approach, we used equations for converting urine PCR to urine ACR and urine dipstick protein to urine ACR on the basis of meta-analyzed associations of same-day measures from the crude model provided by Sumida et al. [18] Patients were categorized as stage 1 if predicted ACR<30, as stage 2 if predicted ACR was in range 30 and 300, and as stage 3 if predicted ACR>300.

As second approach, we developed a stepwise approach as detailed below as shown in S6 Figure. Below are the steps of assigning A-stages. First, we cleaned all urine laboratory measurements within one year prior to admission including admission calendar day with Logical Observation Identifier Names and Codes (LOINC) measurements as provided in S14 Table. We converted values to standard unit, as appropriate, if there were other units used. When albumin or microalbumin and urine creatinine samples were obtained at the same time, they were used to calculate UACR. Similarly, UPCR was also calculated urine protein and urine creatinine samples obtained at the same time. We first found albumin excretion rate (AER) and urine albumin-to-creatinine ratio (UACR) measurements and assigned A stage to A1 if there was at least one measurement of AER or UACR <30 mg/g, to A2 if there was at least one measurement of AER or UACR between 30 and 300 mg/g, or to A3 otherwise. If there were no AER or UACR values available, we used multinomial logistic models we developed that use logarithm of urine protein-to-creatinine ratio (UPCR) to determine probability of being in each A-stage and assign to the class with highest probability. If there are no AER, UACR, or UPCR values available, we used urine protein (UAP) values. UAP values that were not already in the form negative, trace, 1+, 2+, 3+, or 4+ were categorized into groups as negative for UAP<10, trace for 10≤UAP<30, 1+ for 30≤UAP<100 or “small,” 2+ for 100≤UAP<300 or “moderate” or “medium,” 3+ for 300≤UAP<1000 or “large,” and 4+ for UAP≥1000. We used multinomial logistic models that we developed and that use normalized values of UAP and specific gravity to determine probability of being in each A-stage and assign to the class with highest probability. Multinomial logistic models developed are provided below:

UPCR classifier

- a1= exp(11.397 – 2.006 * log(UPCR))
- a2= exp(1.492 – 0.023 * log(UPCR))
- a3= exp(-12.889 + 2.029 * log(UPCR))
- P(A1) = a1 / (a1 + a2 + a3)
- P(A2) = a2 / (a1 + a2 + a3)
- P(A3) = a3 / (a1 + a2 + a3)
- A_stage = max(P(A1), P(A2), P(A3))

UAP classifier

- a1 = exp( – 1.009 + 0.354 * norm(if 1+) -0.812 * norm(if 2+) – 1.077 * norm(if 3+ or more) +1.265 * norm(if negative) – 0.056 * norm(if trace) + 1.185 * norm(SG))
- a2 = exp(1.109 + 0.146 * norm(if 1+) + 0.062 * norm(if 2+) – 0.335 * norm(if 3+ or more) + 0.018 * norm(if negative) + 0.177 * norm(if trace) – 0.053 * norm(SG))
- a3 = exp(-0.1 - 0.499 * norm(if 1+) +0.75 * norm(if 2+) + 1.412 * norm(if 3+ or more) -1.283 * norm(if negative) – 0.122 * norm(if trace) – 1.133 * norm(SG))
- P(A1) = a1 / (a1 + a2 + a3)
- P(A2) = a2 / (a1 + a2 + a3)
- P(A3) = a3 / (a1 + a2 + a3)
- A_stage = max(P(A1), P(A2), P(A3))

where norm represents normalized value using the parameters as in below table for each level of UAP and for SG, where SG stands for specific gravity.

| - **Feature name** | - **Mean** | - **Variance** |
| --- | --- | --- |
| - If 1+ | - 0.25 | - 0.1875 |
| - If 2+ | - 0.314 | - 0.2155 |
| - If 3+ or more | - 0.125 | - 0.109 |
| - If negative | - 0.307 | - 0.213 |
| - If trace | - 0.0036 | - 0.004 |
| - Sg | - 1.016 | - 0.00007 |

1. Identification and staging of AKI and renal recovery

The AKI Identification Flow is used to determine if the patient has any evidence of current AKI by KDIGO serum creatinine criteria (0.3 mg/dl increase in serum creatinine within 48 hours or 50% increase from baseline) or by requirement for KRT, and to identify the presence and trajectory of any AKI based on the duration of AKI and any renal recovery (S7-S8 Figures). This algorithm is triggered to run by every new measurements of serum creatinine in an inpatient encounter. We defined an episode of AKI as beginning when this algorithm identifies AKI and ending if there are two consecutive days without AKI identified, thus allowing us to identify a new episode of AKI in a patient who has recovered from a previous episode of AKI. We determined if the patient had persistent AKI or AKD by KDIGO and ADQI criteria, as well as the recovery trajectory [8, 9] (S8 Figure). An episode of AKI that resolves completely within 48 hours is termed “rapid reversal” AKI and is believed to have minimal clinical consequences. An episode of AKI that persists beyond 48 hours is described as “persistent” AKI, while an episode of AKI that results in renal dysfunction persisting beyond seven days is described using the new term “Acute Kidney Disease” (AKD). Renal dysfunction persisting 90 days or longer is CKD, and CKD resulting in a need for KRT is ESKD. Patients with ESKD can undergo renal transplantation that leaves them with normal renal function or with less-severe CKD that does not require KRT.

With the AKI Staging Flow, we determined the KDIGO AKI stage for all patients identified with AKI algorithm. If the patient was undergoing KRT, the AKI stage is “Stage 3 with KRT.” If the patient was not undergoing KRT, the current reference creatinine is used to stage the AKI (S8 Figure). Stage 1 AKI is defined as a minimum of 0.3 mg/dl absolute increase in serum creatinine in 48 hours or rises to at least 1.5-fold from reference creatinine but less than two-fold. Stage 2 is defined as rises to at least two-fold but less than three-fold, and Stage 3 is defined as rises to at least three-fold from baseline or dialysis. Renal replacement status was determined daily, based on Current Procedural Terminology (CPT) codes and data elements from EHR flowsheet with order details and fluid management for hemodialysis, peritoneal dialysis, and continuous kidney replacement therapies (S8–S10 Tables). Since we have already excluded patients with ESKD, if the patient is undergoing KRT, identified by CPT code or by clinical evidence in the EHR, we assumed that the patient is currently being treated for AKI. In order to examine the impact of the race multiplier on AKI status and stages, we examined changes in classification of AKI status and stages when race modifier is included in the MDRD formula that is part of the reference creatinine for no CKD patients compared to race-agnostic algorithm 1 and 2.

Phenotype Algorithm Validation

The algorithms were tested and validated by comparing the performance of the phenotype in identifying patients with AKI and CKD to clinical diagnosis of these conditions by clinical experts doing chart review of the EHR. Adjudication by clinician experts, with access to all patient data within a medical chart, to identify patients with disease is used as the gold standard. We enlisted three physicians (a nephrologist, an internist, and a surgeon) and a medical student trained in the clinical consensus definitions of AKI and CKD to independently review the validation cohort of patients to determine if the patients had CKD at the time of admission and/or AKI that developed during the hospitalization. Reviewers used physician notes, nursing notes, and laboratory results to search for clinical evidence of CKD and/or AKI. Any differences in ascertainment of either CKD or AKI were arbitrated in discussion between the four reviewers while reviewing the EHR for the patient. Sensitivity, specificity, positive and negative predictive values, and overall accuracy (the proportion of true classification including true positives plus true negatives) for ascertainment by the phenotype compared to ascertainment by clinical review were calculated with exact binomial confidence intervals. Cases where there was a mismatch between identification by phenotype compared to identification by clinician were reviewed again to determine how the phenotype might be improved in the future.

The review sample for the phenotype algorithm validation was created by selecting inpatient encounters admitted between January 2012 and April 2016 from the *AKI EPIC* database based on CKD status while stratifying each group into three groups by AKI status and renal recovery. We determined that a total sample size of 300 with 67% of patients having AKI would achieve 80% power to detect a change in sensitivity from 0.9 to 0.95 and 81% power to detect a change in specificity from 0.9 to 0.97 using a two-sided binomial test at significance level of 0.05. The review sample for the phenotype algorithm validation was created by selecting 300 inpatient encounters, half with CKD and half with no CKD, while stratifying each group into three groups by AKI status and renal recovery (no AKI, AKI with renal recovery, and AKI without renal recovery). We selected proportional number of patients in each subgroup for review. Half of the patients in each subgroup were selected among the relevant group in the cohort with the highest and the other half of the patients were selected among the ones with the lowest reference creatinine values. The medical record numbers for those patient encounters were pulled and used to review each patient’s record in the Epic EHR. Statistical analyses were performed with SAS (version 9.4; SAS Institute, Inc, Cary, NC), Python (version 3.7), and R software (version 3.5.1).

**SUPPLEMENTARY REFERENCES**

1. Bihorac A, Brennan M, Ozrazgat-Baslanti T, Bozorgmehri S, Efron PA, Moore FA, et al. National surgical quality improvement program underestimates the risk associated with mild and moderate postoperative acute kidney injury. Crit Care Med. 2013;41(11):2570-83. Epub 2013/08/10. doi: 10.1097/CCM.0b013e31829860fc. PubMed PMID: 23928835; PubMed Central PMCID: PMCPMC3812338.

2. Adhikari L, Ozrazgat-Baslanti T, Ruppert M, Madushani R, Paliwal S, Hashemighouchani H, et al. Improved predictive models for acute kidney injury with IDEA: Intraoperative Data Embedded Analytics. PLoS One. 2019;14(4):e0214904. Epub 2019/04/05. doi: 10.1371/journal.pone.0214904. PubMed PMID: 30947282; PubMed Central PMCID: PMCPMC6448850.

3. Bihorac A, Ozrazgat-Baslanti T, Ebadi A, Motaei A, Madkour M, Pardalos PM, et al. MySurgeryRisk: Development and Validation of a Machine-learning Risk Algorithm for Major Complications and Death After Surgery. Ann Surg. 2019;269(4):652-62. Epub 2018/03/01. doi: 10.1097/sla.0000000000002706. PubMed PMID: 29489489; PubMed Central PMCID: PMCPMC6110979.

4. Ozrazgat-Baslanti T, Thottakkara P, Huber M, Berg K, Gravenstein N, Tighe P, et al. Acute and Chronic Kidney Disease and Cardiovascular Mortality After Major Surgery. Ann Surg. 2016;264(6):987-96. Epub 2016/01/13. doi: 10.1097/sla.0000000000001582. PubMed PMID: 26756753; PubMed Central PMCID: PMCPMC4936961.

5. Korenkevych D, Ozrazgat-Baslanti T, Thottakkara P, Hobson CE, Pardalos P, Momcilovic P, et al. The Pattern of Longitudinal Change in Serum Creatinine and 90-Day Mortality After Major Surgery. Ann Surg. 2016;263(6):1219-27. Epub 2015/07/17. doi: 10.1097/SLA.0000000000001362. PubMed PMID: 26181482; PubMed Central PMCID: PMCPMC4829495.

6. Gardner AK, Ghita GL, Wang Z, Ozrazgat-Baslanti T, Raymond SL, Mankowski RT, et al. The Development of Chronic Critical Illness Determines Physical Function, Quality of Life, and Long-Term Survival Among Early Survivors of Sepsis in Surgical ICUs. Crit Care Med. 2019. Epub 2019/01/22. doi: 10.1097/ccm.0000000000003655. PubMed PMID: 30664526.

7. Loftus TJ, Mira JC, Ozrazgat-Baslanti T, Ghita GL, Wang Z, Stortz JA, et al. Sepsis and Critical Illness Research Center investigators: protocols and standard operating procedures for a prospective cohort study of sepsis in critically ill surgical patients. BMJ Open. 2017;7(7):e015136. Epub 2017/08/03. doi: 10.1136/bmjopen-2016-015136. PubMed PMID: 28765125; PubMed Central PMCID: PMCPMC5642775.

8. Chawla LS, Bellomo R, Bihorac A, Goldstein SL, Siew ED, Bagshaw SM, et al. Acute kidney disease and renal recovery: consensus report of the Acute Disease Quality Initiative (ADQI) 16 Workgroup. Nat Rev Nephrol. 2017.

9. Khwaja A. KDIGO clinical practice guidelines for acute kidney injury. Nephron Clinical practice. 2012;120(4):c179-84. Epub 2012/08/15. doi: 10.1159/000339789. PubMed PMID: 22890468.

10. KDIGO 2017 Clinical Practice Guideline Update for the Diagnosis, Evaluation, Prevention, and Treatment of Chronic Kidney Disease-Mineral and Bone Disorder (CKD-MBD). Kidney international supplements. 2017;7(1):1-59. Epub 2017/07/01. doi: 10.1016/j.kisu.2017.04.001. PubMed PMID: 30675420; PubMed Central PMCID: PMCPMC6340919.

11. Ozrazgat-Baslanti T, Motaei A, Islam R, Hashemighouchani H, Ruppert M, Madushani R, et al. Development and validation of computable Phenotype to Identify and Characterize Kidney Health in Adult Hospitalized Patients. arXiv preprint arXiv:190303149. 2019.

12. Wald R, Waikar SS, Liangos O, Pereira BJ, Chertow GM, Jaber BL. Acute renal failure after endovascular vs open repair of abdominal aortic aneurysm. J Vasc Surg. 2006;43(3):460-6; discussion 6. doi: 10.1016/j.jvs.2005.11.053. PubMed PMID: 16520155.

13. Kidney Disease: Improving Global Outcomes (KDIGO) CKD Work Group. KDIGO 2012 clinical practice guideline for the evaluation and management of chronic kidney disease. Kidney inter, Suppl. 2013;3(1):1-150.

14. Holmes J, Roberts G, Meran S, Williams JD, Phillips AO, Welsh AKISG. Understanding Electronic AKI Alerts: Characterization by Definitional Rules. Kidney Int Rep. 2017;2(3):342-9. doi: 10.1016/j.ekir.2016.12.001. PubMed PMID: 29142963; PubMed Central PMCID: PMCPMC5678680.

15. Selby NM, Hill R, Fluck RJ, Programme NHSETKA. Standardizing the Early Identification of Acute Kidney Injury: The NHS England National Patient Safety Alert. Nephron. 2015;131(2):113-7. Epub 2015/09/10. doi: 10.1159/000439146. PubMed PMID: 26351847.

16. Inker LA, Eneanya ND, Coresh J, Tighiouart H, Wang D, Sang Y, et al. New Creatinine- and Cystatin C-Based Equations to Estimate GFR without Race. N Engl J Med. 2021;385(19):1737-49. Epub 2021/09/24. doi: 10.1056/NEJMoa2102953. PubMed PMID: 34554658.

17. Levey AS, Stevens LA, Schmid CH, Zhang YL, Castro AF, 3rd, Feldman HI, et al. A new equation to estimate glomerular filtration rate. Ann Intern Med. 2009;150(9):604-12. PubMed PMID: 19414839; PubMed Central PMCID: PMCPMC2763564.

18. Sumida K, Nadkarni GN, Grams ME, Sang Y, Ballew SH, Coresh J, et al. Conversion of Urine Protein-Creatinine Ratio or Urine Dipstick Protein to Urine Albumin-Creatinine Ratio for Use in Chronic Kidney Disease Screening and Prognosis : An Individual Participant-Based Meta-analysis. Ann Intern Med. 2020;173(6):426-35. Epub 2020/07/14. doi: 10.7326/M20-0529. PubMed PMID: 32658569.

19. Nadkarni GN, Gottesman O, Linneman JG, Chase H, Berg RL, Farouk S, et al., editors. Development and validation of an electronic phenotyping algorithm for chronic kidney disease. AMIA Annu Symp Proc; 2014: American Medical Informatics Association.

20. Norton JM, Ali K, Jurkovitz CT, Kiryluk K, Park M, Kawamoto K, et al. Development and Validation of a Pragmatic Electronic Phenotype for CKD. Clin J Am Soc Nephrol. 2019;14(9):1306-14. Epub 2019/08/14. doi: 10.2215/CJN.00360119. PubMed PMID: 31405830; PubMed Central PMCID: PMCPMC6730512.

21. Shang N, Khan A, Polubriaginof F, Zanoni F, Mehl K, Fasel D, et al. Medical records-based chronic kidney disease phenotype for clinical care and "big data" observational and genetic studies. NPJ Digit Med. 2021;4(1):70. Epub 2021/04/15. doi: 10.1038/s41746-021-00428-1. PubMed PMID: 33850243; PubMed Central PMCID: PMCPMC8044136.

22. Wilson FP, Martin M, Yamamoto Y, Partridge C, Moreira E, Arora T, et al. Electronic health record alerts for acute kidney injury: multicenter, randomized clinical trial. BMJ. 2021;372:m4786. Epub 2021/01/20. doi: 10.1136/bmj.m4786. PubMed PMID: 33461986; PubMed Central PMCID: PMC8034420.

23. Menon S, Tarrago R, Carlin K, Wu H, Yonekawa K. Impact of integrated clinical decision support systems in the management of pediatric acute kidney injury: a pilot study. Pediatr Res. 2021;89(5):1164-70. Epub 2020/07/04. doi: 10.1038/s41390-020-1046-8. PubMed PMID: 32620006.

24. Gubb S, Holmes J, Smith G, Geen J, Williams J, Donovan K, et al. Acute Kidney Injury in Children Based on Electronic Alerts. J Pediatr. 2020;220:14-20 e4. Epub 2020/01/21. doi: 10.1016/j.jpeds.2019.11.019. PubMed PMID: 31955879.

25. Holmes J, Donovan K, Geen J, Williams J, Phillips AO. Acute kidney injury demographics and outcomes: changes following introduction of electronic acute kidney injury alerts-an analysis of a national dataset. Nephrol Dial Transplant. 2021;36(8):1433-9. Epub 2020/06/10. doi: 10.1093/ndt/gfaa071. PubMed PMID: 32514532.

26. Bataineh A, Dealmeida D, Bilderback A, Ambrosino R, Al-Jaghbeer MJ, Fuhrman DY, et al. Sustained effects of a clinical decision support system for acute kidney injury. Nephrol Dial Transplant. 2020;35(10):1819-21. Epub 2020/06/24. doi: 10.1093/ndt/gfaa099. PubMed PMID: 32572486; PubMed Central PMCID: PMCPMC7824807.

27. Park S, Baek SH, Ahn S, Lee KH, Hwang H, Ryu J, et al. Impact of Electronic Acute Kidney Injury (AKI) Alerts With Automated Nephrologist Consultation on Detection and Severity of AKI: A Quality Improvement Study. Am J Kidney Dis. 2018;71(1):9-19. Epub 2017/07/30. doi: 10.1053/j.ajkd.2017.06.008. PubMed PMID: 28754457.

28. Holmes J, Roberts G, May K, Tyerman K, Geen J, Williams JD, et al. The incidence of pediatric acute kidney injury is increased when identified by a change in a creatinine-based electronic alert. Kidney Int. 2017;92(2):432-9. Epub 2017/05/10. doi: 10.1016/j.kint.2017.03.009. PubMed PMID: 28483379.

29. Meersch M, Schmidt C, Hoffmeier A, Van Aken H, Wempe C, Gerss J, et al. Prevention of cardiac surgery-associated AKI by implementing the KDIGO guidelines in high risk patients identified by biomarkers: the PrevAKI randomized controlled trial. Intensive Care Med. 2017;43(11):1551-61. Epub 2017/01/23. doi: 10.1007/s00134-016-4670-3. PubMed PMID: 28110412; PubMed Central PMCID: PMCPMC5633630.

30. Al-Jaghbeer M, Dealmeida D, Bilderback A, Ambrosino R, Kellum JA. Clinical Decision Support for In-Hospital AKI. J Am Soc Nephrol. 2018;29(2):654-60. Epub 2017/11/04. doi: 10.1681/ASN.2017070765. PubMed PMID: 29097621; PubMed Central PMCID: PMCPMC5791078.

31. Chandrasekar T, Sharma A, Tennent L, Wong C, Chamberlain P, Abraham KA. A whole system approach to improving mortality associated with acute kidney injury. QJM. 2017;110(10):657-66. Epub 2017/05/19. doi: 10.1093/qjmed/hcx101. PubMed PMID: 28521019.

32. Ebah L, Hanumapura P, Waring D, Challiner R, Hayden K, Alexander J, et al. A Multifaceted Quality Improvement Programme to Improve Acute Kidney Injury Care and Outcomes in a Large Teaching Hospital. BMJ Qual Improv Rep. 2017;6(1). Epub 2017/06/14. doi: 10.1136/bmjquality.u219176.w7476. PubMed PMID: 28607684; PubMed Central PMCID: PMCPMC5457974.

33. Bedford M, Stevens P, Coulton S, Billings J, Farr M, Wheeler T, et al. Development of risk models for the prediction of new or worsening acute kidney injury on or during hospital admission: a cohort and nested study. Health Services and Delivery Research. Southampton (UK)2016.

34. Koyner JL, Adhikari R, Edelson DP, Churpek MM. Development of a Multicenter Ward-Based AKI Prediction Model. Clin J Am Soc Nephrol. 2016;11(11):1935-43. Epub 2016/09/17. doi: 10.2215/CJN.00280116. PubMed PMID: 27633727; PubMed Central PMCID: PMCPMC5108182.

35. Prendecki M, Blacker E, Sadeghi-Alavijeh O, Edwards R, Montgomery H, Gillis S, et al. Improving outcomes in patients with Acute Kidney Injury: the impact of hospital based automated AKI alerts. Postgrad Med J. 2016;92(1083):9-13. Epub 2015/10/30. doi: 10.1136/postgradmedj-2015-133496. PubMed PMID: 26512125; PubMed Central PMCID: PMCPMC4717457.

36. Kolhe NV, Reilly T, Leung J, Fluck RJ, Swinscoe KE, Selby NM, et al. A simple care bundle for use in acute kidney injury: a propensity score-matched cohort study. Nephrol Dial Transplant. 2016;31(11):1846-54. Epub 2016/11/04. doi: 10.1093/ndt/gfw087. PubMed PMID: 27190331.

37. Kolhe NV, Staples D, Reilly T, Merrison D, McIntyre CW, Fluck RJ, et al. Impact of Compliance with a Care Bundle on Acute Kidney Injury Outcomes: A Prospective Observational Study. PLoS One. 2015;10(7):e0132279. Epub 2015/07/15. doi: 10.1371/journal.pone.0132279. PubMed PMID: 26161979; PubMed Central PMCID: PMCPMC4498890.

38. Wilson FP, Shashaty M, Testani J, Aqeel I, Borovskiy Y, Ellenberg SS, et al. Automated, electronic alerts for acute kidney injury: a single-blind, parallel-group, randomised controlled trial. Lancet. 2015;385(9981):1966-74. Epub 2015/03/03. doi: 10.1016/S0140-6736(15)60266-5. PubMed PMID: 25726515; PubMed Central PMCID: PMCPMC4475457.

39. Ahmed A, Vairavan S, Akhoundi A, Wilson G, Chiofolo C, Chbat N, et al. Development and validation of electronic surveillance tool for acute kidney injury: A retrospective analysis. J Crit Care. 2015;30(5):988-93. Epub 2015/06/14. doi: 10.1016/j.jcrc.2015.05.007. PubMed PMID: 26070247.

40. Bell S, Dekker FW, Vadiveloo T, Marwick C, Deshmukh H, Donnan PT, et al. Risk of postoperative acute kidney injury in patients undergoing orthopaedic surgery--development and validation of a risk score and effect of acute kidney injury on survival: observational cohort study. BMJ. 2015;351:h5639. Epub 2015/11/13. doi: 10.1136/bmj.h5639. PubMed PMID: 26561522; PubMed Central PMCID: PMCPMC4641433.

41. Claus BO, Colpaert K, Steurbaut K, De Turck F, Vogelaers DP, Robays H, et al. Role of an electronic antimicrobial alert system in intensive care in dosing errors and pharmacist workload. Int J Clin Pharm. 2015;37(2):387-94. Epub 2015/02/11. doi: 10.1007/s11096-015-0075-6. PubMed PMID: 25666942.

42. Flynn N, Dawnay A. A simple electronic alert for acute kidney injury. Ann Clin Biochem. 2015;52(Pt 2):206-12. Epub 2014/04/26. doi: 10.1177/0004563214534832. PubMed PMID: 24763854.

43. Thomas ME, Sitch A, Baharani J, Dowswell G. Earlier intervention for acute kidney injury: evaluation of an outreach service and a long-term follow-up. Nephrol Dial Transplant. 2015;30(2):239-44. Epub 2014/10/23. doi: 10.1093/ndt/gfu316. PubMed PMID: 25335505.
