# Supplementary material for: Development and validation of a race-agnostic computable phenotype for kidney health in adult hospitalized patients
Source: PLoS One. 2024 Apr 23;19(4):e0299332. doi: 10.1371/journal.pone.0299332 (PMC11037544; doi:10.1371/journal.pone.0299332)
Supplement: S1 Table — (DOCX) [file pone.0299332.s002.docx]

**S1 Table. Summary of studies on chronic kidney disease (CKD) phenotyping**

| **Study** | **Study Design** | **Region and Time Period** | **Real-time implementation** | **CDM** | **Public Data**  **Set** | **CKD Definition** | **Lab Standard (LOINCs)** | **ICD codes for CKD provided** | **CKD staging criteria** | **Race-agnostic** |
| --- | --- | --- | --- | --- | --- | --- | --- | --- | --- | --- |
| Nadkarni (2014)[19] | Retrospective | Development:  Mount Sinai Hospital (2003-2014)  Validation: Marshfield Clinic Research (1985-2014)  Columbia University  Medical Center (since 1990-2014) | No | NA | Yes | Based on 2012 KDIGO criteria for defining CKD stage 3 or higher:  If has ICD code for CKD (585.xx) and GFR ≤ 60 ml/min/1.73 m^2^  OR  two recent GFR lab tests ≤ 60 ml/min/1.73 m^2^ (3 months apart) | NA | Yes | eGFR-staging | No |
| Norton (2019)[20] | Retrospective | Five health care organizations (Christiana Care, Columbia University, University of  Minnesota, UCSF, University of UTAH) | No | NA | NA | Most recent eGFR<60 ml/min per 1.73 m^2^ with at least one value <60 ml/min/1.73 m^2^ 90+ days prior  AND/OR  a UACR of ≥30 mg/g in the most recent test with at least  one positive value 90+ days prior | Yes | Yes | eGFR-staging | No |
| Shang (2021)[21] | Retrospective | Development: Columbia University (CU), University of Minnesota (UMN), Vanderbilt University (VU), and Mayo Clinic (MC) Validation: Columbia Clinical Data Warehouse (CDW) (1997-2017), the entire Electronic Medical Records and Genomics-III (eMERGE-III) network of eight centers with  genetic and EHR data. | No | Can be easily customized to different data models | Yes | Based on combination of NKF KDOQ, KDIGO, and domain expert knowledge:  Most recent eGFR<90 ml/min per 1.73 m2 with at least one value <90 ml/min per 1.73 m2 more than 3 months prior  OR  Has CKD or relevant disease diagnosis codes  OR  Or A1-stage on the latest urine test | Yes | Yes | eGFR and A-staging | No |
