# Supplementary material for: Development and validation of a race-agnostic computable phenotype for kidney health in adult hospitalized patients
Source: PLoS One. 2024 Apr 23;19(4):e0299332. doi: 10.1371/journal.pone.0299332 (PMC11037544; doi:10.1371/journal.pone.0299332)
Supplement: S2 Table — (DOCX) [file pone.0299332.s003.docx]

**S2 Table. Performance of studies on CKD phenotyping**

| **Study** | **Study Design** | **Region and Time Period** | **Validation Sample Size** | **Sensitivity**  **(95% confidence interval)** | **Specificity**  **(95% confidence interval)** | **PPV**  **(95% confidence interval)** | **NPV**  **(95% confidence interval)** |
| --- | --- | --- | --- | --- | --- | --- | --- |
| Nadkarni (2014)[19] | Retrospective | Development:  Mount Sinai Hospital (2003–2014)  Validation: Mount Sinai Hospital (2003–2014), Marshfield Clinic Research (1985–2014)  Columbia University  Medical Center (since 1990–2014) | 1,186 (Mount Sinai Hospital), 100 (Marshfield Clinic), 100 (Columbia University  Medical Center) | NA | NA | 96% (94% –97%) | 93% (91%–95%) |
| Norton (2019)[20] | Retrospective | Five health care organizations (Christiana Care, Columbia University, University of  Minnesota, UCSF, University of UTAH) | 207 (University of Minnesota:58, Christiana Care:71, Columbia University:60, UCSF:18) | 99% (96%–100%) | 99% (92%–100%) | 99% (96%–100%) | 99% (92%–100%) |
| Shang (2021)[21] | Retrospective | Development: Columbia University (CU), University of Minnesota (UMN), Vanderbilt University (VU), and Mayo Clinic (MC) Validation: Columbia Clinical Data Warehouse (CDW) (1997–2017), the entire Electronic Medical Records and Genomics-III (eMERGE-III) network of eight centers with  genetic and EHR data. | 1,136 CKD, 1,214 healthy | 87% | 97% | 97% | 89% |

Abbreviations. CKD, chronic kidney disease; CDM, common data model; LOINC, Logical Observation Identifiers Names and Codes; ICD, International Classification of Diseases.
