# Supplementary material for: Development and validation of a race-agnostic computable phenotype for kidney health in adult hospitalized patients
Source: PLoS One. 2024 Apr 23;19(4):e0299332. doi: 10.1371/journal.pone.0299332 (PMC11037544; doi:10.1371/journal.pone.0299332)
Supplement: S3 Table — (DOCX) [file pone.0299332.s004.docx]

**S3 Table. Summary of studies on AKI alerts**

| **Study** | **Real time implementation** | **Study Design** | **Region and Time Period** | **CDM** | **Public Data Set** | **Race Agnostic** | **Lab Standard (LOINCs)** | **AKI Definition** | **Baseline sCr Definition** | **Labeling Frequency** | **Predictive Task** |
| --- | --- | --- | --- | --- | --- | --- | --- | --- | --- | --- | --- |
| Wilson (2021)[22] | Yes | Double blinded, multicenter, randomized, controlled trial | Six hospitals in the Yale New Haven Health System in Connecticut and Rhode Island, US (29 March 2018 to 14 December 2019) | No | Yes | NA | NA | Based on KDIGO criteria | The lowest creatinine within the prior 7 days of hospitalization | Alert is fired whenever the patient’s electronic chart was opened. | NA |
| Menon (2021)[23] | Yes | Prospective, non-randomized | Seattle Children's Hospital, US (January 2018-October 2018) | No | No | NA | NA | Based on KDIGO criteria | The lowest creatinine in 6 months prior to admission (if available) or calculated via modified Schwartz equation | For each sCr | NA |
| Gubb (2020)[24] | Yes | Prospective | Wales (April 2015-March 2019) | No | No | NA | NA | Based on KDIGO criteria | The lowest creatinine of 7 days or median of 8-365 days prior to admission or population reference interval | For each sCr | NA |
| Holmes (2020)[25] | Yes | Prospective | Wales (April 2015-March 2019) | No | No | NA | NA | Based on KDIGO criteria | The lowest creatinine in last 7 days | For each sCr | NA |
| Bataineh (2020)[26] | No | Follow-up | University of Pittsburgh Medical Center System, US (January 2016-March 2018) | No | No | NA | NA | Based on KDIGO criteria | The lowest creatinine in 12 months prior to admission or back-calculation using MDRD if no CKD history. If the patient has CKD history admission sCr is used as baseline. | 4-hour time-period | NA |
| Selby (2019)[15] | Yes | Multi-center stepped-wedge cluster randomized trial | Five UK hospitals (December 2014-November 2016) | No | No | NA | NA | Based on KDIGO criteria | The lowest creatinine in the last 7 days or median of value of 8 to 365 days prior to admission. | For each sCr | NA |
| Park (2018)[27] | Yes | Before-after | A tertiary referral hospital, Korea (usual care group: 1 January-31 December 2013; alert group: 1 June 2014- 31 May 2015) | No | No | NA | NA | Based on KDIGO criteria | The lowest creatinine within 2 weeks before admission or first value measured during hospitalization | Every midnight | NA |
| Holmes (2017)[28] | Yes | Prospective | Wales (1 November 2013- 30 April 2016) | No | No | NA | NA | Based on KDIGO criteria | Alternative baseline sCr calculations considered: 1) With eCCl 120 ml/min/1.73 m2 and back calculation or 2) midpoint normative creatinine value for age and sex or 3) national algorithm | For each sCr | NA |
| Meersch (2017)[29] | Not alert based | Single center, randomized controlled trial | University of Muenster, Germany (August 2014-December 2015) | No | No | NA | NA | Based on KDIGO criteria | Mean preoperative creatinine | 4-h after cardiac surgery | NA |
| Al-Jaghbeer (2017)[30] | Yes | Prospective (sequential period analysis) | University of Pittsburgh Medical Center, US (Before CDSS: October of 2012-September of 2013, after CDSS: October 2013- September 2015) | No | No | NA | NA | Based on KDIGO criteria | The lowest creatinine within 12 months before admission or back-calculation using MDRD if no CKD history. If the patient has CKD history, admission sCr is used as baseline. | 4-hour time-period | NA |
| Chandrasekar (2017)[31] | Yes | Statistical process control analysis (interventional) | Aintree University Hospital, UK (Before alert: January 2011– October 2013, after alert: October 2013- July 2016) | No | No | NA | NA | Based on AKIN criteria | The lowest creatinine within 7 days prior admission or median creatinine value of 8-365 days prior to admission. | For each sCr | NA |
| Ebah (2017)[32] | Yes | Interventional, before and after | Central Manchester University Hospital, Manchester, UK (Before quality improvement plan: November 2013-January 2015, after quality improvement plan improvement: March 2015-June 2016) | No | No | NA | NA | Condition: there is a greater than 50% rise in serum creatinine from a baseline value. | NA | For each sCr (reported daily) | Yes |
| Bedford (2016)[33] | Yes | Retrospective | East Kent Hospitals University NHS Foundation Trust (2011) and Medway NHS Foundation Trust, UK (as a secondary population for validation) | No | No | NA | NA | Based on KDIGO criteria | Median of all creatinine values in 8-365 days prior to admission or the lowest creatinine in 7 days or the lowest creatinine in 48 hours prior to admission. | 1.At admission to hospital  2. Following initial admission blood tests in the first 24 hours  3. At 72nd hour | Yes |
| Koyner (2016)[34] | No | Prospective | University of Chicago and the Electronic Data Warehouse at NorthShore University Health System, US (November of 2008-January of 2013) | No | No | NA | NA | Based on KDIGO criteria | Defined as the first SCr measured on hospital admission, and this baseline value was updated on a rolling basis as per the KDIGO SCr criteria | Every 12 hours | Yes |
| Prendecki (2016)[35] | Yes | Retrospective | NHS hospitals, UK (20 April 2012-20 September 2013) | No | No | NA | NA | Based on KDIGO criteria | The last available creatinine value | For each sCr | NA |
| Kolhe (2016)[36] | Yes | Prospective, propensity score–matched controlled | Derby Teaching Hospitals, UK (1 August 2013-31 January 2015) | No | No | NA | NA | AKIN criteria | The lowest stable creatinine value from previous 12 months (except the most recent last 48 hours) is used; if possible, a baseline from the last 3 months is preferred. | For each sCr | NA |
| Kolhe (2015)[37] | Yes | Prospective, observational | Royal Derby Hospital, UK (February 2013-December 2013) | No | No | NA | NA | Based on KDIGO criteria | The lowest stable creatinine value from previous 12 months (except the most recent last 48 hours) is used; if possible, a baseline from the last 3 months is preferred. | For each sCr | NA |
| Wilson (2015)[38] | Yes | Parallel group randomized control | University of Pennsylvania Hospital (17 Sept 2013- 14 April 2014) | No | No | NA | NA | Based on KDIGO criteria | The lowest value that occurred in the previous 48 h or the lowest value that occurred in the previous 7 days | Hourly basis | NA |
| Ahmed (2015)[39] | Yes | Retrospective, development and validation | Mayo Clinic ICU, Rochester, MN. (Derivation cohort: July 1, 2010-December 31, 2010; Validation cohort: January 12, 2010-March 23, 2010) | No | No | Race coefficient used | NA | Based on AKIN criteria | Median of all the creatinine values during 180 days prior to ICU admission or MDRD formula | 15 minutes | NA |
| Bell (2015)[40] | No | Observational | Ninewells Hospital, Dundee, Scotland (1 January 2005-31 December 2011) | No | No | NA | NA | Based on KDIGO criteria | Most recent before surgery as preoperative measurement and maximal serum creatinine concentration during the first seven postoperative days as post-measurement. | Once | NA |
| Claus (2015)[41] | Yes | Single center prospective, observational | Ghent University Hospital, Ghent, Belgium (28 February 2008–11 April 2008) | No | No | NA | NA | NA | Mean Scr at the beginning and the end of the 24-h period (for calculating CLcr) | For every prescription each day | NA |
| Flynn (2015)[42] | Yes | Retrospective | University College London Hospitals NHS Foundation Trust (time period was not given) | No | No | NA | NA | Based on AKIN criteria | Previous and subsequent creatinine results | For each sCr | NA |
| Thomas (2015)[43] | Yes | Prospective, before and after study | Two UK hospitals (Before: 15 May 2009-12 June 2009, After: 12 June-31 July 2009) | No | No | NA | NA | Staging based on RIFLE criteria | No imputed baseline values were used. The RIFLE stage for each patient was determined using the percent rise from baseline to peak creatinine for the episode of AKI (retrospectively determined) | For each sCr | NA |

Abbreviations. AKIN, Acute Kidney Injury Network; CDM, common data model; CDSS, clinical decision support system; CKD-EPI, Chronic Kidney Disease Epidemiology Collaboration; eGFR, estimated glomerular filtrate rate; KDIGO, Kidney Disease Improving Global Outcomes; MDRD, Modification of Diet in Renal Disease; RIFLE, Risk, Injury, Failure, Loss of kidney function and End-stage kidney disease; sCr, serum creatinine
