# Supplementary material for: Development and validation of a race-agnostic computable phenotype for kidney health in adult hospitalized patients
Source: PLoS One. 2024 Apr 23;19(4):e0299332. doi: 10.1371/journal.pone.0299332 (PMC11037544; doi:10.1371/journal.pone.0299332)
Supplement: S4 Table — (DOCX) [file pone.0299332.s005.docx]

**S4 Table. Data elements that are used to run CKD phenotyping algorithm**

| **Features** | **Description** | **Format** |
| --- | --- | --- |
| patient_deiden_id | Deidentified Patient ID | String |
| encounter_deiden_id | Deidentified Encounter ID | String |
| admit_datetime | Hospital Admission Date and Time | Date and Time |
| dischg_datetime | Discharge Date and Time | Date and Time |
| birth_date | Birth Date | Date and Time |
| Sex | Sex | String |
| Race | Race | String |
| Ethnicity | Ethnicity | String |
| patient_type | Patient Type | String |
| start_date | Diagnosis Start Date | Date |
| diag_code | Diagnosis Code | String |
| diag_icd_type | Diagnosis Code Type (ICD9, ICD10) | String |
| proc_date | Procedure Date | Date |
| proc_code_type | Procedure Code Type (ICD9, ICD10, CPT) | String |
| proc_code | Procedure Code | String |
| lab_result | Lab Result | Float |
| lab_unit | Lab Unit | String |
| inferred_specimen_datetime | Inferred Specimen Taken Date and Time | Date and Time |
| stamped_and_inferred_loinc_code | Stamped and Inferred LOINC Code | String |

Abbreviations. ICD-9-CM, International Classification of Diseases, 9th revision, clinical modification; ICD-10-CM, International Classification of Diseases, 10th revision, clinical modification; CPT, Current Procedural Terminology; LOINC, Logical Observation Identifier Names and Codes.
