# Supplementary material for: Development and validation of a race-agnostic computable phenotype for kidney health in adult hospitalized patients
Source: PLoS One. 2024 Apr 23;19(4):e0299332. doi: 10.1371/journal.pone.0299332 (PMC11037544; doi:10.1371/journal.pone.0299332)
Supplement: S5 Table — (DOCX) [file pone.0299332.s006.docx]

**S5** **Table. Data elements that are used to run AKI phenotyping algorithm**

| **Features** | **Description** | **Format** |
| --- | --- | --- |
| patient_deiden_id | Deidentified Patient ID | String |
| encounter_deiden_id | Deidentified Encounter ID | String |
| proc_code | Procedure code | String |
| Proc_code_type | Procedure code type (ICD9, ICD10, CPT) | String |
| proc_date | Date for procedure code | Date |
| vital_sign_measure_name^a^ | The type of vital signs measured | String |
| meas_value^a^ | The measured value of the vital | String |
| recorded_time^a^ | The date and time of measurement | Date and Time |
| hemodialysis_intake^a^ | Intake value for hemodialysis | Float |
| hemodialysis_output^a^ | Output value for hemodialysis | Float |
| peritoneal_dialysis_intake^a^ | Intake value for Peritoneal Dialysis | Float |
| peritoneal_dialysis_output^a^ | Output value for Peritoneal Dialysis | Float |
| observation_datetime^a^ | Date and time for measurement for dialysis intake and output | Date and Time |
| lab_result | Lab Result | Float |
| lab_unit | Lab Unit | String |
| inferred_specimen_datetime | Inferred Specimen Taken Date and Time | Date and Time |
| stamped_and_inferred_loinc_code | Stamped and Inferred LOINC Code | String |

^a^ Data elements that are used for determining dialysis status in addition to CPT codes. Patient is considered to be on dialysis when the type of vital signs measured is “Treatment Type” and the measured value of the vital is *CVVH* or *CVVHD* or *CVVHDF* or if there is non-zero hemodialysis or peritoneal dialysis intake or output.
