# Supplementary material for: Development and validation of a race-agnostic computable phenotype for kidney health in adult hospitalized patients
Source: PLoS One. 2024 Apr 23;19(4):e0299332. doi: 10.1371/journal.pone.0299332 (PMC11037544; doi:10.1371/journal.pone.0299332)
Supplement: S6 Table — (DOCX) [file pone.0299332.s007.docx]

**S6 Table. Administrative codes used for end stage kidney disease**

| **ICD Code** | **Explanation** |
| --- | --- |
| **ICD-9-CM Diagnosis** |  |
| 585.6 | End stage kidney disease |
| V45.1 | Renal dialysis status  Excludes: admission for dialysis treatment or session (V56.0) |
| V45.11 | Renal dialysis status  Hemodialysis status  Patient requiring intermittent renal dialysis  Peritoneal dialysis status  Presence of arterial-venous shunt (for dialysis) |
| V45.12 | Noncompliance with renal dialysis |
| **ICD-10-CM Diagnosis** |  |
| N18.6 | End stage kidney disease |
| Z91.15 | Patient's noncompliance with renal dialysis |
