# Supplementary material for: Development and validation of a race-agnostic computable phenotype for kidney health in adult hospitalized patients
Source: PLoS One. 2024 Apr 23;19(4):e0299332. doi: 10.1371/journal.pone.0299332 (PMC11037544; doi:10.1371/journal.pone.0299332)
Supplement: S7 Table — (DOCX) [file pone.0299332.s008.docx]

**S7** **Table. Administrative codes used for chronic kidney disease**

| **ICD Code** | **Explanation** |
| --- | --- |
| **ICD-9-CM Diagnosis** |  |
| 403.00 | Hypertensive chronic kidney disease, malignant, with chronic kidney disease stage I through stage IV, or unspecified |
| 403.01 | Hypertensive chronic kidney disease, malignant, with chronic kidney disease stage V or end stage kidney disease |
| 403.10 | Hypertensive chronic kidney disease, benign, with chronic kidney disease stage I through stage IV, or unspecified |
| 403.11 | Hypertensive chronic kidney disease, benign, with chronic kidney disease stage V or end stage kidney disease |
| 403.90 | Hypertensive chronic kidney disease, unspecified, with chronic kidney disease stage I through stage IV, or unspecified |
| 403.91 | Hypertensive chronic kidney disease, unspecified, with chronic kidney disease stage V or end stage kidney disease |
| 404.00 | Hypertensive heart and chronic kidney disease, malignant, without heart failure and with chronic kidney disease stage I through stage IV, or unspecified |
| 404.01 | Hypertensive heart and chronic kidney disease, malignant, with heart failure and with chronic kidney disease stage I through stage IV, or unspecified |
| 404.02 | Hypertensive heart and chronic kidney disease, malignant, without heart failure and with chronic kidney disease stage V or end stage kidney disease |
| 404.03 | Hypertensive heart and chronic kidney disease, malignant, with heart failure and with chronic kidney disease stage V or end stage kidney disease |
| 404.10 | Hypertensive heart and chronic kidney disease, benign, without heart failure and with chronic kidney disease stage I through stage IV, or unspecified |
| 404.11 | Hypertensive heart and chronic kidney disease, benign, with heart failure and with chronic kidney disease stage I through stage IV, or unspecified |
| 404.12 | Hypertensive heart and chronic kidney disease, benign, without heart failure and with chronic kidney disease stage V or end stage kidney disease |
| 404.13 | Hypertensive heart and chronic kidney disease, benign, with heart failure and chronic kidney disease stage V or end stage kidney disease |
| 404.90 | Hypertensive heart and chronic kidney disease, unspecified, without heart failure and with chronic kidney disease stage I through stage IV, or unspecified |
| 404.91 | Hypertensive heart and chronic kidney disease, unspecified, with heart failure and with chronic kidney disease stage I through stage IV, or unspecified |
| 404.92 | Hypertensive heart and chronic kidney disease, unspecified, without heart failure and with chronic kidney disease stage V or end stage kidney disease |
| 404.93 | Hypertensive heart and chronic kidney disease, unspecified, with heart failure and chronic kidney disease stage V or end stage kidney disease |
| 581 | Nephrotic syndrome |
| 581.0 | Nephrotic syndrome with lesion of proliferative glomerulonephritis |
| 581.1 | Nephrotic syndrome with lesion of membranous glomerulonephritis |
| 581.2 | Nephrotic syndrome with lesion of membranoproliferative glomerulonephritis |
| 581.3 | Nephrotic syndrome with lesion of minimal change glomerulonephritis |
| 581.8 | Nephrotic syndrome with other specified pathological lesion in kidney |
| 581.81 | Nephrotic syndrome in diseases classified elsewhere |
| 581.89 | Nephrotic syndrome with other specified pathological lesion in kidney |
| 581.9 | Nephrotic syndrome with unspecified pathological lesion in kidney |
| 582 | Chronic glomerulonephritis |
| 582.0 | Chronic glomerulonephritis with lesion of proliferative glomerulonephritis |
| 582.1 | Chronic glomerulonephritis with lesion of membranous glomerulonephritis |
| 582.2 | Chronic glomerulonephritis with lesion of membranoproliferative glomerulonephritis |
| 582.4 | Chronic glomerulonephritis with lesion of rapidly progressive glomerulonephritis |
| 582.8 | Chronic glomerulonephritis with other specified pathological lesion in kidney |
| 582.81 | Chronic glomerulonephritis in diseases classified elsewhere |
| 582.89 | Chronic glomerulonephritis with other specified pathological lesion in kidney |
| 582.9 | Chronic glomerulonephritis with unspecified pathological lesion in kidney |
| 583 | Nephritis and nephropathy, not specified as acute or chronic |
| 583.0 | Nephritis and nephropathy, not specified as acute or chronic, with lesion of proliferative glomerulonephritis |
| 583.1 | Nephritis and nephropathy, not specified as acute or chronic, with lesion of membranous glomerulonephritis |
| 583.2 | Nephritis and nephropathy, not specified as acute or chronic, with lesion of membranoproliferative glomerulonephritis |
| 583.4 | Nephritis and nephropathy, not specified as acute or chronic, with lesion of rapidly progressive glomerulonephritis |
| 583.6 | Nephritis and nephropathy, not specified as acute or chronic, with lesion of renal cortical necrosis |
| 583.7 | Nephritis and nephropathy, not specified as acute or chronic, with lesion of renal medullary necrosis |
| 583.8 | Nephritis and nephropathy not specified as acute or chronic with other specified pathological lesion in kidney |
| 583.81 | Nephritis and nephropathy, not specified as acute or chronic, in diseases classified elsewhere |
| 583.89 | Nephritis and nephropathy, not specified as acute or chronic, with other specified pathological lesion in kidney |
| 583.9 | Nephritis and nephropathy, not specified as acute or chronic, with unspecified pathological lesion in kidney |
| 585 | Chronic kidney disease (ckd) |
| 585.1 | Chronic kidney disease, Stage I |
| 585.2 | Chronic kidney disease, Stage II (mild) |
| 585.3 | Chronic kidney disease, Stage III (moderate) |
| 585.4 | Chronic kidney disease, Stage IV (severe) |
| 585.9 | Chronic kidney disease, unspecified |
| 586 | Renal failure, unspecified |
| 250.40 | Diabetes with renal manifestations, type II or unspecified type, not stated as uncontrolled |
| 250.41 | Diabetes with renal manifestations, type I [juvenile type], not stated as uncontrolled |
| 250.42 | Diabetes with renal manifestations, type II or unspecified type, uncontrolled |
| 250.43 | Diabetes with renal manifestations, type I [juvenile type], uncontrolled |
| 588.8 | Other specified disorders resulting from impaired renal function |
| 588.81 | Secondary hyperparathyroidism (of renal origin) |
| 588.89 | Other specified disorders resulting from impaired renal function |
| 588.9 | Unspecified disorder resulting from impaired renal function |
| 753.13 | Polycystic kidney, autosomal dominant |
| **ICD-10-CM Diagnosis** |  |
| I12.0 | Hypertensive chronic kidney disease with stage 5 chronic kidney disease or end stage kidney disease |
| I12.9 | Hypertensive chronic kidney disease with stage 1 through stage 4 chronic kidney disease, or unspecified chronic kidney disease |
| I13.0 | Hypertensive heart and chronic kidney disease with heart failure and stage 1 through stage 4 chronic kidney disease, or unspecified chronic kidney disease |
| I13.1 | Hypertensive heart and chronic kidney disease without heart failure |
| I13.10 | Hypertensive heart and chronic kidney disease without heart failure with stage 1 through stage 4 chronic kidney disease, or unspecified chronic kidney disease |
| I13.11 | Hypertensive heart and chronic kidney disease without heart failure with stage 5 chronic kidney disease, or end stage kidney disease |
| I13.2 | Hypertensive heart and chronic kidney disease with heart failure and with stage 5 chronic kidney disease, or end stage kidney disease |
| N01 | Rapidly progressive nephritic syndrome |
| N01.0 | Rapidly progressive nephritic syndrome with minor glomerular abnormality |
| N01.1 | Rapidly progressive nephritic syndrome with focal and segmental glomerular lesions |
| N01.2 | Rapidly progressive nephritic syndrome with diffuse membranous glomerulonephritis |
| N01.3 | Rapidly progressive nephritic syndrome with diffuse mesangial proliferative glomerulonephritis |
| N01.4 | Rapidly progressive nephritic syndrome with diffuse endocapillary proliferative glomerulonephritis |
| N01.5 | Rapidly progressive nephritic syndrome with diffuse mesangiocapillary glomerulonephritis |
| N01.6 | Rapidly progressive nephritic syndrome with dense deposit disease |
| N01.7 | Rapidly progressive nephritic syndrome with diffuse crescentic glomerulonephritis |
| N01.8 | Rapidly progressive nephritic syndrome with other morphologic changes |
| N01.9 | Rapidly progressive nephritic syndrome with unspecified morphologic changes |
| N02.0 | Recurrent and persistent hematuria with minor glomerular abnormality |
| N02.1 | Recurrent and persistent hematuria with focal and segmental glomerular lesions |
| N02.2 | Recurrent and persistent hematuria with diffuse membranous glomerulonephritis |
| N02.3 | Recurrent and persistent hematuria with diffuse mesangial proliferative glomerulonephritis |
| N02.4 | Recurrent and persistent hematuria with diffuse endocapillary proliferative glomerulonephritis |
| N02.5 | Recurrent and persistent hematuria with diffuse mesangiocapillary glomerulonephritis |
| N02.6 | Recurrent and persistent hematuria with dense deposit disease |
| N02.7 | Recurrent and persistent hematuria with diffuse crescentic glomerulonephritis |
| N02.8 | Recurrent and persistent hematuria with other morphologic changes |
| N02.9 | Recurrent and persistent hematuria with unspecified morphologic changes |
| N03 | Chronic nephritic syndrome |
| N03.0 | Chronic nephritic syndrome with minor glomerular abnormality |
| N03.1 | Chronic nephritic syndrome with focal and segmental glomerular lesions |
| N03.2 | Chronic nephritic syndrome with diffuse membranous glomerulonephritis |
| N03.3 | Chronic nephritic syndrome with diffuse mesangial proliferative glomerulonephritis |
| N03.4 | Chronic nephritic syndrome with diffuse endocapillary proliferative glomerulonephritis |
| N03.5 | Chronic nephritic syndrome with diffuse mesangiocapillary glomerulonephritis |
| N03.6 | Chronic nephritic syndrome with dense deposit disease |
| N03.7 | Chronic nephritic syndrome with diffuse crescentic glomerulonephritis |
| N03.8 | Chronic nephritic syndrome with other morphologic changes |
| N03.9 | Chronic nephritic syndrome with unspecified morphologic changes |
| N04 | Nephrotic syndrome |
| N04.0 | Nephrotic syndrome with minor glomerular abnormality |
| N04.1 | Nephrotic syndrome with focal and segmental glomerular lesions |
| N04.2 | Nephrotic syndrome with diffuse membranous glomerulonephritis |
| N04.3 | Nephrotic syndrome with diffuse mesangial proliferative glomerulonephritis |
| N04.4 | Nephrotic syndrome with diffuse endocapillary proliferative glomerulonephritis |
| N04.5 | Nephrotic syndrome with diffuse mesangiocapillary glomerulonephritis |
| N04.6 | Nephrotic syndrome with dense deposit disease |
| N04.7 | Nephrotic syndrome with diffuse crescentic glomerulonephritis |
| N04.8 | Nephrotic syndrome with other morphologic changes |
| N04.9 | Nephrotic syndrome with unspecified morphologic changes |
| N05 | Unspecified nephritic syndrome |
| N05.0 | Unspecified nephritic syndrome with minor glomerular abnormality |
| N05.1 | Unspecified nephritic syndrome with focal and segmental glomerular lesions |
| N05.2 | Unspecified nephritic syndrome with diffuse membranous glomerulonephritis |
| N05.3 | Unspecified nephritic syndrome with diffuse mesangial proliferative glomerulonephritis |
| N05.4 | Unspecified nephritic syndrome with diffuse endocapillary proliferative glomerulonephritis |
| N05.5 | Unspecified nephritic syndrome with diffuse mesangiocapillary glomerulonephritis |
| N05.6 | Unspecified nephritic syndrome with dense deposit disease |
| N05.7 | Unspecified nephritic syndrome with diffuse crescentic glomerulonephritis |
| N05.8 | Unspecified nephritic syndrome with other morphologic changes |
| N05.9 | Unspecified nephritic syndrome with unspecified morphologic changes |
| N06 | Isolated proteinuria with specified morphological lesion |
| N06.0 | Isolated proteinuria with minor glomerular abnormality |
| N06.1 | Isolated proteinuria with focal and segmental glomerular lesions |
| N06.2 | Isolated proteinuria with diffuse membranous glomerulonephritis |
| N06.3 | Isolated proteinuria with diffuse mesangial proliferative glomerulonephritis |
| N06.4 | Isolated proteinuria with diffuse endocapillary proliferative glomerulonephritis |
| N06.5 | Isolated proteinuria with diffuse mesangiocapillary glomerulonephritis |
| N06.6 | Isolated proteinuria with dense deposit disease |
| N06.7 | Isolated proteinuria with diffuse crescentic glomerulonephritis |
| N06.8 | Isolated proteinuria with other morphologic lesion |
| N06.9 | Isolated proteinuria with unspecified morphologic lesion |
| N07 | Hereditary nephropathy, not elsewhere classified |
| N07.0 | Hereditary nephropathy, not elsewhere classified with minor glomerular abnormality |
| N07.1 | Hereditary nephropathy, not elsewhere classified with focal and segmental glomerular lesions |
| N07.2 | Hereditary nephropathy, not elsewhere classified with diffuse membranous glomerulonephritis |
| N07.3 | Hereditary nephropathy, not elsewhere classified with diffuse mesangial proliferative glomerulonephritis |
| N07.4 | Hereditary nephropathy, not elsewhere classified with diffuse endocapillary proliferative glomerulonephritis |
| N07.5 | Hereditary nephropathy, not elsewhere classified with diffuse mesangiocapillary glomerulonephritis |
| N07.6 | Hereditary nephropathy, not elsewhere classified with dense deposit disease |
| N07.7 | Hereditary nephropathy, not elsewhere classified with diffuse crescentic glomerulonephritis |
| N07.8 | Hereditary nephropathy, not elsewhere classified with other morphologic lesions |
| N07.9 | Hereditary nephropathy, not elsewhere classified with unspecified morphologic lesions |
| E08.2 | Diabetes mellitus due to underlying condition with kidney complications |
| E08.21 | Diabetes mellitus due to underlying condition with diabetic nephropathy |
| E08.22 | Diabetes mellitus due to underlying condition with diabetic chronic kidney disease |
| E08.29 | Diabetes mellitus due to underlying condition with other diabetic kidney complication |
| E09.2 | Drug or chemical induced diabetes mellitus with kidney complications |
| E09.21 | Drug or chemical induced diabetes mellitus with diabetic nephropathy |
| E09.22 | Drug or chemical induced diabetes mellitus with diabetic chronic kidney disease |
| E09.29 | Drug or chemical induced diabetes mellitus with other diabetic kidney complication |
| E10.2 | Type 1 diabetes mellitus with kidney complications |
| E10.21 | Type 1 diabetes mellitus with diabetic nephropathy |
| E10.22 | Type 1 diabetes mellitus with diabetic chronic kidney disease |
| E10.29 | Type 1 diabetes mellitus with other diabetic kidney complication |
| E11.2 | Type 2 diabetes mellitus with kidney complications |
| E11.21 | Type 2 diabetes mellitus with diabetic nephropathy |
| E11.22 | Type 2 diabetes mellitus with diabetic chronic kidney disease |
| E11.29 | Type 2 diabetes mellitus with other diabetic kidney complication |
| E13.2 | Other specified diabetes mellitus with kidney complications |
| E13.21 | Other specified diabetes mellitus with diabetic nephropathy |
| E13.22 | Other specified diabetes mellitus with diabetic chronic kidney disease |
| E13.29 | Other specified diabetes mellitus with other diabetic kidney complication |
| N25 | Disorders resulting from impaired renal tubular function |
| N25.8 | Other disorders resulting from impaired renal tubular function |
| N25.81 | Secondary hyperparathyroidism of renal origin |
| N25.89 | Other disorders resulting from impaired renal tubular function |
| N25.9 | Disorder resulting from impaired renal tubular function, unspecified |
| Q61 | Cystic kidney disease |
| Q61.2 | Polycystic kidney, adult type |
| Q61.3 | Polycystic kidney, unspecified |
| Q61.4 | Renal dysplasia |
| Q61.5 | Medullary cystic kidney |
| Q61.8 | Other cystic kidney diseases |
| Q61.9 | Cystic kidney disease, unspecified |
| N18.1 | Chronic kidney disease, stage 1 |
| N18.2 | Chronic kidney disease, stage 2 (mild) |
| N18.3 | Chronic kidney disease, stage 3 (moderate) |
| N18.4 | Chronic kidney disease, stage 4 (severe) |
| N18.5 | Chronic kidney disease, stage 5 |
| N18.9 | Chronic kidney disease, unspecified |
| N28 | Other disorders of kidney and ureter, not elsewhere classified |
| N28.0 | Ischemia and infarction of kidney |
