# Supplementary material for: Development and validation of a race-agnostic computable phenotype for kidney health in adult hospitalized patients
Source: PLoS One. 2024 Apr 23;19(4):e0299332. doi: 10.1371/journal.pone.0299332 (PMC11037544; doi:10.1371/journal.pone.0299332)
Supplement: S8 Table — (DOCX) [file pone.0299332.s009.docx]

**S8** **Table. Administrative codes for kidney transplant**

| **ICD Code** | **Explanation** |
| --- | --- |
| **ICD-9-CM Diagnosis** |  |
| V42.0 | Kidney replaced by transplant |
| 996.81 | Complications of transplanted kidney |
| **ICD-9-CM Procedure** |  |
| 55.6 | Transplant of Kidney |
| 55.61 | Renal autotransplantation |
| 55.69 | Other kidney transplantation |
| **ICD-10-CM Diagnosis** |  |
| Z94.0 | Kidney transplant status |
| T86.10 | Unspecified complication of kidney transplant |
| T86.11 | Kidney transplant rejection |
| T86.12 | Kidney transplant failure |
| T86.13 | Kidney transplant infection |
| T86.19 | Other complication of kidney transplant |
| **ICD-10-PCS Procedure** |  |
| 0TS00ZZ | Reposition Right Kidney, Open Approach |
| 0TS10ZZ | Reposition Left Kidney, Open Approach |
| 0TY00Z0 | Transplantation of Right Kidney, Allogeneic, Open Approach |
| 0TY00Z1 | Transplantation of Right Kidney, Syngeneic, Open Approach |
| 0TY10Z0 | Transplantation of Left Kidney, Allogeneic, Open Approach |
| 0TY10Z1 | Transplantation of Left Kidney, Syngeneic, Open Approach |
| **CPT** |  |
| 50360 | Renal allotransplantation, implantation of graft; without recipient nephrectomy |
| 50365 | Renal allotransplantation, implantation of graft; with recipient nephrectomy |
| 50380 | Renal autotransplantation, reimplantation of kidney |
