# Supplementary material for: Development and validation of a race-agnostic computable phenotype for kidney health in adult hospitalized patients
Source: PLoS One. 2024 Apr 23;19(4):e0299332. doi: 10.1371/journal.pone.0299332 (PMC11037544; doi:10.1371/journal.pone.0299332)
Supplement: S9 Table — (DOCX) [file pone.0299332.s010.docx]

**S9 Table. Administrative codes used for renal-replacement therapy**

| **ICD Code** | **Explanation** |
| --- | --- |
| **ICD-9-CM Diagnosis** |  |
| V45.12 | Noncompliance with renal dialysis |
| V56.0 | Encounter for extracorporeal dialysis |
| V56.8 | Encounter for other dialysis |
| V56.1 | Fitting and adjustment of extracorporeal dialysis catheter |
| V56.2 | Fitting and adjustment of peritoneal dialysis catheter |
| V56.32 | Encounter for adequacy testing for peritoneal dialysis |
| V45.1 | Renal dialysis status |
| V45.11 | Renal dialysis status |
| 996.56 | Mechanical complication due to peritoneal dialysis catheter |
| 996.68 | Infection and inflammatory reaction due to peritoneal dialysis catheter |
| 792.5 | Cloudy (hemodialysis) (peritoneal) dialysis effluent |
| **ICD-9-CM Procedure** |  |
| 39.95 | Hemodialysis |
| 54.98 | Peritoneal dialysis |
| **ICD-10-CM Diagnosis** |  |
| Z91.15 | Patient's noncompliance with renal dialysis |
| Z49.31 | Encounter for adequacy testing for hemodialysis |
| Z49.32 | Encounter for adequacy testing for peritoneal dialysis |
| Z49.01 | Encounter for fitting and adjustment of extracorporeal dialysis catheter |
| Z49.02 | Encounter for fitting and adjustment of peritoneal dialysis catheter |
| Z49.32 | Encounter for adequacy testing for peritoneal dialysis |
| T85.71XA | Infection and inflammatory reaction due to peritoneal dialysis catheter, initial encounter |
| T85.611A | Breakdown (mechanical) of intraperitoneal dialysis catheter, initial encounter |
| T85.621A | Displacement of intraperitoneal dialysis catheter, initial encounter |
| R88.0 | Cloudy (hemodialysis) (peritoneal) dialysis effluent |
| T85.631A | Leakage of intraperitoneal dialysis catheter, initial encounter |
| T85.71XA | Infection and inflammatory reaction due to peritoneal dialysis catheter |
| T85.71XS | Infection and inflammatory reaction due to peritoneal dialysis catheter, sequela |
| Z99.2 | Dependence on renal dialysis |
| T85.71XD | Infection and inflammatory reaction due to peritoneal dialysis catheter, subsequent encounter |
| **ICD-10-PCS Procedure** |  |
| 5A1D00Z | Performance of Urinary Filtration, Single |
| 5A1D60Z | Performance of Urinary Filtration, Multiple |
| 5A1D70Z | Performance of Urinary Filtration, Intermittent, Less than 6 Hours Per Day |
| 5A1D80Z | Performance of Urinary Filtration, Prolonged Intermittent, 6-18 hours Per Day |
| 5A1D90Z | Performance of Urinary Filtration, Continuous, Greater than 18 hours Per Day |
| 3E1M39Z | Irrigation of Peritoneal Cavity using Dialysate, Percutaneous Approach |
| **CPT** |  |
| 90935 | Hemodialysis procedure with single evaluation by a physician or other qualified health care professional |
| 90937 | Hemodialysis procedure requiring repeated evaluation(s) with or without substantial revision of dialysis prescription |
| 90945 | Dialysis procedure other than hemodialysis (e.g., peritoneal dialysis, hemofiltration, or other continuous renal replacement therapies), with single evaluation by a physician or other qualified health care professional |
| 90947 | Dialysis procedure other than hemodialysis (e.g., peritoneal dialysis, hemofiltration, or other continuous renal replacement therapies) requiring repeated evaluations by a physician or other qualified health care professional, with or without substantial revision of dialysis prescription |
| 90999 | Unlisted dialysis procedure, inpatient or outpatient |
