# Supplementary material for: Development and validation of a race-agnostic computable phenotype for kidney health in adult hospitalized patients
Source: PLoS One. 2024 Apr 23;19(4):e0299332. doi: 10.1371/journal.pone.0299332 (PMC11037544; doi:10.1371/journal.pone.0299332)
Supplement: S10 Table — (DOCX) [file pone.0299332.s011.docx]

**S10** **Table. ICD codes used for history of acute kidney injury (AKI)**

| **ICD Code** | **Explanation** |
| --- | --- |
| **ICD-9-CM Diagnosis** |  |
| 584 | Acute kidney failure |
| 584.5 | Acute kidney failure with lesion of tubular necrosis convert |
| 584.6 | Acute kidney failure with lesion of renal cortical necrosis convert |
| 584.7 | Acute kidney failure with lesion of renal medullary [papillary] necrosis |
| 584.8 | Acute kidney failure with other specified pathological lesion in kidney |
| 584.9 | Acute kidney failure, unspecified |
| 593.9 | Unspecified disorder of kidney and ureter (includes renal disease (chronic) not otherwise specified) |
| 997.5 | Urinary complications, not elsewhere classified |
| **ICD-10-CM Diagnosis** |  |
| N17 | Acute kidney failure |
| N17.0 | Acute kidney failure with tubular necrosis |
| N17.1 | Acute kidney failure with acute cortical necrosis |
| N17.2 | Acute kidney failure with medullary necrosis |
| N17.8 | Other acute kidney failure |
| N17.9 | Acute kidney failure, unspecified |
| N28.9 | Disorder of kidney and ureter, unspecified |
