# Supplementary material for: Development and validation of a race-agnostic computable phenotype for kidney health in adult hospitalized patients
Source: PLoS One. 2024 Apr 23;19(4):e0299332. doi: 10.1371/journal.pone.0299332 (PMC11037544; doi:10.1371/journal.pone.0299332)
Supplement: S11 Table — (DOCX) [file pone.0299332.s012.docx]

**S11 Table. Details on output categories for CKD algorithm**

| **Flag** | **Flag explanation** | **Group definition** | **Reference creatinine** | **GFR/**  **A Staging** | **Group** | **AKI subgroup prior to admission** |
| --- | --- | --- | --- | --- | --- | --- |
| 1. ESKD | This patient most likely has history of end stage renal disease based on previous records. Please confirm. | Patient has prior ESKD codes and  no renal transplant codes and  admission creatinine / estimated creatinine ≥ 1.5 or missing. | No reference needed,  AKI algorithm does not run. | No | ESKD | NA |
| 1. ESKD with Warning | This patient most likely has history of end stage renal disease based on previous records. Please confirm. | Patient has prior ESKD codes and  no renal transplant codes and  admission creatinine / estimated creatinine <1.5.  Warning issued as some of these patients may be misclassified as ESKD especially if creatinine is not very elevated -need further clarification by physician. | No reference needed,  AKI algorithm does not run. | No | ESKD | NA |
| 1. AKD on admission, CKD after kidney transplant by medical history. | Warning - If no admission creatinine, staging can't be done. Please check serum creatinine. | Patient has AKI codes within 3 months of admission, and renal transplant codes and  admission creatinine / estimated creatinine ≥ 1.5 or missing. Allowed to have ESKD codes prior to renal transplant codes. | CKD algorithm | Yes | CKD | Non-Recovered AKI (AKD) |
| 1. Recovered AKI on admission, CKD after kidney transplant by medical history | Previous recent AKI episode with recovery | Patient has AKI codes within 3 months of admission, and renal transplant codes and  admission creatinine / estimated creatinine < 1.5.  Allowed to have ESKD codes prior to renal transplant codes. | CKD algorithm | Yes | CKD | Recovered AKI |
| 1. CKD after kidney transplant by medical history | This patient has chronic kidney disease after kidney transplant by medical history | Patient has no AKI codes within 3 months of admission  and has renal transplant codes after any ESKD code. | CKD algorithm | Yes | CKD | No AKI |
| 1. CKD by medical history | This patient has chronic kidney disease by medical history | Patient has no AKI codes within 3 months of admission and  no ESKD and  no renal transplant codes and  has CKD codes. | CKD algorithm | Yes | CKD | No AKI |
| 1. Possible AKD on admission, CKD by medical history | Warning –if no admission creatinine, staging can’t be done. Please check serum creatinine. | Patient has AKI codes within 3 months of admission,  no renal transplant codes and  admission creatinine / estimated creatinine ≥ 1.5 or missing. | AKI algorithm | No | CKD | Non-Recovered AKI (AKD) |
| 1. Recovered AKI on admission, CKD by Medical History | Previous recent AKI episode with recovery. | Patient has AKI codes within 3 months of admission and  no renal transplant codes and  no ESKD codes and  has CKD codes and  admission creatinine / estimated creatinine < 1.5. | CKD algorithm | Yes | CKD | Recovered AKI |
| 1. No CKD by any criteria | Patient does not have CKD by medical history and creatinine criteria. | Patient has no AKI codes within 3 months of admission and  no ESKD and  no renal transplant codes and  no CKD codes and  no CKD by creatinine criteria. | AKI algorithm | No | No CKD/ Control | No AKI |
| 1. AKD on admission, no CKD by any criteria. | Warning - If no admission creatinine, staging can't be done. Please check serum creatinine. | Patient has AKI codes within 3 months of admission and  no ESKD and  no renal transplant codes and  no CKD codes and  no CKD by creatinine criteria and  admission creatinine / estimated creatinine ≥ 1.5 or missing. | AKI algorithm | No | No CKD/ Control | Non-Recovered AKI (AKD) |
| 1. Recovered AKI on admission, no CKD by any criteria. | Previous recent AKI episode with recovery. | Patient has AKI codes within 3 months of admission and  no ESKD and  no renal transplant codes and  no CKD codes and  no CKD by creatinine criteria and  no admission creatinine / estimated creatinine < 1.5. | AKI algorithm | Yes | No CKD/ Control | Recovered AKI |
| 1. CKD by Creatinine Criteria | Warning- If no creatinine within 30 days of admission staging can't be done. Please check serum creatinine. | Patient has no AKI codes within 3 months of admission and  no ESKD and  no renal transplant codes and  no CKD codes and  has two eGFR <= 60 separated by at least 3 months (but not within 30 days of admission). | CKD algorithm | Yes | CKD | No AKI |
| 1. AKD on admission, CKD by creatinine criteria. | Warning - If no admission creatinine, staging can't be done. Please check serum creatinine. | Patient has AKI codes within 3 months of admission, and  no ESKD and  no renal transplant codes and  no CKD codes and  has two eGFR <= 60 separated by at least 3 months (but not within 30 days of admission) and  admission creatinine / estimated creatinine ≥ 1.5 or missing. | CKD algorithm | Yes | CKD | Non-Recovered AKI (AKD) |
| 1. Recovered AKI on admission, CKD by creatinine criteria | Previous recent AKI episode with recovery. | Patient has AKI codes within 3 months of admission, and  no ESKD and  no renal transplant codes and  no CKD codes and  has two eGFR <= 60 separated by at least 3 months (but not within 30 days of admission) and  admission creatinine / estimated creatinine < 1.5 . | CKD algorithm | Yes | CKD | Recovered AKI |
| 1. Insufficient Data | Patient has insufficient data to determine CKD by either history or creatinine. | Patient has no ICD codes in the record and  no creatinine measurements. | AKI algorithm | No | Undetermined | Undetermined |

*Race-adjusted algorithm and race-agnostic algorithm calculate estimated creatinine by back-calculation from the Modification of Diet in Renal Disease Study equation with and without race multiplier, respectively. Race-agnostic algorithm 2 calculates estimated creatinine by back calculation from the 2021 CKD-EPI refit without race.
