# Supplementary material for: Development and validation of a race-agnostic computable phenotype for kidney health in adult hospitalized patients
Source: PLoS One. 2024 Apr 23;19(4):e0299332. doi: 10.1371/journal.pone.0299332 (PMC11037544; doi:10.1371/journal.pone.0299332)
Supplement: S12 Table — (DOCX) [file pone.0299332.s013.docx]

**S12** **Table. Methods used to define reference creatinine in validation cohort using race-agnostic and race-adjusted algorithms.**

|  | **Using race-adjusted algorithm,**  **n (%)** | **Using race-agnostic algorithm 1,**  **n (%)** | **Using race-agnostic algorithm 2,**  **n (%)** |
| --- | --- | --- | --- |
| **Method used to determine reference creatinine in all cohort** | N=358,580 | N=358,580 | N= 358,580 |
| Admission creatinine | 177,258 (49) | 173,343 (48) | 179,229 (50) |
| Minimum creatinine in the 7 days prior to admission | 27,383 (8) | 27,060 (8) | 27,713 (8) |
| Median creatinine in 8-365 days prior to admission | 105,542 (29) | 103,742 (28) | 106,217 (27) |
| Estimated creatinine (MDRD or CKD-EPI)^a^ | 47,593 (13) | 53,623 (15) | 44,617 (12) |
| **Among encounters with CKD** | N=84,216 | N=86,329 | N= 82,735 |
| Admission creatinine | 39,506 (47) | 40,516 (47) | 38,844 (50) |
| Minimum creatinine in the 7 days prior to admission | 8,059 (10) | 8,219 (10) | 7,937 (10) |
| Median creatinine in 8-365 days prior to admission | 35,847 (43) | 36,782 (43) | 35,150 (42) |
| Missing | 804 (1) | 812 (1) | 804 (1) |
| **Among encounters with no CKD^b^** | N=274,364 | N=272,251 | N= 275,845 |
| Admission creatinine | 137,752 (50) | 132,827 (49) | 140,385 (51) |
| Minimum creatinine in the 7 days prior to admission | 19,324 (7) | 18,841 (7) | 19,776 (7) |
| Median creatinine in 8-365 days prior to admission | 69,695 (25) | 66,960 (25) | 71,067 (26) |
| Estimated creatinine (MDRD or CKD-EPI) ^a^ | 47,593 (17) | 53,623 (20) | 44,617 (16) |

Abbreviations: CKD, chronic kidney disease; MDRD, Modification of Diet in Renal Disease Study; CKD-EPI, Chronic Kidney Disease Epidemiology Collaboration equation.

^a^ Race-agnostic algorithm 1 estimates creatinine by back-calculation from the Modification of Diet in Renal Disease Study equation without race multiplier. Race-agnostic algorithm 2 estimates creatinine by back calculation from the 2021 CKD-EPI fit without race. Race-adjusted algorithm estimates creatinine by back-calculation from the original Modification of Diet in Renal Disease Study equation with race multiplier.

^b^ Includes group with No CKD with warning due to insufficient data.
