# Supplementary material for: Development and validation of a race-agnostic computable phenotype for kidney health in adult hospitalized patients
Source: PLoS One. 2024 Apr 23;19(4):e0299332. doi: 10.1371/journal.pone.0299332 (PMC11037544; doi:10.1371/journal.pone.0299332)
Supplement: S13 Table — (DOCX) [file pone.0299332.s014.docx]

**S13 Table. Methods used to define reference creatinine in validation cohort using race-agnostic phenotyping algorithms stratified by African American race**

|  | **Using race-agnostic algorithm 1** | | **Using race-agnostic algorithm 2** | |
| --- | --- | --- | --- | --- |
|  | **African American** | **Non-African American** | **African American** | **Non-African American** |
| **Number of encounters** | 86,379 | 272,201 | 86,379 | 272,201 |
| **No CKD by medical history or creatinine criteria, n (%)** | 59,466 (68) | 212,759 (78) | 60,371 (70) | 215,448 (79) |
| **No serum creatinine in past one year or admission ^a^** | 1,995 (3) | 8,904 (4) | 1,997 (3) | 8,889 (4) |
| **Serum creatinine in past one year or admission** | 54,471 (92) | 203,876 (96) | 58,374 (97) | 206,559 (96) |
| **Only admission day** | 19,182 (35) | 76,018 (37) | 19,257 (33) | 76,205 (37) |
| Admission creatinine | 14,055 (73) | 57,999 (76) | 15,463 (80) | 61,194 (80) |
| Estimated creatinine (MDRD or CKD-EPI)^b^ | 5,127 (27) | 18,019 (24) | 3,794 (20) | 15,011 (20) |
| **Only in past one year, and not on admission day** | 4,645 (8) | 21,080 (10) | 4,713 (8) | 21,341 (10) |
| Past creatinine | 3,114 (67) | 16,728 (79) | 4,058 (86) | 17,837 (84) |
| Minimum creatinine in the 7 days prior to admission | 917 (29) | 5,650 (33) | 999 (25) | 6,013 (34) |
| Median creatinine in 8-365 days prior to admission | 2,197 (71) | 11,078 (67) | 3,059 (75) | 11,824 (66) |
| Estimated creatinine (MDRD or CKD-EPI) ^b^ | 931 (33) | 4,352 (21) | 655 (14) | 3,504 (17) |
| **Both in past one year and admission day** | 33,644 (61) | 106,778 (52) | 34,404 (59) | 109,013 (53) |
| Admission creatinine | 14,484 (43) | 46,289 (43) | 15,424 (45) | 48,304 (44) |
| Past creatinine (min 7 or median 8-365) | 15,574 (46) | 49,785 (46) | 16,738 (49) | 52,210 (48) |
| Minimum creatinine in the 7 days prior to admission | 2,305 (15) | 9,969 (20) | 2,414 (14) | 10,350 (20) |
| Median creatinine in 8-365 days prior to admission | 13,269 (85) | 39,816 (80) | 14,324 (86) | 41,860 (80) |
| Estimated creatinine (MDRD or CKD-EPI) ^b^ | 3,586 (11) | 10,704 (10) | 2,242 (7) | 8,499 (8) |
| **CKD by medical history, n (%)** | 23,284 (27) | 48,511 (18) | 23,284 (27) | 48,511 (18) |
| **No serum creatinine in past one year or admission^b^** | 208 (1) | 564 (1) | 208 (1) | 564 (1) |
| **Serum creatinine in past one year or admission** | 23,076 (99) | 47,947 (99) | 23,076 (99) | 47,947 (99) |
| **Only admission day** | 2,924 (13) | 7,640 (16) | 2,924 (13) | 7,640 (16) |
| Admission creatinine | 2,924 (100) | 7,640 (100) | 2,924 (100) | 7,640 (100) |
| Estimated creatinine (MDRD or CKD-EPI) ^b^ | NA | NA | NA (NA) | NA |
| **Only in past one year, and not on admission day** | 1,578 (7) | 4,177 (9) | 1,578 (7) | 4,177 (9) |
| Past creatinine | 1,578(100) | 4,177 (100) | 1,578 (100) | 4,177 (100) |
| Minimum creatinine in the 7 days prior to admission | 393 (25) | 1,144 (27) | 393 (25) | 1,144 (27) |
| Median creatinine in 8-365 days prior to admission | 1,185 (75) | 3,033 (73) | 1,185 (75) | 3,033 (73) |
| Estimated creatinine (MDRD or CKD-EPI) ^b^ | NA | NA | NA | NA |
| **Both in past one year and admission day** | 18,574 (80) | 36,130 (76) | 18,574 (80) | 36,130 (76) |
| Admission creatinine | 8,064 (43) | 15,109 (42) | 8,064 (43) | 15,108 (42) |
| Past creatinine (min 7 or median 8-365) | 10,510 (57) | 21,021 (58) | 10,510 (57) | 21,022 (58) |
| Minimum creatinine in the 7 days prior to admission | 1,718 (16) | 3,728 (18) | 1,718 (16) | 3,728 (18) |
| Median creatinine in 8-365 days prior to admission | 8,792 (84) | 17,293 (82) | 8,792 (84) | 17,294 (82) |
| Estimated creatinine (MDRD or CKD-EPI) ^b^ | NA | NA | NA | NA |
| **CKD by creatinine criteria, n (%)** | 3,624 (4) | 10,910 (4) | 2,719 (3) | 8,221 (3) |
| **No serum creatinine in past one year or admission^c^** | 17 (0.5) | 23 (0.2) | 15 (1) | 17 (0.2) |
| **Serum creatinine in past one year or admission** | 3,607 (99) | 10,887 (99) | 2,704 (99) | 8,204 (99) |
| **Only admission day** | 268 (7) | 640 (6) | 193 (7) | 453 (6) |
| Admission creatinine | 268 (100) | 640 (100) | 193 (100) | 453 (100) |
| Estimated creatinine (MDRD or CKD-EPI) ^b^ | NA | NA | NA | NA |
| **Only in past one year, and not on admission day** | 272 (8) | 933 (9) | 204 (8) | 672 (8) |
| Past creatinine | 272 (100) | 933 (100) | 204 (100) | 672 (100) |
| Minimum creatinine in the 7 days prior to admission | 49 (18) | 193 (20) | 41 (20) | 140 (20) |
| Median creatinine in 8-365 days prior to admission | 223 (82) | 740 (80) | 163 (80) | 532 (80) |
| Estimated creatinine (MDRD or CKD-EPI) ^b^ | NA | NA | NA | NA |
| **Both in past one year and admission day** | 3,067 (85) | 9,314 (85) | 2,307(85) | 7,079 (86) |
| Admission creatinine | 1,490 (48) | 4,381 (47) | 1,143 (50) | 3,319 (47) |
| Past creatinine (min 7 or median 8-365) | 1,577 (52) | 4,933 (53) | 1,164 (52) | 3,760 (53) |
| Minimum creatinine in the 7 days prior to admission | 213 (13) | 781 (16) | 165 (14) | 608 (16) |
| Median creatinine in 8-365 days prior to admission | 1,364 (87) | 4,152 (84) | 999 (86) | 3,152 (84) |
| Estimated creatinine (MDRD or CKD-EPI) ^b^ | NA | NA | NA | NA |
| **Insufficient data, n (%)** | 5 (0.0) | 21 (0.0) | 5 (0.0) | 21 (0.0) |
| **No serum creatinine in past one year or admission ^a^** | 5 (100) | 21 (100) | 5 (100) | 21 (100) |
| **Serum creatinine in past one year or admission** | NA | NA | NA | NA |
| **Only admission day** | NA | NA | NA | NA |
| Admission creatinine | NA | NA | NA | NA |
| Estimated creatinine (MDRD or CKD-EPI) ^b^ | NA | NA | NA | NA |
| **Only in past one year, and not on admission day** | NA | NA | NA | NA |
| Past creatinine | NA | NA | NA | NA |
| Minimum creatinine in the 7 days prior to admission | NA | NA | NA | NA |
| Median creatinine in 8-365 days prior to admission | NA | NA | NA | NA |
| Estimated creatinine (MDRD or CKD-EPI) ^b^ | NA | NA | NA | NA |
| **Both in past one year and admission day** | NA | NA | NA | NA |
| Admission creatinine | NA | NA | NA | NA |
| Past creatinine (min 7 or median 8-365) | NA | NA | NA | NA |
| Minimum creatinine in the 7 days prior to admission | NA | NA | NA | NA |
| Median creatinine in 8-365 days prior to admission | NA | NA | NA | NA |
| Estimated creatinine (MDRD or CKD-EPI) ^b^ | NA | NA | NA | NA |

Abbreviations: CKD, chronic kidney disease; MDRD, Modification of Diet in Renal Disease Study; CKD-EPI, Chronic Kidney Disease Epidemiology Collaboration equation.

^a^ Reference creatinine is estimated by MDRD or 2021 CKD-EPI formulas for all encounters in this group.

^b^ Race-adjusted algorithm and race-agnostic algorithm 1 calculated estimated creatinine by back-calculation from the Modification of Diet in Renal Disease Study equation with and without race multiplier, respectively. Race-agnostic algorithm 2 calculated estimated creatinine by back calculation from the 2021 CKD-EPI refit without race.

^c^ Reference creatinine is missing for this group. The first creatinine of the encounter is used as the reference creatinine to determine the first AKI status and stage of the encounter, but eGFR calculation and CKD staging is not done.
