# Supplementary material for: Development and validation of a race-agnostic computable phenotype for kidney health in adult hospitalized patients
Source: PLoS One. 2024 Apr 23;19(4):e0299332. doi: 10.1371/journal.pone.0299332 (PMC11037544; doi:10.1371/journal.pone.0299332)
Supplement: S14 Table — (DOCX) [file pone.0299332.s015.docx]

**S14** **Table. Logical Observation Identifier Names and Codes (LOINC) codes used for CKD A-staging**

| **LOINC** | **LOINC code description** | **Type** | **Used for calculation of** | **Common Unit** | **To Unit** |
| --- | --- | --- | --- | --- | --- |
| 30003-8 | Microalbumin [Mass/volume] in 24 hour Urine 30003-8 | AER (A24) | AER (A24) | mg/24h | mg/24h |
| 14956-7 | Microalbumin [Mass/time] in 24 hour Urine 14956-7 | AER (A24) | AER (A24) | mg/24h | mg/24h |
| 58448-2 | Microalbumin ug/min [Mass/time] in 24 hour Urine 58448-2 | AER (A24) | AER (A24) | ug/min | mg/24h |
| 49023-5 | Microalbumin [Mass/time] in Urine collected for unspecified duration 49023-5 | AER (A24) | AER (A24) | ug/min | mg/24h |
| 14959-1 | Microalbumin/Creatinine [Mass Ratio] in Urine 14959-1 | UACR | UACR | mg/g Cr | mg/g Cr |
| 9318-7 | Albumin/Creatinine [Mass Ratio] in Urine 9318-7 | UACR | UACR | mg/g Cr | mg/g Cr |
| 14957-5 | Microalbumin [Mass/volume] in Urine 14957-5 | Microalbumin in urine | UACR | mg/dL | mg/dL |
| 1754-1 | Albumin [Mass/volume] in Urine 1754-1 | Albumin in urine and UAP | UACR | mg/dL | mg/dL |
| 2161-8 | Creatinine [Mass/volume] in Urine 2161-8 | Creatinine in Urine | UACR and UPCR | mg/dL | mg/dL |
| 2889-4 | Protein [Mass/time] in 24 hour Urine 2889-4 | PER (P24) | PER (P24) | mg/24h | mg/24h |
| 34539-7 | Protein electrophoresis panel - Urine 34539-7 | Protein in urine | UPCR | mg/dL | mg/dL |
| 2888-6 | Protein [Mass/volume] in Urine 2888-6 | Protein in urine | UPCR | mg/dL | mg/dL |
| 35663-4 | Protein [Mass/volume] in Urine collected for unspecified duration 35663-4 | Protein in urine | UPCR | mg/dL | mg/dL |
| 13801-6 | Protein/Creatinine [Mass Ratio] in 24 hour Urine 13801-6 | UPCR | UPCR | mg/g Cr | mg/g Cr |
| 2890-2 | Protein/Creatinine [Mass Ratio] in Urine 2890-2 | UPCR | UPCR | mg/g Cr | mg/g Cr |
| 20454-5 | Protein [Presence] in Urine by Test strip | UAP | UAP | no unit or mg/dL | no unit |
| 50561-0 | Protein [Mass/volume] in Urine by Automated test strip | UAP | UAP | no unit or mg/dL | no unit |
| 57735-3 | Protein [Presence] in Urine by Automated test strip | UAP | UAP | no unit or mg/dL | no unit |
| 2965-2 | Specific gravity of Urine | SG | SG | no unit | no unit |
| 5811-5 | Specific gravity of Urine by Test strip | SG | SG | no unit | no unit |
| 53326-5 | Specific gravity of Urine by Automated test strip | SG | SG | no unit | no unit |
| 5810-7 | Specific gravity of Urine by Refractometry | SG | SG | no unit | no unit |

Abbreviations. AER, albumin excretion rate; PER, protein excretion rate; UACR, urine albumin-to-creatinine ratio; UAP, urine protein; UPCR, urine protein-to-creatinine ratio; SG, specific gravity.
