# Supplementary material for: Development and validation of a race-agnostic computable phenotype for kidney health in adult hospitalized patients
Source: PLoS One. 2024 Apr 23;19(4):e0299332. doi: 10.1371/journal.pone.0299332 (PMC11037544; doi:10.1371/journal.pone.0299332)
Supplement: S15 Table — (DOCX) [file pone.0299332.s016.docx]

**S15** **Table. Clinical characteristic for patients without ESKD for each cohort**

|  | **DECLARE**  **(Surgical admissions)** | **PICS**  **(Sepsis)** | **AKI EPIC-1**  **(Hospital admissions 1/2012-4/2016)** | **AKI EPIC-2**  **(Hospital admissions 1/2012-8/2021)** |
| --- | --- | --- | --- | --- |
| Number of patients | 49,522 | 239 | 71,934^a^ | 139,152^a^ |
| Age (years), mean (SD) | 56 (17) | 59 (15) | 56 (19) | 54 (19) |
| Female sex, n (%) | 24,307 (49) | 109 (46) | 37,165 (52) | 72,810 (52) |
| African American ethnicity, n (%) | 5,923 (12) | 19 (8) | 12,240 (17) | 24,383 (17) |

^a^ Characteristic for first encounter of each patient is used for the non-ESKD patients with sufficient data to complete AKI phenotyping.
