# Supplementary material for: Development and validation of a race-agnostic computable phenotype for kidney health in adult hospitalized patients
Source: PLoS One. 2024 Apr 23;19(4):e0299332. doi: 10.1371/journal.pone.0299332 (PMC11037544; doi:10.1371/journal.pone.0299332)
Supplement: S16 Table — (DOCX) [file pone.0299332.s017.docx]

**S16** **Table. CKD characteristics using race-adjusted and race-agnostic algorithms**

|  | **Using race-adjusted algorithm,**  **n (%)** | **Using race-agnostic algorithm 1,**  **n (%)** | **Using race-agnostic algorithm 2,**  **n (%)** |
| --- | --- | --- | --- |
| **Overall Patient Encounters** | 358,580 | 358,580 | 358,580 |
| Insufficient Data (No CKD with warning) | 26 (0.0) | 26 (0.0) | 26 (0.0) |
| ***No CKD*** | 274,338 (77) | 272,225 (75) | 275,819 (77) |
| No CKD by Medical History Or Creatinine Criteria and no recent AKI episode^a^ | 265,063 (74) | 263,150 (73) | 266,397 (74) |
| No CKD by Medical History Or Creatinine Criteria, Recovered recent AKI on Admission | 7,395 (2) | 7,156 (2) | 7,566 (2) |
| No CKD by Medical History Or Creatinine Criteria, Non-recovered recent AKI (AKD) on admission | 1,880 (1) | 1,919 (1) | 1,856 (1) |
| ***CKD*** | 84,216 (23) | 86,329 (24) | 82,735 (23) |
| **CKD by Medical History** | 67,123 (19) | 67,123 (19) | 67,123 (19) |
| CKD by Medical History and no recent AKI episode^a^ | 51,590 (14) | 51,590 (14) | 51,590 (14) |
| CKD by Medical History, Recovered recent AKI on Admission | 7,844 (2) | 7,227 (2) | 7,669 (2) |
| CKD by Medical History, Non-recovered recent AKI (AKD) on Admission | 7,689 (2) | 8,306 (2) | 7,864 (2) |
| **CKD by Creatinine Criteria** | 12,421 (3) | 14,534 (4) | 10,940 (3) |
| CKD by Creatinine Criteria and no recent AKI episode^a^ | 10,354 (3) | 12,267 (3) | 9,020 (3) |
| CKD by Creatinine Criteria, Recovered recent AKI on Admission | 1,615 (0.5) | 1,749 (0.5) | 1,529 (0.4) |
| CKD by Creatinine Criteria, Non-recovered recent AKI (AKD) on Admission | 452 (0.1) | 518 (0.1) | 391 (0.1) |
| **CKD after kidney transplant** | 4,672 (1) | 4,672 (1) | 4,672 (1) |
| CKD after kidney transplant and no recent AKI episode^a^ | 3,162 (1) | 3,162 (1) | 3,162 (1) |
| CKD after kidney transplant, Recovered recent AKI on Admission | 519 (0.1) | 480 (0.1) | 534 (0.2) |
| CKD after kidney transplant, Non-recovered recent AKI (AKD) on Admission | 991 (0.3) | 1,030 (0.3) | 976 (0.3) |
| **CKD G Stages among all encounters with CKD** | 84,216 (23) | 86,329 (24) | 82,735 (23) |
| eGFR, ml/min/1.73m^2^), median (25^th^, 75^th^) | 65.67 (47.8, 88.3) | 63.55 (46, 85) | 66.59 (48.8, 89.6) |
| eGFR, ml/min/1.73m^2^), mean (SD) | 69.5 (30) | 66.62 (27) | 69.42 (28) |
| G1 (eGFR ≥ 90 ml/min/1.73m^2^) | 19,660 (23) | 17,511 (20) | 20,176 (24) |
| G2 (90>eGFR ≥ 60 ml/min/1.73m^2^) | 28,803 (34) | 29,888 (35) | 28,637 (35) |
| G3a (60>eGFR ≥ 45 ml/min/1.73m^2^) | 16,925 (20) | 18,391 (21) | 16,381 (20) |
| G3b (45>eGFR ≥ 30 ml/min/1.73m^2^) | 11,796 (14) | 12,772 (15) | 10,903 (13) |
| G4 (30>eGFR ≥ 15 ml/min/1.73m^2^) | 5,382 (6) | 5,981 (7) | 5,032 (6) |
| G5 (eGFR < 15 ml/min/1.73m^2^) | 846 (1) | 974 (1) | 802 (1) |
| No staging can be done | 804 (1) | 812 (1) | 804 (1) |
| **CKD A Stages among all encounters with CKD (using our method)** |  |  |  |
| A1 | 23,707 (28) | 24,559 (28) | 23,291 (28) |
| A2 | 18,565 (22) | 19,003 (22) | 18,300 (22) |
| A3 | 6,092 (6) | 6,135 (7) | 6,057 (7) |
| No staging can be done | 35,852 (42) | 36,632 (42) | 35,087 (42) |
| **CKD A Stages among all encounters with CKD (using formula by Sumida et al)** |  |  |  |
| A1 | 29,508 (35) | 30,538 (35) | 28,920 (35) |
| A2 | 10,566 (13) | 10,792 (13) | 10,488 (13) |
| A3 | 8,290 (10) | 8,367 (10) | 8,240 (10) |
| No staging can be done | 35,852 (42) | 36,632 (42) | 35,087 (42) |

Abbreviations: AKD, acute kidney disease; CKD, chronic kidney disease; eGFR, estimated glomerular filtration rate.

^a^ Recent AKI episode defined by the presence of ICD9 or 10 codes documented in EHR in the three months prior to admission.
