# Supplementary material for: Development and validation of a race-agnostic computable phenotype for kidney health in adult hospitalized patients
Source: PLoS One. 2024 Apr 23;19(4):e0299332. doi: 10.1371/journal.pone.0299332 (PMC11037544; doi:10.1371/journal.pone.0299332)
Supplement: S17 Table — (DOCX) [file pone.0299332.s018.docx]

**S17 Table. AKI characteristics using race-adjusted and race-agnostic algorithms**

|  | **Using race-adjusted algorithm** | **Using race-agnostic algorithm 1** | **Using race-agnostic algorithm 2** |
| --- | --- | --- | --- |
| **Number of encounters, n** | 358,580 | 358,580 | 358,580 |
| **No AKI during hospitalization, n (%)** | 304,749 (85) | 304,174 (85) | 304,909 (85) |
| **AKI during hospitalization, n (%)** | 53,831 (15) | 54,406 (15) | 53,671 (15) |
| Reference serum creatinine, median (25^th^, 75^th^) | 0.85 (0.7, 1.1) | 0.84 (0.7 1.1) | 0.89 (0.7, 1.2) |
| Reference serum creatinine, mean (SD) | 1.03 (0.8) | 1.03 (0.8) | 1.07 (0.8) |
| **Maximum AKI Stage, n (%)** | 53,831 | 54,406 | 53,671 |
| Stage 1 | 36,062 (67) | 36,396 (66) | 36,001 (67) |
| Stage 2 | 9,403 (17) | 9,588 (18) | 9,316 (17) |
| Stage 3 (with or without KRT) | 8,366 (16) | 8,422 (15) | 8,354 (16) |
| KRT, n (%) | 2,058 (4) | 2,058 (4) | 2,058 (4) |
| Number of days on KRT, median (25^th^, 75^th^) | 10 (5, 20) | 10 (5, 20) | 10 (5, 20) |
| Recurrent AKI^a^, n (%) | 6,460 (12) | 6,477 (12) | 6,471 (12) |
| AKI duration, days, median (25^th^, 75^th^) | 2 (1, 4) | 2 (1, 4) | 2 (1, 4) |
| **AKI trajectories, n (%)** |  |  |  |
| Rapidly reversed AKI | 31,291 (58) | 31,605 (58) | 31,784 (59) |
| Persistent AKI | 22,540 (42) | 22,801 (42) | 21,887 (41) |

Abbreviations: AKI, acute kidney injury; KRT, kidney replacement therapy.

^a^ Recurrent AKI is defined as having more than one AKI episode during the hospital encounter.
