# Supplementary material for: Development and validation of a race-agnostic computable phenotype for kidney health in adult hospitalized patients
Source: PLoS One. 2024 Apr 23;19(4):e0299332. doi: 10.1371/journal.pone.0299332 (PMC11037544; doi:10.1371/journal.pone.0299332)
Supplement: S18 Table — (DOCX) [file pone.0299332.s019.docx]

**S18 Table. Comparison of performance of chronic kidney disease (CKD) and acute kidney injury (AKI) phenotyping algorithms, using race-adjusted algorithm, to manual chart review in diagnosing CKD and AKI.**

|  | **Manual chart review for CKD** | | | **Manual chart review for AKI** | | |
| --- | --- | --- | --- | --- | --- | --- |
| ***eKidneyHealth* Phenotyping Algorithm** | **Case** | **Control** | **Total** | **Case** | **Control** | **Total** |
| Case, n | 131 | 19^a^ | 150 | 202 | 2^b^ | 204 |
| Control, n | 1^b^ | 149 | 150 | 2^c^ | 94 | 96 |
| Total, n | 132 | 168 | 300 | 204 | 96 | 300 |
| Positive predictive value (95% Confidence Interval) |  |  | 72% (63%, 80%) |  |  | 89% (68%, 97%) |
| Negative predictive value (95% Confidence Interval) |  |  | 100% (98%, 100%) |  |  | 100% (99%, 100%) |
| Sensitivity (95% Confidence Interval) |  |  | 99% (96%, 100%) |  |  | 99% (97%, 100%) |
| Specificity (95% Confidence Interval) |  |  | 89% (83%, 93%) |  |  | 98% (93%, 100%) |
| Accuracy (95% Confidence Interval) |  |  | 91% (87%, 94%) |  |  | 98% (96%, 99%) |

Reasons for mismatches between phenotyping algorithm and manual chart review includes:

^a^ Assignment of wrong ICD code for patient who had AKI (n=4), assignment of wrong ICD code (n=2), assignment of wrong ICD code for nephrotic syndrome (n=4), and non-specific CKD code for patient who had AKI (n=9)

^b^ CKD by creatinine criteria was missed by algorithm (n=1)

^c^ Reference creatinine wrong based on erroneous laboratory measurement (n=2)

^d^ Wrong reference creatinine due to insufficient creatinine history for CKD patient (n=1) and wrong reference creatinine due to wrong CKD code assignment (n=1)
