# Supplementary material for: Development and validation of a race-agnostic computable phenotype for kidney health in adult hospitalized patients
Source: PLoS One. 2024 Apr 23;19(4):e0299332. doi: 10.1371/journal.pone.0299332 (PMC11037544; doi:10.1371/journal.pone.0299332)
Supplement: S19 Table — (DOCX) [file pone.0299332.s020.docx]

**S19 Table. Comparison of performance of chronic kidney disease (CKD) and acute kidney injury (AKI) phenotyping algorithms, using race agnostic algorithm 1, to manual chart review in diagnosing CKD and AKI.**

|  | **Manual chart review for CKD** | | | **Manual chart review for AKI** | | |
| --- | --- | --- | --- | --- | --- | --- |
| ***eKidneyHealth* Phenotyping Algorithm** | **Case** | **Control** | **Total** | **Case** | **Control** | **Total** |
| Case, n | 132 | 21^a^ | 153 | 202 | 2^b^ | 204 |
| Control, n | 0 | 147 | 147 | 2^c^ | 94 | 96 |
| Total, n | 132 | 168 | 300 | 204 | 96 | 300 |
| Positive predictive value (95% Confidence Interval) |  |  | 71% (62%, 78%) |  |  | 89% (68%, 97%) |
| Negative predictive value (95% Confidence Interval) |  |  | 100% (NA, NA) |  |  | 100% (99%, 100%) |
| Sensitivity (95% Confidence Interval) |  |  | 100% (97%, 100%) |  |  | 99% (97%, 100%) |
| Specificity (95% Confidence Interval) |  |  | 88% (82%, 92%) |  |  | 98% (93%, 100%) |
| Accuracy (95% Confidence Interval) |  |  | 90% (86%, 93%) |  |  | 98% (96%, 99%) |

Reasons for mismatches between phenotyping algorithm and manual chart review includes:

^a^ Assignment of wrong ICD code for patient who had AKI (n=4), assignment of wrong ICD code (n=2), assignment of wrong ICD code for nephrotic syndrome (n=4), non-specific CKD code for patient who had AKI (n=9), and CKD captured based on creatinine criteria by algorithm (n=2)

^b^ Reference creatinine wrong based on erroneous laboratory measurement (n=2)

^c^ Wrong reference creatinine due to insufficient creatinine history for CKD patient (n=1) and wrong reference creatinine due to wrong CKD code assignment (n=1)
