# Supplementary material for: Development and validation of a race-agnostic computable phenotype for kidney health in adult hospitalized patients
Source: PLoS One. 2024 Apr 23;19(4):e0299332. doi: 10.1371/journal.pone.0299332 (PMC11037544; doi:10.1371/journal.pone.0299332)
Supplement: S20 Table — (DOCX) [file pone.0299332.s021.docx]

**S20 Table. Comparison of reference creatinine determination methods used for race-agnostic algorithms to race-adjusted phenotyping algorithms among African American patients**

|  | | **Reference creatinine determination method using race-adjusted algorithm** | | | | |
| --- | --- | --- | --- | --- | --- | --- |
|  |  | Admission creatinine (n=45,200, 52%) | Minimum creatinine in the 7 days prior to admission (n=5,918, 7%) | Median creatinine in 8-365 days prior to admission (n=29,430, 34%) | Estimated creatinine ^a^ (n=5,614, 6%) | Missing (n=217, 0.3%) |
| **Reference creatinine determination method using race-agnostic algorithm 1** | Admission creatinine (n=41,285, 48%) | 41,100 (100) | 0 (0) | 0 (0) | 185 (0.4) | 0 (0) |
|  | Minimum creatinine in the 7 days prior to admission (n=5,595, 6%) | 0 (0) | 5,578 (100) | 0 (0) | 17 (0.3) | 0 (0) |
|  | Median creatinine in 8-365 days prior to admission (n=27,630, 32%) | 0 (0) | 0 (0) | 27,424 (99) | 206 (1) | 0 (0) |
|  | Estimated creatinine ^b^ (n=11,644, 13%) | 4,100 (35) | 340 (3) | 2,006 (17) | 5,198 (45) | 0 (0) |
|  | Missing (n=225, 0.3%) | 0 (0) | 0 (0) | 0 (0) | 8 (4) | 217 (96) |
| **Reference creatinine determination method using race-agnostic algorithm 2** | Admission creatinine (n=43,211, 50%) | 43,094 (100) | 0 (0) | 0 (0) | 117 (0.3) | 0 (0) |
|  | Minimum creatinine in the 7 days prior to admission (n=5,730, 7%) | 0 (0) | 5,720 (100) | 0 (0) | 10 (0.2) | 0 (0) |
|  | Median creatinine in 8-365 days prior to admission (n=28,522, 33%) | 0 (0) | 0 (0) | 28,397 (100) | 125 (0.4) | 0 (0) |
|  | Estimated creatinine ^c^ (n=8,693, 10%) | 2,106 (24) | 198 (2) | 1033 (11) | 5356 (61) | 0 (0) |
|  | Missing (n=223, 0.3%) | 0 (0) | 0 (0) | 0 (0) | 6 (4) | 217 (96) |

Percentages inside the table represents row percentages.

^a^ Estimated creatinine refers to the estimated creatinine calculated by back-calculation from the original Modification of Diet in Renal Disease Study (MDRD) equation with race multiplier.

^b^ Estimated creatinine refers to the estimated creatinine calculated by back-calculation from the MDRD equation without race multiplier.

^c^ Estimated creatinine refers to the estimated creatinine calculated by back-calculation from the 2021 CKD-EPI refit without race.
