# Supplementary material for: Development and validation of a race-agnostic computable phenotype for kidney health in adult hospitalized patients
Source: PLoS One. 2024 Apr 23;19(4):e0299332. doi: 10.1371/journal.pone.0299332 (PMC11037544; doi:10.1371/journal.pone.0299332)
Supplement: S21 Table — (DOCX) [file pone.0299332.s022.docx]

**S21** **Table. CKD status and G-stages for African American encounters using race-adjusted and race-agnostic algorithms**

|  | **Using race-adjusted algorithm** | **Using race-agnostic algorithm 1** | **Using race-agnostic algorithm 2** |
| --- | --- | --- | --- |
| **Number of encounters for African American cohort, n** | N = 86,379 | N = 86,379 | N=86,379 |
| eGFR, ml/min/1.73m^2^), median (25^th^, 75^th^) | 108.5 (84.9, 129.3) | 96.1 (77.6, 113.2) | 98.89 (79.9, 114.8) |
| eGFR, ml/min/1.73m^2^), mean (SD) | 105.5 (33.1) | 92.5 (29.2) | 94.67 (27.8) |
| Increase in eGFR after race adjustment, ml/min/1.73m^2^), median (25^th^, 75^th^) | NA | 15.31 (12.4, 18.0) | 11.3 (8.1, 15.4) |
| Increase in eGFR after race adjustment, ml/min/1.73m^2^), mean (SD) | NA | 14.74 (4.6) ^a^ | 11.96 (5.8) ^b^ |
| **CKD class** |  |  |  |
| Insufficient Data (No CKD with warning), n (%) | 5 (0.0) | 5 (0.0) | 5 (0.0) |
| No CKD, n (%) | 61,579 (71) | 59,466 (69) | 60,371 (70) |
| CKD, n (%) | 24,795 (29) | 26,908 (31) | 26,003 (30) |
| G1 (eGFR ≥ 90 ml/min/1.73m^2^), n (%) | 8,141 (33) | 5,992 (22) | 6,791 (26) |
| G2 (90>eGFR ≥ 60 ml/min/1.73m^2^), n (%) | 7,986 (32) | 9,071 (34) | 8,802 (34) |
| G3a (60>eGFR ≥ 45 ml/min/1.73m^2^), n (%) | 3,934 (16) | 5,400 (20) | 4,752 (18) |
| G3b (45>eGFR ≥ 30 ml/min/1.73m^2^), n (%) | 2,860 (12) | 3,836 (14) | 3,405 (13) |
| G4 (30>eGFR ≥ 15 ml/min/1.73m^2^), n (%) | 1,420 (6) | 2,019 (8) | 1,733 (6) |
| G5 (eGFR < 15 ml/min/1.73m^2^), n (%) | 237 (1) | 365 (1) | 297 (1) |
| No staging can be done, n (%) | 217 (1) | 225 (1) | 223 (1) |
| eGFR among CKD patients | N = 24,795 | N = 26,908 | N= 26,003 |
| eGFR, ml/min/1.73m^2^), median (25^th^, 75^th^) | 72.8 (51.9, 101.1) | 64.3 (46.3, 87) | 67.4 (48.6, 91.3) |
| eGFR, ml/min/1.73m^2^), mean (SD) | 77.5 (34.7) | 67.5 (29.2) | 70.2 (29.3) |
| Increase in eGFR after race adjustment, ml/min/1.73m^2^), median (25^th^, 75^th^) | NA | 9.9 (7.1, 13.8) ^a^ | 6.3 (4.2, 9.6) ^b^ |
| Increase in eGFR after race adjustment, ml/min/1.73m^2^), mean (SD) | NA | 10.6 (4.7) ^a^ | 7.7 (5.2) ^b^ |

Abbreviations: eGFR, estimated glomerular filtration rate.

Race-agnostic algorithm 1 calculates estimated creatinine by back-calculation from the Modification of Diet in Renal Disease Study equation without race multiplier. Race-agnostic algorithm 2 calculates estimated creatinine by back calculation from the 2021 CKD-EPI fit without race. Race-adjusted algorithm calculates estimated creatinine by back-calculation from the original Modification of Diet in Renal Disease Study equation with race multiplier.

^a^ Increase in eGFR after race adjustment is calculated by finding difference between eGFR obtained using race-adjusted algorithm and eGFR obtained using race-agnostic algorithm 1.

^b^ Increase in eGFR after race adjustment is calculated by finding difference between eGFR obtained using race-adjusted algorithm and eGFR obtained using race-agnostic algorithm 2.
