# Supplementary material for: Development and validation of a race-agnostic computable phenotype for kidney health in adult hospitalized patients
Source: PLoS One. 2024 Apr 23;19(4):e0299332. doi: 10.1371/journal.pone.0299332 (PMC11037544; doi:10.1371/journal.pone.0299332)
Supplement: S22 Table — (DOCX) [file pone.0299332.s023.docx]

**S22 Table. Reclassification of CKD status and CKD stages,** **using race agnostic algorithm 1, among African American patients after race-adjustment**

|  | | | **CKD G-stage using race-adjusted algorithm** | | | | | | | | |
| --- | --- | --- | --- | --- | --- | --- | --- | --- | --- | --- | --- |
|  |  |  | No CKD (n=61,579, 71%) | CKD (n=24,795, 29%) | G1  (n=8,141, 33%) | G2 (n=7,986, 32%) | G3a (n=3,934, 16%) | G3b (n=2,860, 11%) | G4 (n=1,420, 6%) | G5 (n=237, 1%) | No staging (n=217, 1%) |
| **CKD**  **G-stage using race-agnostic algorithm 1** | No CKD (n= 59,466, 69%) | 59,466 (100) | | 0 (0) | 0 (0) | 0 (0) | 0 (0) | 0 (0) | 0 (0) | 0 (0) | 0 (0) |
|  | CKD (n=26,908, 31%) | 2,113 (8) | | 24,795 (92) | 8,141 (33) | 7,986 (32) | 3,934 (16) | 2,860 (12) | 1,420 (6) | 237 (1) | 217 (1) |
|  | G1 (n= 5,992, 22%) | 388 (6) | | 5,604 (94) | 5,604 (100) | 0 (0) | 0 (0) | 0 (0) | 0 (0) | 0 (0) | 0 (0) |
|  | G2 (n= 9,071, 34%) | 1,380 (15) | | 7,691 (85) | 2,537 (33) | 5,154 (67) | 0 (0) | 0 (0) | 0 (0) | 0 (0) | 0 (0) |
|  | G3a (n= 5,400, 20%) | 313 (6) | | 5,087 (94) | 0 (0) | 2,832 (56) | 2,255 (44) | 0 (0) | 0 (0) | 0 (0) | 0 (0) |
|  | G3b (n= 3,836, 14%) | 22 (1) | | 3,814 (99) | 0 (0) | 0 (0) | 1,679 (44) | 2,135 (56) | 0 (0) | 0 (0) | 0 (0) |
|  | G4 (n= 2,019, 8%) | 2 (0.1) | | 2,017 (99) | 0 (0) | 0 (0) | 0 (0) | 725 (36) | 1,292 (64) | 0 (0) | 0 (0) |
|  | G5 (n= 365, 1%) | 0 (0) | | 365 (100) | 0 (0) | 0 (0) | 0 (0) | 0 (0) | 128 (35) | 237 (65) | 0 (0) |
|  | No staging (n= 225, 1%) | 8 (4) | | 217 (96) | 0 (0) | 0 (0) | 0 (0) | 0 (0) | 0 (0) | 0 (0) | 217 (100) |

Percentages inside the table represents row percentages.

Abbreviations: CKD, chronic kidney disease.

Gray shading indicates patients who were reclassified into no CKD or less severe stages of CKD after race adjustment.

Race-adjusted algorithm calculated eGFR using 2009 CKD-EPI formula, while race-agnostic algorithm 1 used 2009 CKD-EPI formula with race modifier removed. Race-agnostic algorithm 2 calculated eGFR using the 2021 CKD-EPI refit without race.
