# Supplementary material for: Development and validation of a race-agnostic computable phenotype for kidney health in adult hospitalized patients
Source: PLoS One. 2024 Apr 23;19(4):e0299332. doi: 10.1371/journal.pone.0299332 (PMC11037544; doi:10.1371/journal.pone.0299332)
Supplement: S23 Table — (DOCX) [file pone.0299332.s024.docx]

**S23 Table: Reclassification of CKD stages among African-American CKD patients identified by medical history using into less severe CKD stages after race adjustment**

|  | | **CKD G-stage using race-adjusted algorithm** | | | | | | | |
| --- | --- | --- | --- | --- | --- | --- | --- | --- | --- |
|  |  | CKD (n= 23,284, 100%) | G1 (n=7,610, 33%) | G2 (n= 7,264, 31%) | G3a (n= 3,743, 15%) | G3b (n=2,808, 12%) | G4 (n= 1,416, 6%) | G5 (n= 235, 1%) | No staging (n= 208, 1%) |
| **CKD**  **G-stage using race-agnostic algorithm 1** | CKD (n= 23,284, 100%) | 23,284 (100) | 7,610 (33) | 7,264 (31) | 3,473 (15) | 2,808 (12) | 1,416 (6) | 235 (1) | 208 (1) |
|  | G1 (n= 5,319, 23%) | 5,319 (100) | 5,319 (100) | 0 (0) | 0 (0) | 0 (0) | 0 (0) | 0 (0) | 0 (0) |
|  | G2 (n= 6,981, 30%) | 6,981 (100) | 2,291 (33) | 4,690 (67) | 0 (0) | 0 (0) | 0 (0) | 0 (0) | 0 (0) |
|  | G3a (n= 4,679, 20%) | 4,679 (100) | 0 (0) | 2,574 (55) | 2,105 (45) | 0 (0) | 0 (0) | 0 (0) | 0 (0) |
|  | G3b (n= 3,727, 16%) | 3,727 (100) | 0 (0) | 0 (0) | 1,638 (44) | 2,089 (56) | 0 (0) | 0 (0) | 0 (0) |
|  | G4 (n= 2,007, 9%) | 2,007 (100) | 0 (0) | 0 (0) | 0 (0) | 719 (36) | 1,288 (64) | 0 (0) | 0 (0) |
|  | G5 (n= 363, 2%) | 363 (100) | 0 (0) | 0 (0) | 0 (0) | 0 (0) | 128 (35) | 235 (65) | 0 (0) |
|  | No staging (n= 208, 1%) | 208 (100) | 0 (0) | 0 (0) | 0 (0) | 0 (0) | 0 (0) | 0 (0) | 208 (100) |
| **CKD**  **G-stage using race-agnostic algorithm 2** | CKD (n= 23,284, 100%) | 23,284 (100) | 7,610 (33) | 7,264 (31) | 3,743 (15) | 2,808 (12) | 1,416 (6) | 235 (1) | 208 (1) |
|  | G1 (n= 6,124, 26%) | 6,124 (100) | 6,124 (100) | 0 (0) | 0 (0) | 0 (0) | 0 (0) | 0 (0) | 0 (0) |
|  | G2 (n= 7,274, 31%) | 7,274 (100) | 1,486 (20) | 5,788 (80) | 0 (0) | 0 (0) | 0 (0) | 0 (0) | 0 (0) |
|  | G3a (n= 4,318, 19%) | 4,318 (100) | 0 (0) | 1,476 (34) | 2,842 (66) | 0 (0) | 0 (0) | 0 (0) | 0 (0) |
|  | G3b (n= 3,339, 14%) | 3,339 (100) | 0 (0) | 0 (0) | 901 (27) | 2,438 (73) | 0 (0) | 0 (0) | 0 (0) |
|  | G4 (n= 1,726, 8%) | 1,726 (100) | 0 (0) | 0 (0) | 0 (0) | 370 (21) | 1,356 (79) | 0 (0) | 0 (0) |
|  | G5 (n= 295, 1%) | 295 (100) | 0 (0) | 0 (0) | 0 (0) | 0 (0) | 60 (20) | 235 (80) | 0 (0) |
|  | No staging (n= 208, 1%) | 208 (100) | 0 (0) | 0 (0) | 0 (0) | 0 (0) | 0 (0) | 0 (0) | 208 (100) |

Percentages inside the table represents row percentages.

Abbreviations. CKD, chronic kidney disease.

Gray shading indicates patients who were reclassified into less severe stages of CKD patients after inclusion of race multiplier.

Race-adjusted algorithm calculated eGFR using 2012 CKD-EPI formula while race-agnostic algorithm 1 used 2012 CKD-EPI formula with race modifier removed. Race-agnostic algorithm 2 calculated eGFR using the 2021 CKD-EPI refit without race.
