# Supplementary material for: Development and validation of a race-agnostic computable phenotype for kidney health in adult hospitalized patients
Source: PLoS One. 2024 Apr 23;19(4):e0299332. doi: 10.1371/journal.pone.0299332 (PMC11037544; doi:10.1371/journal.pone.0299332)
Supplement: S24 Table — (DOCX) [file pone.0299332.s025.docx]

**S24 Table. Reclassification of CKD status and CKD stages after race adjustment among African American patients who do not have CKD by medical history**

|  | | **CKD G-stage using race-adjusted algorithms** | | | | | | | | |
| --- | --- | --- | --- | --- | --- | --- | --- | --- | --- | --- |
|  |  | **No CKD (n=61,579, 98%)** | **CKD (n=1,511, 2%)** | **G1 (n=531, 35%)** | **G2 (n=722, 48%)** | **G3a (n=191, 13%)** | **G3b (n= 52, 4%)** | **G4 (n= 4, 0.3%)** | **G5 (n= 2, 0.1%)** | **No staging (n= 9, 0.01%)** |
| **CKD**  **G-stage using race-agnostic algorithm 1** | No CKD (n=59,466, 94%) | 59,466 (100) | 0 (0) | 0 (0) | 0 (0) | 0 (0) | 0 (0) | 0 (0) | 0 (0) | 0 (0) |
|  | CKD (n= 3,624, 6%) | 2,113 (58) | 1,511 (42) | 531 (35) | 722 (48) | 191 (13) | 52 (3) | 4 (0.3) | 2 (0.1) | 9 (1) |
|  | G1 (n= 673, 19%) | 388 (58) | 285 (42) | 285 (42) | 0 (0) | 0 (0) | 0 (0) | 0 (0) | 0 (0) | 0 (0) |
|  | G2 (n= 2,090, 58%) | 1,380 (66) | 710 (34) | 246 (12) | 464 (22) | 0 (0) | 0 (0) | 0 (0) | 0 (0) | 0 (0) |
|  | G3a (n= 721, 20%) | 313 (43) | 408 (57) | 0 (0) | 258 (36) | 150 (21) | 0 (0) | 0 (0) | 0 (0) | 0 (0) |
|  | G3b (n= 109, 3%) | 22 (20) | 87 (80) | 0 (0) | 0 (0) | 41 (38) | 46 (42) | 0 (0) | 0 (0) | 0 (0) |
|  | G4 (n= 12, 0.3%) | 2 (17) | 10 (83) | 0 (0) | 0 (0) | 0 (0) | 6 (50) | 4 (33) | 0 (0) | 0 (0) |
|  | G5 (n= 2, 0.1%) | 0 (0) | 2 (100) | 0 (0) | 0 (0) | 0 (0) | 0 (0) | 0 (0) | 2 (100) | 0 (0) |
|  | No staging (n= 17, 0.5%) | 8 (47) | 9 (53) | 0 (0) | 0 (0) | 0 (0) | 0 (0) | 0 (0) | 0 (0) | 9 (53) |
| **CKD**  **G-stage using race-agnostic algorithm 2** | No CKD (n= 60,371, 96%) | 60,371 (100) | 0 (0) | 0 (0) | 0 (0) | 0 (0) | 0 (0) | 0 (0) | 0 (0) | 0 (0) |
|  | CKD (n= 2,719, 4%) | 1,208 (44) | 1,511 (56) | 531 (35) | 722 (48) | 191 (13) | 52 (3) | 4 (0.3) | 2 (0.1) | 9 (1) |
|  | G1 (n= 667, 25%) | 299 (44) | 368 (56) | 368 (100) | 0 (0) | 0 (0) | 0 (0) | 0 (0) | 0 (0) | 0 (0) |
|  | G2 (n= 1,528, 56%) | 779 (51) | 749 (51) | 163 (21) | 586 (79) | 0 (0) | 0 (0) | 0 (0) | 0 (0) | 0 (0) |
|  | G3a (n= 434, 16%) | 118 (27) | 316 (73) | 0 (0) | 136 (43) | 180 (57) | 0 (0) | 0 (0) | 0 (0) | 0 (0) |
|  | G3b (n=66, 2%) | 6 (9) | 60 (91) | 0 (0) | 0 (0) | 11 (18) | 49 (82) | 0 (0) | 0 (0) | 0 (0) |
|  | G4 (n= 7, 0.3%) | 0 (0) | 7 (100) | 0 (0) | 0 (0) | 0 (0) | 3 (42) | 4 (58) | 0 (0) | 0 (0) |
|  | G5 (n= 2, 0.1%) | 0 (0) | 2 (100) | 0 (0) | 0 (0) | 0 (0) | 0 (0) | 0 (0) | 2 (100) | 0 (0) |
|  | No staging (n= 15, 1%) | 6 (40) | 9 (60) | 0 (0) | 0 (0) | 0 (0) | 0 (0) | 0 (0) | 0 (0) | 9 (100) |

Percentages inside the table represents row percentages.

Abbreviations: CKD, chronic kidney disease.

Gray shading indicates patients who were reclassified into no CKD or less severe stages of CKD after race adjustment.

Race-adjusted algorithm calculated eGFR using 2009 CKD-EPI formula, while race-agnostic algorithm 1 used 2009 CKD-EPI formula with race modifier removed. Race-agnostic algorithm 2 calculated eGFR using the 2021 CKD-EPI refit without race.
