# Supplementary material for: Development and validation of a race-agnostic computable phenotype for kidney health in adult hospitalized patients
Source: PLoS One. 2024 Apr 23;19(4):e0299332. doi: 10.1371/journal.pone.0299332 (PMC11037544; doi:10.1371/journal.pone.0299332)
Supplement: S25 Table — (DOCX) [file pone.0299332.s026.docx]

**S25 Table. Reclassification of AKI status and AKI stages,** **using race agnostic algorithm 1, after race adjustment among African American patients**

|  | |  | **AKI stage with race adjustment** | | | |
| --- | --- | --- | --- | --- | --- | --- |
|  |  | **No AKI (n=74,503, 86%)** | **AKI (n=11,876, 14%)** | **Stage 1 (n=7,937, 67%)** | **Stage 2 (n=1,891, 16%)** | **Stage 3 (n=2,048, 17%)** |
| AKI stage using race-agnostic algorithm 1 | No AKI (n=73,928, 86%) | 73,912 (100) | 16 (0.02) | 15 (0.02) | 1 (0) | 0 (0) |
|  | AKI (n=12,451, 14%) | 591 (5) | 11,860 (95) | 7,922 (64) | 1890 (15) | 2,048 (16) |
|  | Stage 1 (n=8,271, 66%) | 591 (7) | 7,680 (93) | 7,676 (93) | 4 (0.1) | 0 (0) |
|  | Stage 2 (n=2,076, 17%) | 0 (0) | 2,076 (100) | 246 (12) | 1,827 (88) | 3 (0.1) |
|  | Stage 3 (n=2,104, 17%) | 0 (0) | 2,104 (100) | 0 (0) | 59 (3) | 2,045 (97) |

Percentages in the table represent row percentages.

Abbreviations. AKI, acute kidney injury.

Gray shading indicates patients who were reclassified into no AKI or less severe stages of AKI patients after race adjustment. Sixteen patients who were classified to more severe stages were due to change in CKD class from CKD to no CKD after race multiplier inclusion.

Reference creatinine used in determination of AKI stages involves calculation of an estimated creatinine for no CKD patients. Race-adjusted algorithm and race-agnostic algorithm calculate estimated creatinine by back-calculation from the Modification of Diet in Renal Disease Study equation with and without race multiplier, respectively. Race-agnostic algorithm 2 calculates estimated creatinine by back calculation from the 2021 CKD-EPI refit without race.
