# Supplementary material for: Development and validation of a race-agnostic computable phenotype for kidney health in adult hospitalized patients
Source: PLoS One. 2024 Apr 23;19(4):e0299332. doi: 10.1371/journal.pone.0299332 (PMC11037544; doi:10.1371/journal.pone.0299332)
Supplement: S26 Table — (DOCX) [file pone.0299332.s027.docx]

**S26 Table. AKI status and stages for African American encounters using algorithms without and with race adjustment**

|  | **Using race-adjusted algorithm**  **(N = 86,379)** | **Using race-agnostic algorithm 1**  **(N = 86,379)** | **Using race-agnostic algorithm 2**  **(N = 86,379)** |
| --- | --- | --- | --- |
| No AKI, n (%) | 74,503 (86) | 73,928 (85) | 74,128 (86) |
| AKI, n (%) | 11,876 (14) | 12,451 (14) | 12,251 (14) |
| **Worst AKI staging, n (%)** |  |  |  |
| Stage 1 | 7,937 (67) | 8,271 (66) | 8,159 (66) |
| Stage 2 | 1,891 (16) | 2,076 (17) | 2,006 (16) |
| Stage 3 | 2,048 (17) | 2,104 (17) | 2,086 (17) |

Reference creatinine used in determination of AKI stages involves calculation of an estimated creatinine for no CKD patients. Race-adjusted algorithm and race-agnostic algorithm calculate estimated creatinine by back-calculation from the Modification of Diet in Renal Disease Study equation with and without race multiplier, respectively. Race-agnostic algorithm 2 calculates estimated creatinine by back calculation from the 2021 CKD-EPI refit without race.
