# Supplementary figures and images for: Development and validation of a race-agnostic computable phenotype for kidney health in adult hospitalized patients
Source: PLoS One. 2024 Apr 23;19(4):e0299332. doi: 10.1371/journal.pone.0299332 (PMC11037544; doi:10.1371/journal.pone.0299332)

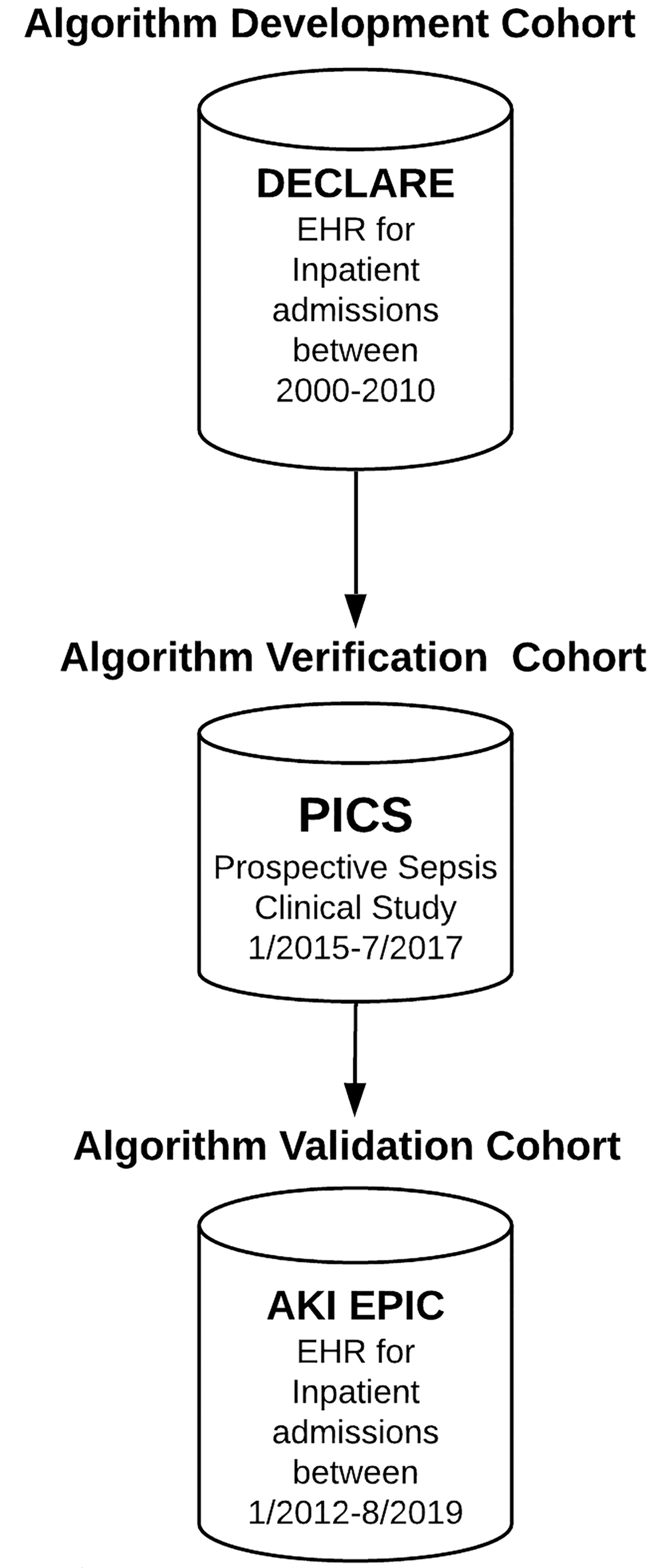

Supplement: S1 Fig — (TIF) [file pone.0299332.s028.tif]

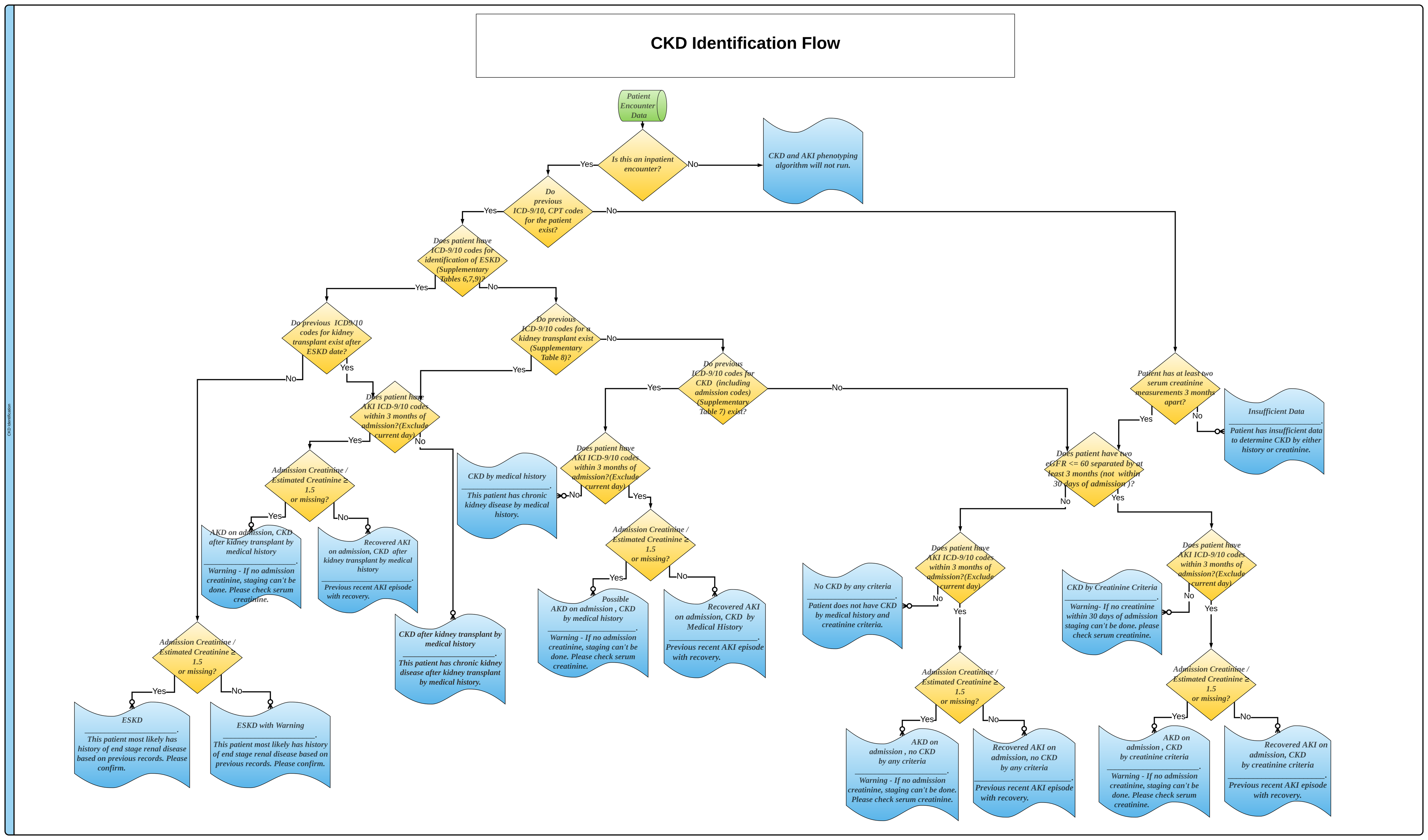

Supplement: S3 Fig — This flow shows the rules for determination of preadmission chronic kidney disease using data from the index admission along with historical data prior to that admission. (TIF) [file pone.0299332.s030.tif]

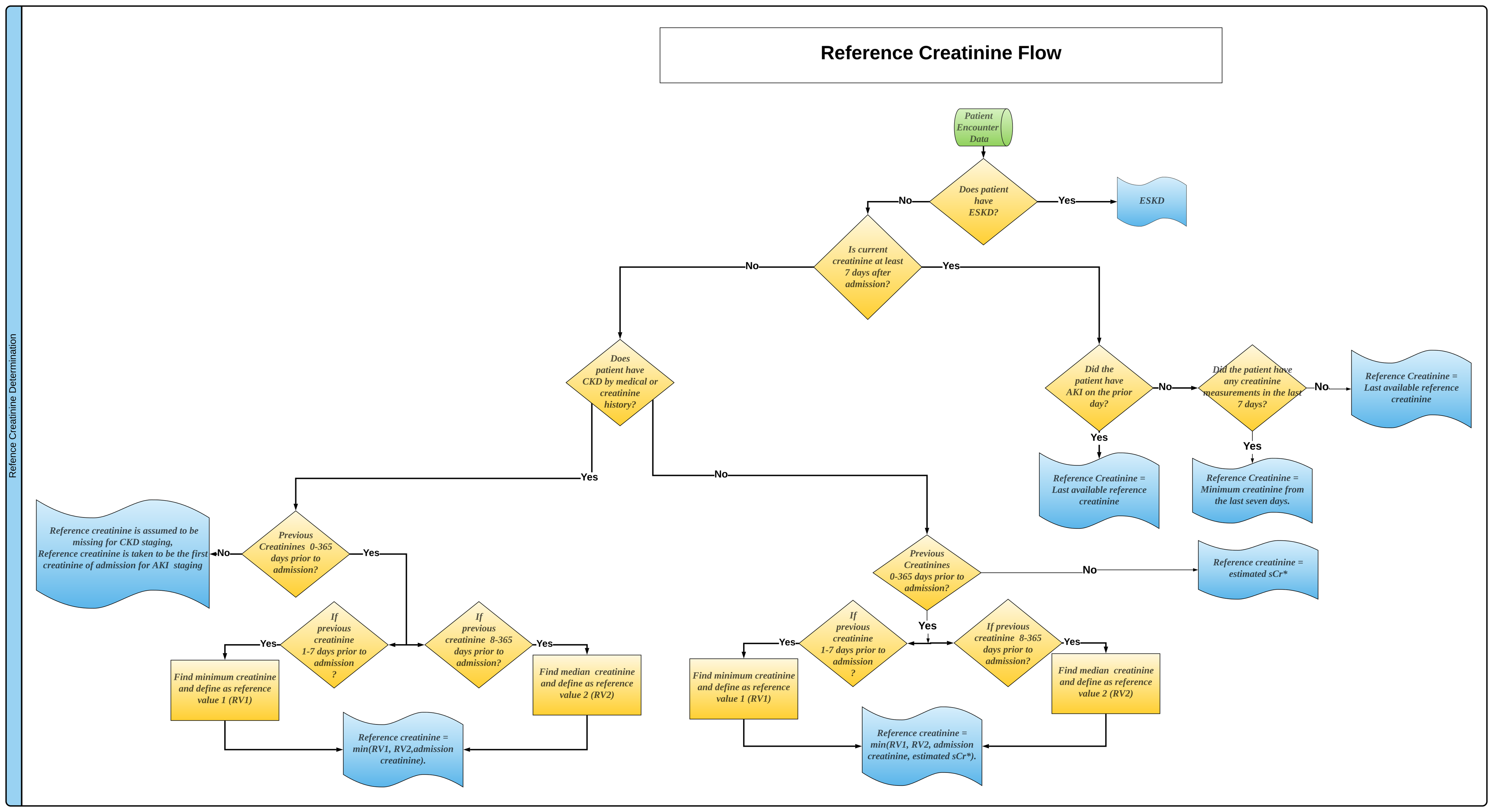

Supplement: S4 Fig — This flow shows the rule for determination of reference creatinine that changes dynamically during the index admission. *Race-adjusted algorithm and race-agnostic algorithm calculate estimated creatinine by back-calculation from the Modification of Diet in Renal Disease Study equation with and without race multiplier, respectively. Race-agnostic algorithm 2 calculates estimated creatinine by back calculation from the 2021 CKD-EPI refit without race. (TIF) [file pone.0299332.s031.tif]

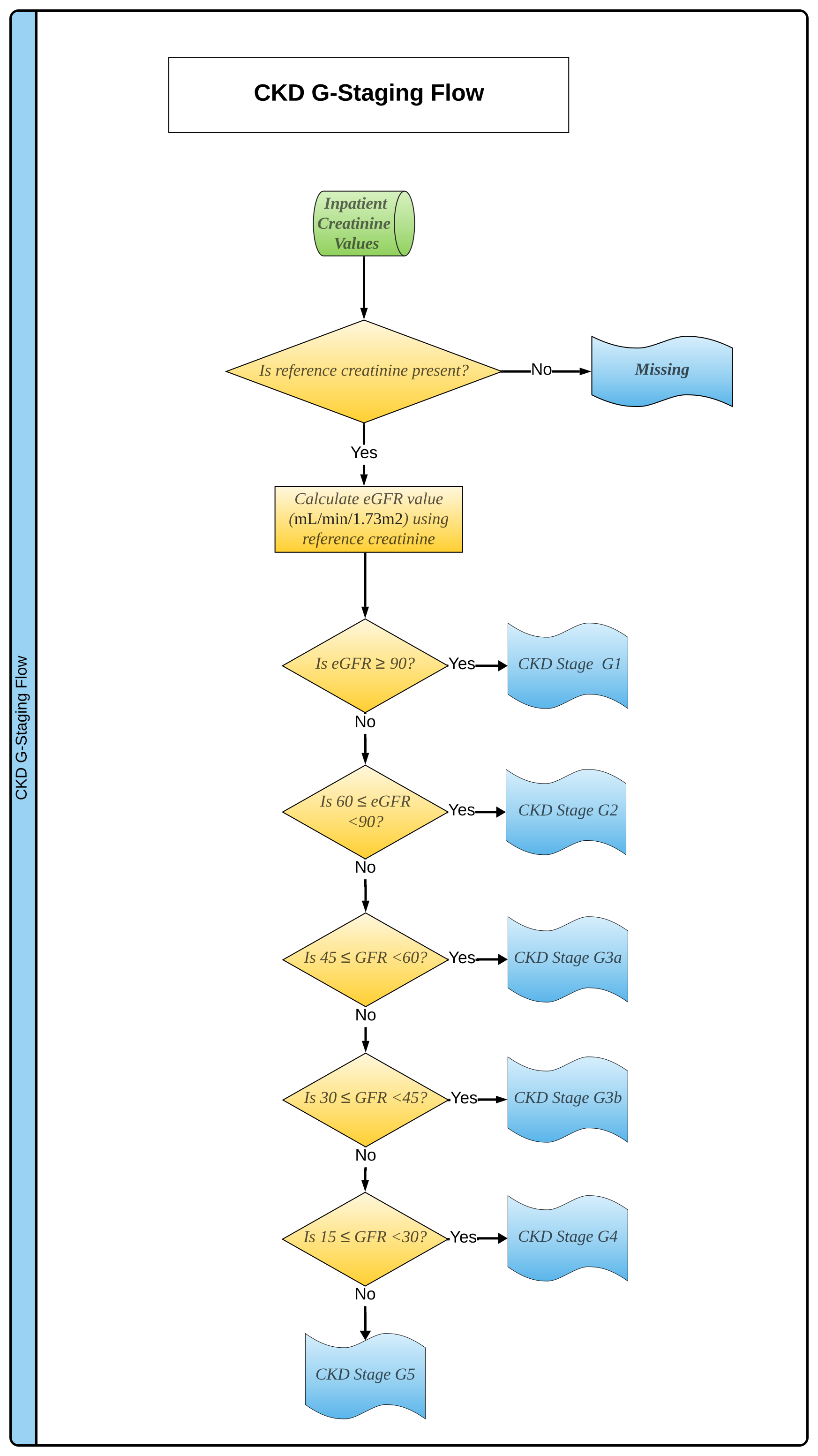

Supplement: S5 Fig — This flow shows rule for determination of G-stages for patients with chronic kidney disease. (TIF) [file pone.0299332.s032.tif]

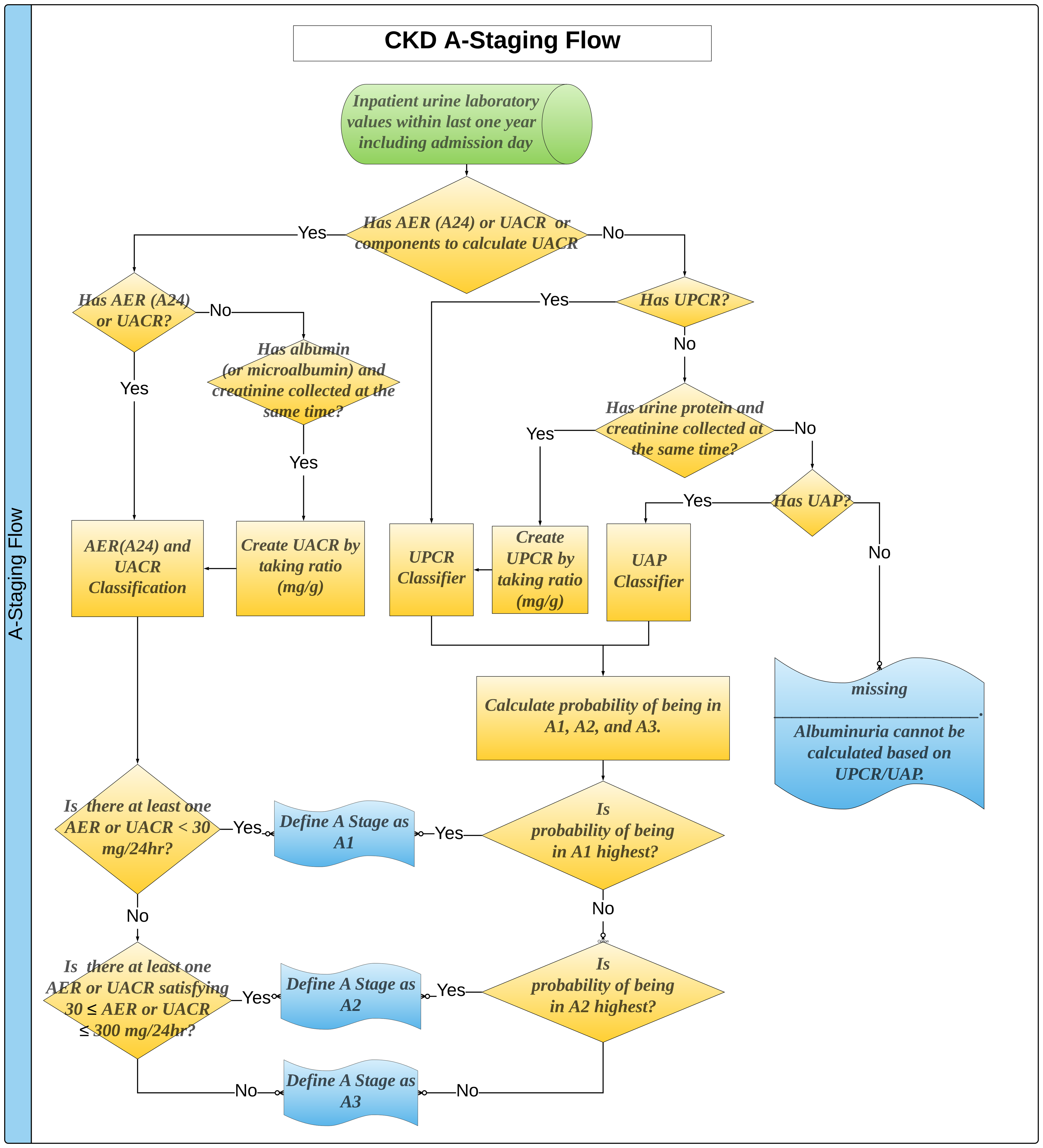

Supplement: S6 Fig — This flow shows rule for determination of A-stages for patients with chronic kidney disease. (TIF) [file pone.0299332.s033.tif]

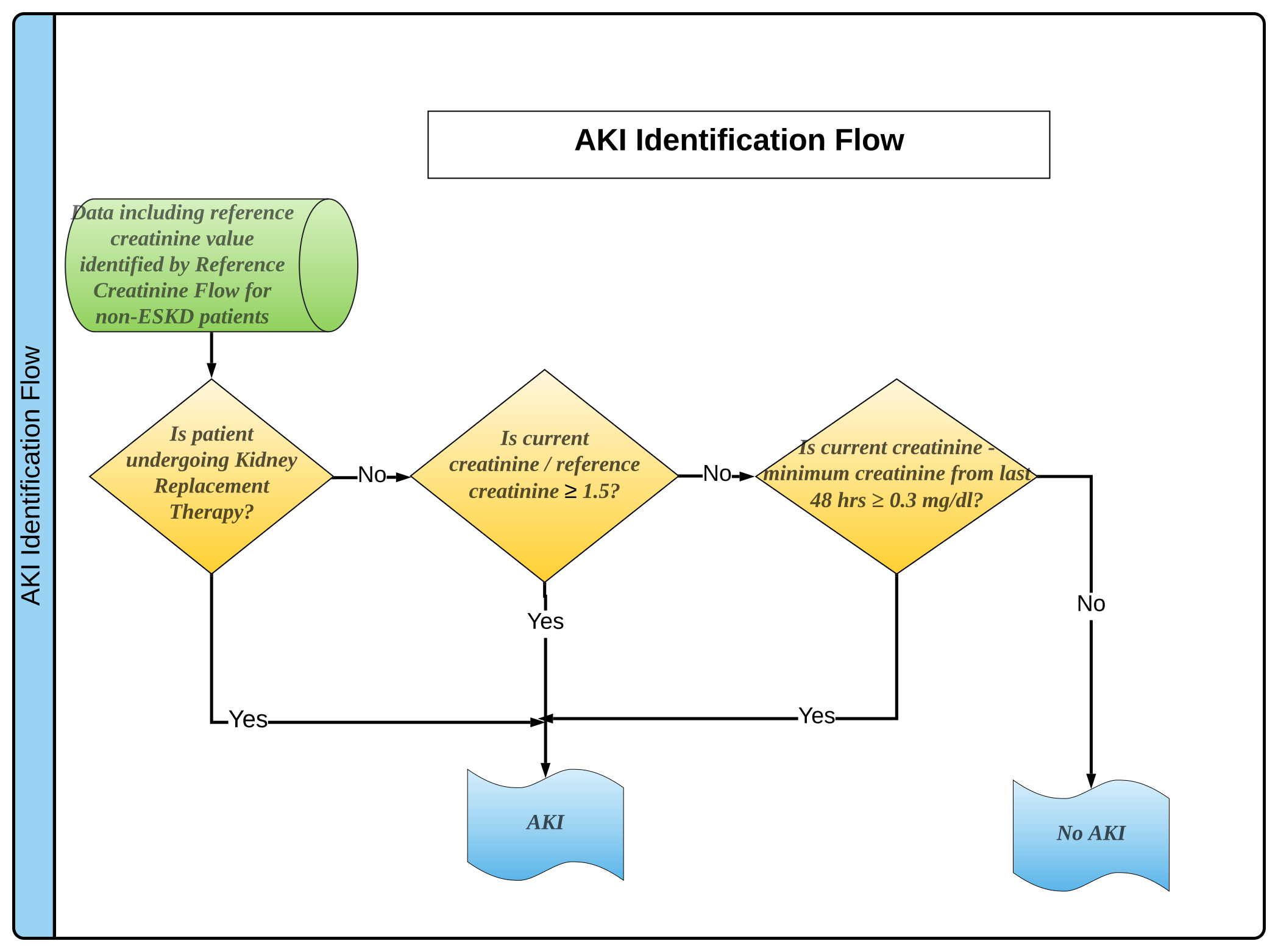

Supplement: S7 Fig — This flow shows rule for determination of type of kidney injury/disease during the index admission. (TIF) [file pone.0299332.s034.tif]

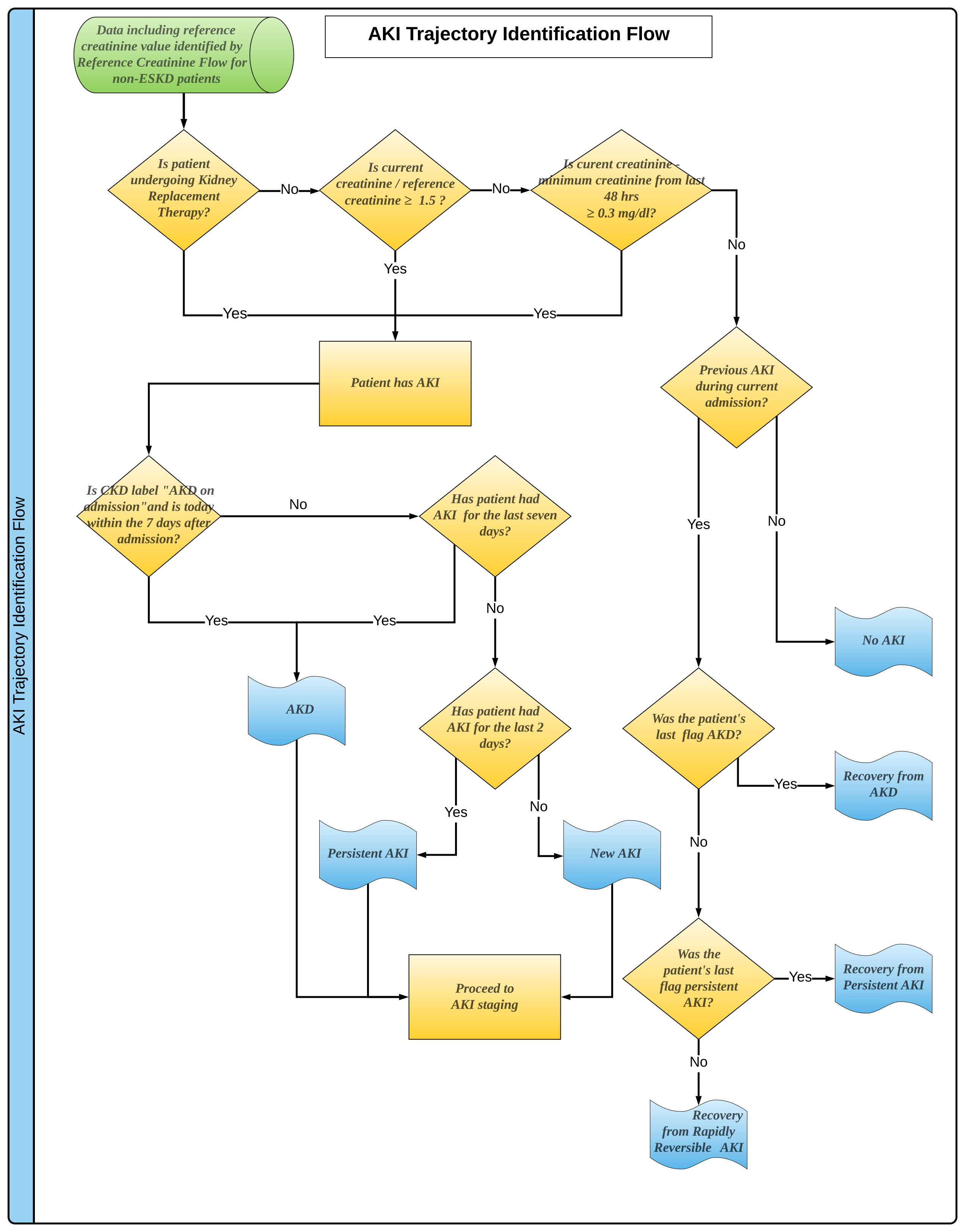

Supplement: S8 Fig — This flow shows rule for determination of type of kidney injury/disease during the index admission. (TIFF) [file pone.0299332.s035.tiff]

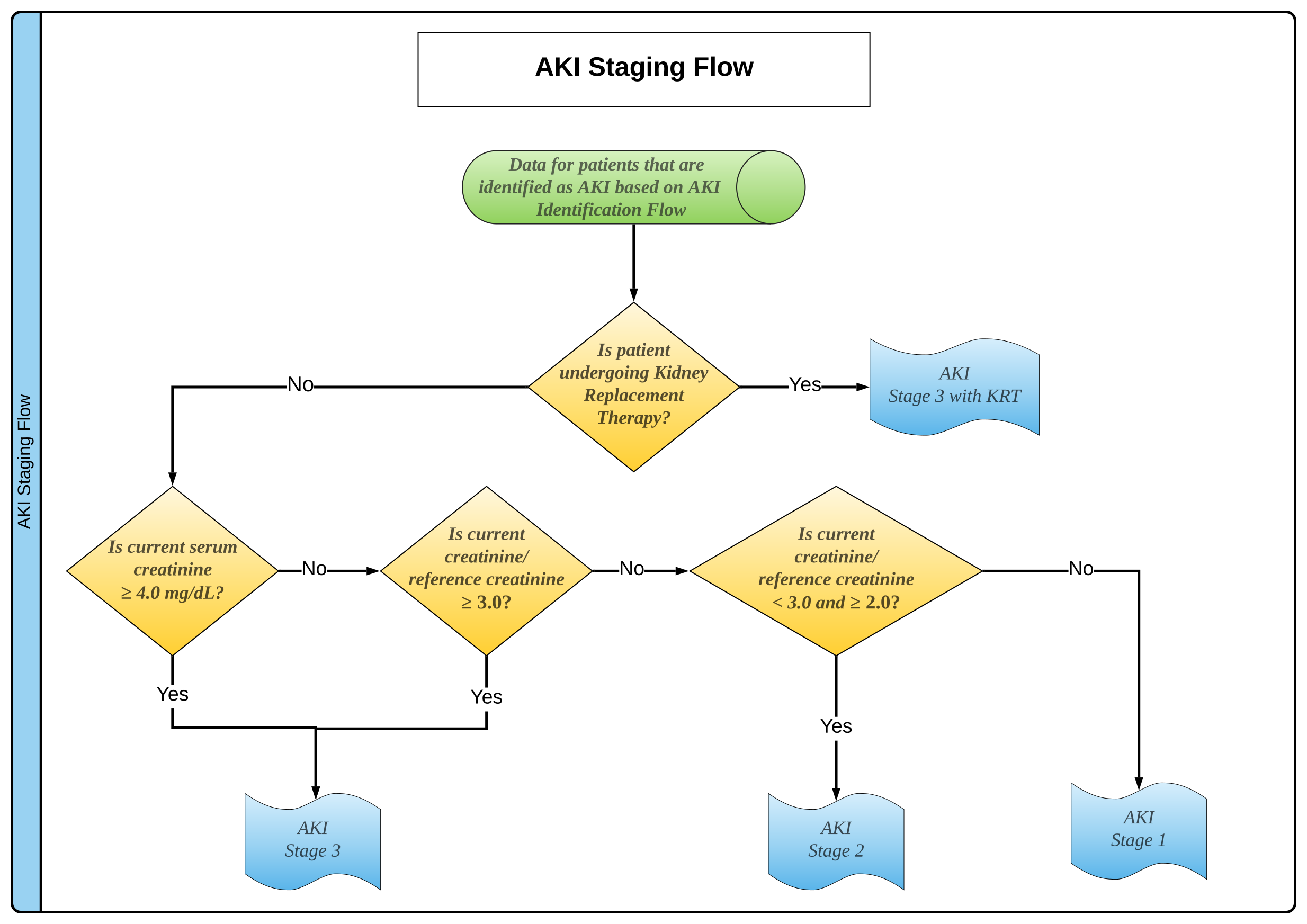

Supplement: S9 Fig — This flow shows rule for determination of AKI stages for patients with acute kidney injury using KDIGO criteria. (TIF) [file pone.0299332.s036.tif]
